# A Novel Graph Neural Network Methodology to Investigate Dihydroorotate Dehydrogenase Inhibitors in Small Cell Lung Cancer

**DOI:** 10.3390/biom11030477

**Published:** 2021-03-23

**Authors:** Hong-Yi Zhi, Lu Zhao, Cheng-Chun Lee, Calvin Yu-Chian Chen

**Affiliations:** 1 Artificial Intelligence Medical Center, School of Intelligent Systems Engineering, Sun Yat-sen University, Shenzhen 510275, China; zhihy@mail2.sysu.edu.cn (H.-Y.Z.); zhaolu26@mail.sysu.edu.cn (L.Z.); 2 Department of Clinical Laboratory, The Sixth Affiliated Hospital, Sun Yat-sen University, Guangzhou 510655, China; 3 Department of Medical Research, China Medical University Hospital, Taichung 40447, Taiwan; leeck@mail.cmu.edu.tw; 4 Department of Bioinformatics and Medical Engineering, Asia University, Taichung 41354, Taiwan

**Keywords:** dihydroorotate dehydrogenase, graph neural networks, deep learning, machine learning, molecular dynamics simulation

## Abstract

Small cell lung cancer (SCLC) is a particularly aggressive tumor subtype, and dihydroorotate dehydrogenase (DHODH) has been demonstrated to be a therapeutic target for SCLC. Network pharmacology analysis and virtual screening were utilized to find out related proteins and investigate candidates with high docking capacity to multiple targets. Graph neural networks (GNNs) and machine learning were used to build reliable predicted models. We proposed a novel concept of multi-GNNs, and then built three multi-GNN models called GIAN, GIAT, and SGCA, which achieved satisfactory results in our dataset containing 532 molecules with all R^^2^ values greater than 0.92 on the training set and higher than 0.8 on the test set. Compared with machine learning algorithms, random forest (RF), and support vector regression (SVR), multi-GNNs had a better modeling effect and higher precision. Furthermore, the long-time 300 ns molecular dynamics simulation verified the stability of the protein–ligand complexes. The result showed that ZINC8577218, ZINC95618747, and ZINC4261765 might be the potentially potent inhibitors for DHODH. Multi-GNNs show great performance in practice, making them a promising field for future research. We therefore suggest that this novel concept of multi-GNNs is a promising protocol for drug discovery.

## 1. Introduction

Small cell lung cancer (SCLC) is particularly aggressive and the most malignant subtype of lung cancer [1]. The clinical treatment of SCLC is mainly chemotherapy, but the choice of chemotherapy drugs is limited. In recent years, compared with the rapid development of targeted drugs for non-small cell lung cancer (NSCLC), research on targeted drugs for SCLC has developed slowly, and the difficulty is that no direct therapeutic target has been found. Therefore, it is of great significance to develop a treatment regimen for small cell lung cancer [2]. Li et al. used a CRISPR screening approach to identify a metabolic vulnerability and determined that the dihydroorotate dehydrogenase (DHODH) protein is one of the therapeutic targets for SCLC [3].

DHODH, a flavin mononucleotide (FMN) flavoenzyme [4], is a key enzyme crucial for de novo pyrimidine synthesis located on the outer surface of the inner membrane of the mitochondrion [5]. The pyrimidine nucleotides in most organisms are centrally derived from the salvage pathways and the de novo synthesis. For malignant proliferating cells, the amount of pyrimidine nucleotides from the salvage pathway cannot sufficiently maintain their survival, and then to some extent, they are relied on in the de novo synthesis pathway [6]. Therefore, inhibiting the activity of DHODH means preventing the synthesis of biological macromolecules, such as DNA, RNA, and glycoprotein, and regulating the abnormal proliferation and metabolism of cells [7]. In recent years, DHODH has been proved to be a successful therapeutic drug target for a variety of diseases [8], such as cancer [3,9], viral infections [10], parasitic diseases [11], bacterial diseases [12], and autoimmune diseases [13].

Network-pharmacology-based analysis provides a novel approach to screen and discover drugs for multiple targets [14]. The effects of drugs that act on multiple targets not only combat complex systemic diseases [15] but also overcome challenges, such as emerging resistance or lack of efficacy due to single targeted drugs [16,17]. Several related target proteins, such as uridine 5’-monophosphate synthase (UMPS) and CAD protein, which are downstream and upstream of DHODH, respectively, on the pyrimidine biosynthesis pathway, should be focused on as well [18].

Artificial intelligence (AI) is a new technical science that researches and develops the intelligent theory, technology, and application for simulating and extending human intelligence [19,20,21]. Machine learning (ML) has shown its enormous potential to revolutionize drug discovery, and drug discovery is an extremely long, expensive, complex, and inefficient process that typically costs 2.6 billion USD and takes 12 years on average [19,20,21,22]. Quantitative structure–activity relationship (QSAR) models can predict the properties of molecules through mathematical models to describe the relationship between the structures of molecules and their biological activities [23]. Studies show that the application of machine learning on QSAR models, including support vector machines (SVMs), random forest (RF), and gradient boosting algorithm (GBR), has shown good performance in molecular property prediction. ML is also able to predict the effects of drugs on specific cancers [22]. Iorio et al. used ML algorithms to identify molecular features that predict drug response [24], and Christian et al. confirmed the presence of structural relationships that differentiate promiscuous and nonpromiscuous compounds via diagnostic machine learning [25].

Deep learning (DL), an area of machine learning where models extract latent message over many different layers and then learn the features represented in the data, has achieved remarkable success in drug discovery [26,27]. A deep neural network (DNN) is the foundation of a deep learning architecture, containing input, hidden, and output layers. Satoshi et al. developed novel unsupervised learning techniques to accurately predict the survival of patients with lung cancer using multiomics data, and first detected survival-associated subtypes in non-small cell lung cancer [28]. A drug-target interaction (DTI) model of a deep-learning-based algorithmic framework was developed by Wen et al. [29]. In the latest reviews, Shozu et al. proposed a novel model-agnostic method using deep learning techniques, the multiframe + cylinder method (MFCY), to improve the segmentation performance of the thoracic wall in fetal ultrasound videos [30].

For molecular graph structure, graph neural network (GNN) provides a method for directly extracting features from non-Euclidean structural data, and has achieved state-of-the-art performance on some molecular property prediction tasks [31]. First, the 2D molecular graph structures need to be transformed into adjacency matrices as graph representations of molecules, containing atoms and bonds information. The graph representations of molecule then go through a convolution operation to aggregate the neighboring atoms and bonds information. After passing through several fully connected neural layers, the final output is generated. It was reported that Xiong et al. [32] proposed a new GNN architecture that used a graph attention mechanism to learn from drug discovery datasets. Many other creative graph convolution neural networks were reported by Wen et al. [33], Jaechang et al. [34], and Wang et al. [35].

**Figure 1:**
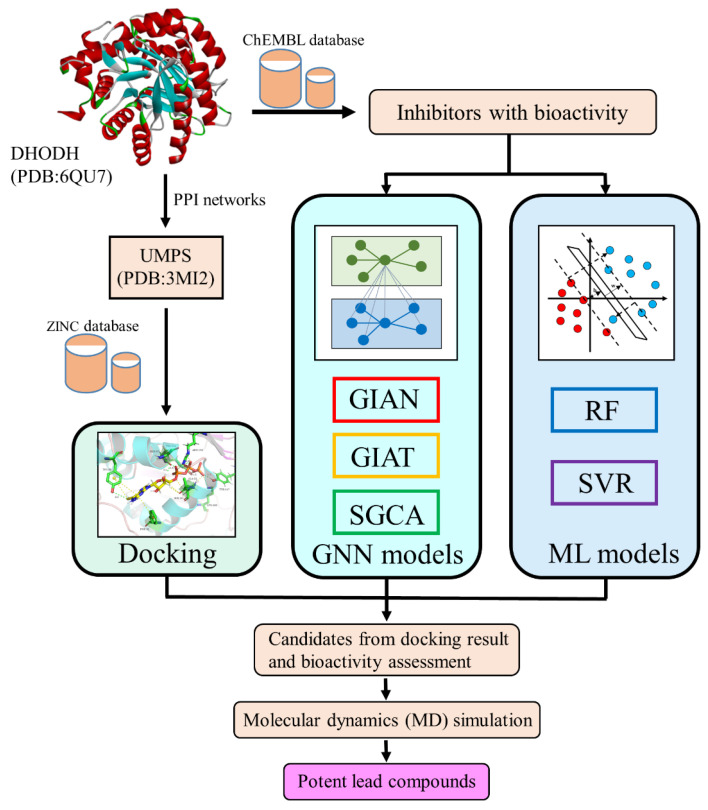
Flowchart of the experiment.

This article focuses on the DHODH protein and its related target, the UMPS protein, identified via network pharmacology in order to screen potential lead compounds from small molecular databases. Here, we introduce several composite architectures of GNN (GIAN, GIAT, and SGCA) and two machine learning methods (RF and SVR (support vector regression)) for the prediction of molecular biological activity. The docking results, GNN models, and machine learning could help us to identify the most promising lead compounds. The flowchart of our study is shown in Figure 1.

## 2. Materials and Methods

### 2.1. Network Pharmacology Analysis

To seek out the related targets of DHODH, the STRING database (version 11.0) [36] was used for network analysis of biological systems. Specific signal proteins were selected to screen molecules that could dock well with them. It provided biological signal pathways among the related proteins, and the protein–protein interaction (PPI) information was obtained from several databases of curated biological pathway knowledge, such as Kyoto Encyclopedia of Genes and Genomes (KEGG) and Reactome. The top 10 proteins with an interaction score of more than 0.400 were used to construct the PPI network, which was generated and visualized from the STRING database. The Kyoto Encyclopedia of Genes and Genomes (KEGG) database can provide the biological pathways data for functional enrichment analysis, and the multitarget can be found in this pathway.

### 2.2. Virtual Screening and Molecular Docking

The three-dimensional structures of two related target proteins, DHODH protein and UMPS protein, were acquired from the Protein Data Bank (PDB). The crystal structure of human DHODH (PDB: 6QU7) was complex with BAY 2402234 with cogent resolution (1.53 Å), and the crystal structure of human UMPS (PDB: 3L0K) was complex with 6-acetyl-UMP with 1.34 Å resolution. We used the BIOVIA Discovery Studio 2017 R2 Client software (created for Windows by Dassault Systèmes in Paris, France) in this part. Before carrying out the docking protocol, we used the “Remove Cell” protocol to remove all top-level cells while keeping their constituent contents intact. Then the “Prepare Protein” protocol was employed to prepare the protein, performing tasks such as inserting missing atoms in incomplete residues, removing crystal waters, and standardizing atom names. After that, the “Define and Edit Binding Site (Advanced) tools” protocol was used to calculate a binding site of DHODH from a selected ligand and display the receptor sphere; the selected ligand was a DHODH inhibitor named BAY 2402234 on the basis of Christian’s study [37]. A total of 10,152 molecule compounds obtained from the ZINC database [38] were utilized for docking so that we were able to find some of these approved drugs that could inhibit the DHODH protein and act on the UMPS protein at the same time.

The “Dock Ligands (LigandFit)” protocol followed three stages: docking, in situ ligand minimization, and scoring. During the docking process, the docked poses were used to be minimized with the Chemistry at Harvard Molecular Mechanics (CHARMM) and evaluated with a set of scoring functions, including LigScore1, LigScore2, PLP1, PLP2, Jain, PMF, PMF04, Ludi energy estimate 1, Ludi energy estimate 2, and Ludi energy estimate 3. Here, we also illustrated some other parameter settings. The DREIDING force field was chosen for calculating interaction energies for each ligand and the specified receptor. The numbers of Monte Carlo trials were set to “2500 120, 4 1200 300, 6 1500 350, 10 2000 500, 25 3000 750”, and the minimization algorithm was set to “Do not minimize”. Lastly, we set the parallel processing as “True” and the batch size as 25 in order to speed up the docking process.

### 2.3. Graph Neural Networks (GNNs)

GNNs are divided into two main domains: the spectral-based approaches and the spatial-based approaches. The spectral-based approaches compare the graph with signal processing and introduce a filter to realize the graph convolution. Graph convolution can be interpreted as the removal of noise from graph signals. The spatial-based approaches apply graph convolution through updating the representation for the central node by convolving the central node’s representation with its neighbors’ representations [39].

The spectral-based GNN is implemented in graph signal processing by mathematical operation. The graph convolution of the input signal x with a filter $g\in R^{n}$ is defined as:

(1) x*gG=F−1(F(x)⊙F(g))=U(UTx⊙UTg)

where ⊙ denotes the elementwise product, U is the matrix of eigenvectors ordered by eigenvalues, and *F*(x) is the graph Fourier transform to a signal x.

As for the spatial-based GNN, given that G = (V, E) denotes a graph with node feature vectors $x_{v}$ for v∈V, we suppose that each node and its adjacent nodes and even the edge indicate some latent message. The feature of the graph G is generated by learning from the representations of nodes $h_{v}$ for v∈V, or the entire graph structure. Spatial-based GNNs follow three phases: aggregate, combine, and readout. GNNs encompass an iterative procedure using a neighborhood aggregation strategy so that the representation of each node is iteratively updated, and then combine the feature of neighbor aggregation with the feature of the current node to update the feature of the current node. Readout is used for the classification task to transform all node features of the graph into graph features, but we used a fully connected network to carry out the regression task in our study. As per the existing GNN architectures [40], our GNN models are formulated as follows:

Aggregate:

(2) $$a_{v}^{\left(k\right)}={\mathrm{AGGREGATE}}^{\left(k\right)}\left(\left\{h_{u}^{\left(k-1\right)}\;|\;u\in N\left(v\right)\right\}\right)$$

Combine:

(3) $$h_{v}^{\left(k\right)}={\mathrm{COMBINE}}^{\left(k\right)}\left(h_{v}^{\left(k-1\right)},\;a_{v}^{\left(k\right)}\right)$$

where N(v) is a set of nodes adjacent to v and $h_{v}^{\left(k\right)}$ represents the features of node v at the k-th iteration.

Several GNN models and training strategies used in this study are introduced as the following, including two spectral-based GNNs (simple graph convolution (SGC) and autoregressive moving average (ARMA)) and two spatial-based GNNs (graph isomorphism network (GIN) and graph attention network (GAT)).

#### 2.3.1. Graph Isomorphism Network (GIN)

**Figure 2:**
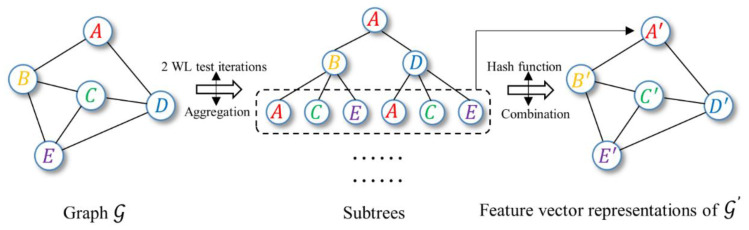
Overview of the graph isomorphism network (GIN) framework. The GIN model applies an injective aggregation function to capture the full multiset of node neighbors, which is similar to the Weisfeiler–Lehman test (WL test). Molecules with similar structures may have similar functional properties. The network has a strong ability to distinguish different graphs because each point has a unique representation.

Recently, Keyulu Xu et al. proposed an expressive model called graph isomorphism network (GIN) [40]. The GIN applies a novel neighborhood aggregation method rivaling the Weisfeiler–Lehman test (WL test) [40,41], which is the upper limit of GNN performance proved in the research (Figure 2). The GIN updates the node features at the *k*-th layer as:

(4) $$h_{v}^{\left(k\right)}={\mathrm{MLP}}^{\left(k\right)}\;\left(\left(1+\epsilon^{\left(k\right)}\right)\cdot h_{v}^{\left(k-1\right)}+\sum_{u\in N\left(v\right)}h_{u}^{\left(k-1\right)}\right)$$

where $h_{v}^{\left(k\right)}$ represents the features at node v for the k-th iteration, N(v) is a set of nodes adjacent to v, and ϵ is a learnable parameter. The multilayer perceptron (MLP) is applied to approximate any function so as to learn injective functions to achieve a performance similar to that of the WL test.

#### 2.3.2. ARMA Filter Network

Filippo Maria Bianchi introduced a GNN with the ARMA (autoregressive moving average) filters [42]. The advantage of the ARMA filters is that the resulting filters are localized in the node space and independent from the underlying graph structure instead of learning in the Fourier space. The ${\mathrm{ARMA}}_{K}$ layer uses a graph convolutional skip (GCS) layer to implement one recursive update, and the output of the ARMA convolutional layer is obtained by combining K parallel stacks of T GCS layers. The GCS layer and the output of the ARMA convolutional layer are defined respectively as:

(5) $${\bar{X}}^{\left(t+1\right)}=\sigma \left(\tilde{L}{\bar{X}}^{\left(t\right)}W^{\left(t\right)}+XV^{\left(t\right)}\right)\;\bar{X}=\frac{1}{K}\overset{K}{\underset{k=1}{\sum }}{\bar{X}}_{k}^{\left(T\right)}$$

where $W^{\left(t\right)}$ and $V^{\left(t\right)}$ are trainable parameters, X are the initial node features, and $\tilde{L}=I-L$ is the modified Laplacian matrix.

#### 2.3.3. Graph Attention Network (GAT)

**Figure 3:**
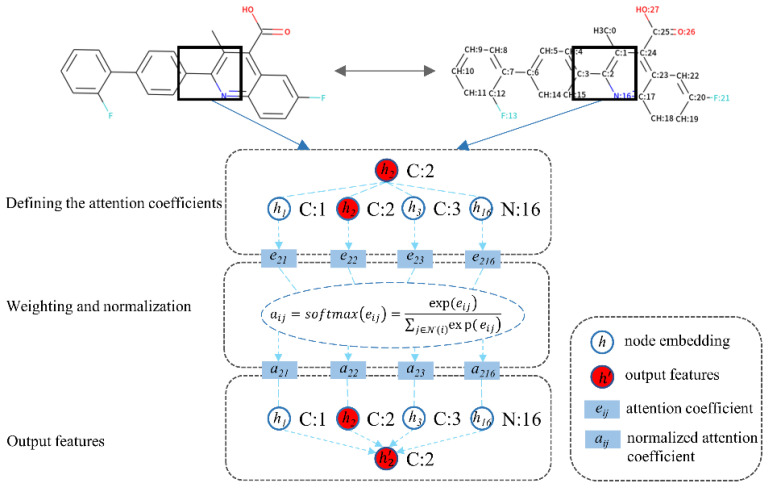
Illustration of the graph attention mechanism.

The core objective of the attention mechanism is to focus on the high-value information that is more critical to the current task from the neural network. At present, the attention mechanism has been widely used in various types of deep learning tasks, including but not limited to natural language processing, image recognition, and speech recognition. It is one of the most noteworthy core technologies in deep learning. Recently, Petar and Guillem et al. presented GATs that applied the attention mechanism to the GNN [43]. Such model defines the attention coefficients and learns the latent important features among a neighborhood of nodes (Figure 3). The process follows three steps: (1) defining the attention coefficients, (2) weighting and normalization, and (3) output features, as formulated below:

(1) Defining the attention coefficients:

(6) $$e_{ij}=LeakyReLu\left(\alpha \left[Wh_{i}\cdot Wh_{j}\right]\right)$$

(2) Weighting and normalization:

(7) $$a_{ij}=softmax\left(e_{ij}\right)=\frac{\exp\left(e_{ij}\right)}{\sum_{j\in N\left(i\right)}\exp\left(e_{ij}\right)}$$

(3) Output features:

(8) $$h_{i}^{’}=\sigma \left(\underset{j\in N\left(i\right)}{\sum }a_{ij}Wh_{j}\right)$$

where i is the target node and $h_{i}$ is the input features vector of node i, as is $h_{j}$ to the neighbor node j. Here, LeakyReLu and σ are both nonlinear activation functions that could consistently perform better. During the first step, the input features vectors of the target node and its neighbor node perform a linear transformation with a trainable weight matrix W, followed by a weight vector α and applying the LeakyReLu nonlinearity. $e_{ij}$ is the attention coefficient for each target–neighbor pair. During the second step, the coefficients are normalized using the softmax function to make them easier to calculate and easily comparable across overall nodes. $a_{ij}$ is a normalized attention coefficient that represents the importance of neighbor node j to target node i. During the third step, $h_{j}$ preforms a linear transformation with W, followed by a weighted sum and a nonlinear activation function σ, and finally, the output features of the target node $h_{i}$ are obtained.

#### 2.3.4. Simple Graph Convolution (SGC)

The SGC model was proposed to reduce the excess complexity of graph convolution networks [44]. Such model considers that the nonlinearities between the GCN layers are not the most critical, but rather the feature propagation of the local neighbors. Therefore, the linear model is obtained by repeatedly removing the nonlinear transformation function between each GCN layer and collapsing the resulting function into a single linear transformation, as formulated below:

(9) $$\hat{Y}=softmax\left(S^{K}XW^{1}W^{2}\ldots W^{K}\right)$$

(10) $$S={\tilde{D}}^{-\frac{1}{2}}\tilde{A}{\tilde{D}}^{-\frac{1}{2}}$$

where $\tilde{A}=A+I$, A is a symmetric adjacency matrix and $\tilde{D}$ is the degree matrix of $\tilde{A}$. S denotes the “normalized” adjacency matrix with added self-loops. The feature matrix X performs the repeated multiplication with the normalized adjacency matrix S and the learnable weights W for the k-th iteration. The resulting $\hat{Y}$ represents the class prediction for all nodes.

#### 2.3.5. Batch Normalization (BN)

As the depth of the network increases, the eigenvalue distribution of each layer will gradually get closer to the saturation interval of the output interval of the activation function, which may result in gradient vanish and slow convergence. Batch normalization (BN) was used to solve the internal covariate shift problem, avoid gradient vanish, and accelerate the convergence process [45]. By means of normalization, BN enhancing transforms the distribution of the input value of any neuron in each layer of a neural network back to the standard normal distribution with a mean value of 0 and a variance of 1 so that the eigenvalues will fall in the interval where the activation function is more sensitive to the input. In this way, a small change in the input can lead to a large change in the loss function, which can make the gradient bigger and avoid gradient vanish and overfitting to some extent. The batch normalization (BN) algorithm is given in Algorithm 1.
**Algorithm 1.** Batch Normalization**Input**: Values of x over a minibatch: $𝓑=\{x_{1\ldots m}\}$;
   Parameters to be learned: γ,β **Output**: $\left\{y_{i}={\mathrm{BN}}_{\gamma ,\beta }\left(x_{i}\right)\right\}$ $\mu_{𝓑}\leftarrow \frac{1}{m}\overset{m}{\underset{i=1}{\sum }}x_{i}\;//\mathrm{minibatch}\;\mathrm{mean}$ $\sigma_{𝓑}^{2}\leftarrow \frac{1}{m}\overset{m}{\underset{i=1}{\sum }}x_{i}\;//\mathrm{minibatch}\;\mathrm{variance}$ ${\hat{x}}_{i}\leftarrow \frac{x_{i}-\mu_{𝓑}}{\sqrt{\sigma_{𝓑+\epsilon }^{2}}}\;//\mathrm{normalize}$ $y_{i}\leftarrow \gamma {\hat{x}}_{i}+\gamma \equiv {\mathrm{BN}}_{\gamma ,\beta }\left(x_{i}\right)\;//\mathrm{scale}\;\mathrm{and}\;\mathrm{shift}$

### 2.4. Early Stopping

Early stopping terminates the training process to avoid overfitting and save training time when the performance of the model on the verification set starts to decline. It can also be considered as a regularization method similar to L1/L2 weight attenuation and discarding. In our training process, we set a maximum epoch of 1000 and a threshold of early stopping process of 60, which means that if the assessment criteria had not improved in 60 epochs, the training process was terminated early. However, the threshold setting of the early stopping process is empirical and could be variant on different training sets and tasks. We selected the appropriate threshold to make the model performance relatively better in our task.

### 2.5. Cross-Validation (CV) and Model Evaluation

Cross-validation (CV) was applied to assess the performance of the model, and model hyperparameters were optimized through internal 10-fold cross-validation. A single subsample was retained as data for the validation of the model, and the other nine samples were used for training. CV was repeated 10 times, and each subsample was validated once. Best hyperparameters were selected according to the average value of the 10-times validation root mean square error (RMSE; Equation (11)). We applied the RMSE as an evaluation criterion to measure the predictive results of each model. The RMSE is a frequently used measure of the differences between values (sample or population values) predicted by a model or an estimator and the values observed.

(11) $$RMSE\left(X,h\right)=\sqrt{\frac{1}{m}\overset{m}{\underset{i=1}{\sum }}{\left(h\left(x_{i}\right)-y_{i}\right)}^{2}}$$

### 2.6. Multi-GNN Models

GNNs show a variety of distinguishing characteristics and bring about an effect on account of their different aggregating or combining strategies. Individual models have different characteristics and focus on different domains, so we combined them to enhance the performance of the overall models.

**Figure 4:**
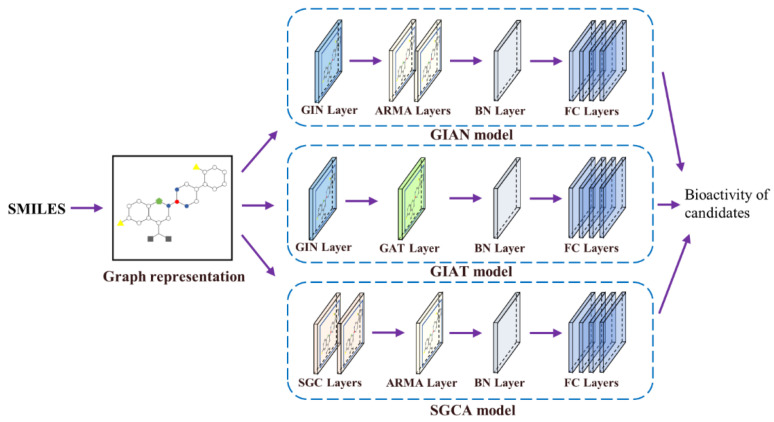
The overall architectures of the three multi-GNN models named GIAN, GIAT, and SGCA, respectively.

Here, we propose several multi-GNN models, including GIAN, GIAT, and SGCA, for our task through the combination and restructure of the different GNN models. We present the three overall architectures and provide details about some of the construction and modification processes in the following (Figure 4).

#### 2.6.1. Graph Representation of Molecules

Molecular graphs first needed to be transformed to a suitable input for GNN so that the model could availably extract a spatial feature for learning. Specifically, the graph structure of each molecule G was denoted with an edge connection matrix A and a node feature matrix X. The edge connection matrix $A\in R^{2\times n}$, where n denotes the number of edges in a molecule, represented the connection information between atoms in coordinate (COO) format. For example, $A_{i}$ for i∈n indicated that there was an edge connection between two nodes, which were represented as $A_{1i}$ and $A_{2i}$ in i-th column, respectively.

**Figure 5:**
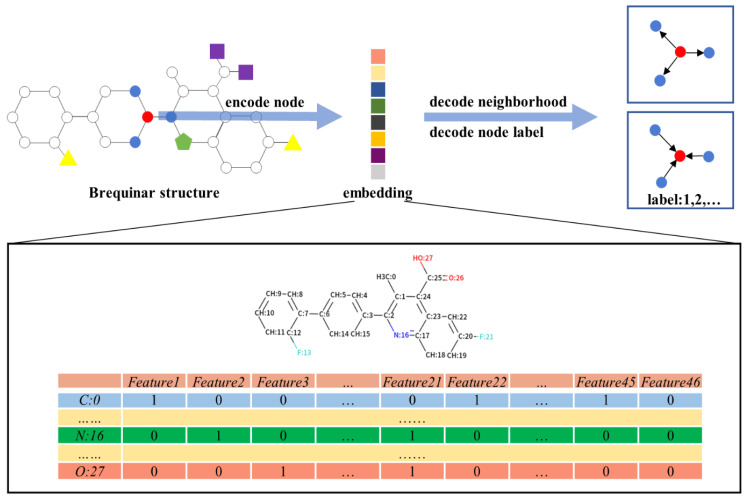
The construction of the molecules’ graph representation and initial feature matrix of the molecules. Atoms are coded to indicate the feature vector corresponding to atoms.

The node feature matrix $X\in R^{n\times m}$, where n denotes the number of nodes and m denotes the number of node features, represented the information of each node feature. The features include atom symbol, degree, hybridization, valence, formal charge, atom in ring of size, aromatic, and explicit hydrogen, which are introduced in Table 1. We used one-hot encoding for most of these features, except for aromatic, which was encoded as integers. After one-hot encoding, all categories of each feature were listed and sorted, and marked as either 0 or 1 by atomic category (Figure 5). For example, atom symbol was encoded as a vector of 12 bits, and degree was encoded as a vector of 7 bits. If the atom was a carbon atom and the number of its covalent bonds was 2, the first site of the atom symbol vector and the third site of the degree vector were marked as 1; the other sites in both vectors were marked as 0.

#### 2.6.2. GIAN Model

The model was constructed by applying GIN and ARMA filters. Spatial-based approaches perform graph convolution locally at each node, making it easy to share weights among different locations and structures, while spectral-based approaches use the graph signal filtering method to convolve. Spatial-based GIN, which is one of the most powerful models in recent years, was chosen as the base model, and given that GIN’s strategy focuses on global structure, we chose spectral-based ARMA filters to implement local attention. GIAN is a combination model of a spatial-based approach and a spectral-based approach.

#### 2.6.3. GIAT Model

GIN can theoretically distinguish between each of the different graph structures, so it focuses on the entire molecular structures. GAT borrows the attention mechanism to achieve better neighbor aggregation and is able to update the target node’s representation by learning from its neighbors and local environment, which means paying more attention to local features. The combination of a global approach and a local approach is able to improve the performance of the prediction model. GIAT is a combination model of two spatial-based approaches.

#### 2.6.4. SGCA Model

SGC, reducing the complexity of the graph convolution network by removing the weight matrix between the nonlinear transformation and the compressing convolutional layer, was also an available option. SGCA is a combination model of two spectral-based approaches.

In all of our models, each molecule and its atomic features were first extracted with RDkit and encoded in a one-hot fashion. Then, to focus on the most relevant information on its neighbors and gain the final latent features embedded in each molecule, we used different GNN models described previously and integrated them. Moreover, the batch normalization (BN) layer connected these different GNN layers to accelerate the training speed, avoid the vanishing gradient problem, and improve the generalization ability of the network. Finally, the last embedding containing the structural information about the molecular graph was used to predict the biological activity of each molecule through four fully connected layers. Specifically, the input unit and output unit of the first layer were set to 100 and 200, respectively. The output units of the second and third layers were set to 300 and 200, respectively. For our regression tasks, the last layer had only one unit and was not activated.

#### 2.6.5. Datasets of DHODH Inhibitors

The SMILES (simplified molecular input line entry specification) information of hundreds of compounds with an inhibited effect on DHODH was acquired from the ChEMBL database (Table S1) [46]. We filtered the raw data by removing some data without a 2D molecular structure or biological activity information. In order to enhance the generalization ability and reliability of the model, after filtering, 20% of the remaining 532 compounds were randomly set to a test set, and the rest to a training set. In addition, we counted the biological activity value distributions of DHODH inhibitors and the distributions of the training set and testing set.

#### 2.6.6. Training Protocol

PyTorch Geometric [47], a library for deep learning on irregularly structured input data, such as graphs, was implemented to construct the graph representation of molecules and train our models using the Adam optimizer for gradient descent optimization. Modeling experiments were carried out using a machine with an Intel^®^ Core^TM^ i7-9700K at 3.60 GHz × 8 CPU, 15.6 GiB of RAM, and an NVIDIA GeForce RTX 2060 SUPER/PCle/SSE2 graphics card. Our code and dataset are available on GitHub.

### 2.7. Construction of 2D-QSAR Model

A total of 532 compounds were also applied to build 2D-QSAR models. The “*Calculate Molecular Properties*” protocol of the DS software was used to calculate the 204 properties of the DHODH inhibitors for creating a 2D-QSAR model. All IC_50_ values and pIC_50_ values of the 532 compounds (Table S1) retrieved from the ChEMBL database represent the bioactivities of the compounds. The 204 calculated properties of the compounds were treated as feature vectors, pIC50 as training label, and the precision of the predicted bioactivities from different models as an assessment of each model. Then we employed the Pearson correlation coefficient matrix to examine the correlation and orthogonality between each feature. Additionally, principal component analysis (PCA) and Lasso feature selection were used for data preprocessing by following this step: Feature selection and standardization of datasets. Features with a variance higher than 0.01 were selected and standardized to a mean of 0 and a variance of 1. The Lasso feature selection was used to further filter the features. Finally, the predicted bioactivities trained by the QSAR models were the evaluation of ZINC database candidates. The Pearson correlation coefficient was calculated with Equation (12):

(12) $$r=\frac{N\sum^{\;}x_{i}y_{i}-\sum^{\;}x_{i}\sum^{\;}y_{i}}{\sqrt{N\sum^{\;}x_{i}^{2}-{\left(\sum^{\;}x_{i}\right)}^{2}}\sqrt{N\sum^{\;}y_{i}^{2}-{\left(\sum^{\;}y_{i}\right)}^{2}}}$$

### 2.8. Molecular Dynamics (MD) Simulation

The protein–ligand complexes of all candidates were used for MD simulation in 300 ns with the Gromacs 2020 software. The candidates were processed by SwissParam [48] to receive the topology and parameters file. In the MD process, the leapfrog algorithm was utilized to integrate Newton’s laws, and the energy was optimized by the steepest descent minimization algorithm in 5000 steps. The MD simulation system employed a CHARMM27 force field and a periodic cubic box with a margin of 1.2 nm. In addition, the explicit solvent water model TIP3P and 0.145 M Na^+^ and Cl^−^ ions were added to the system to mimic the physiological conditions. The canonical ensemble (NVT) balanced the system with position constraints by keeping the system volume and temperature constant, and then the isothermal–isobaric ensemble (NPT) modified the density of the system by keeping the system temperature and pressure constant. The NVT and the NPT were both processed for a total of 10 ns. All bonds were constrained with the Lincs algorithm, and temperature coupling was on with a setting of 310 K for each group. The Verlet scheme was applied for neighbor search cutoff, and the pressure coupling was on with Parrinello–Rahman coupling. The MD simulation was performed in 150,000,000 steps with 2 fs for each step. Through MD analysis, we could acquire the calculated results, including the molecular framework, root-mean-square deviation (RMSD), total energy, root-mean-square fluctuation (RMSF), radius of gyration (gyrate), solvent accessible surface area (SASA), and mean square displacement (MSD).

## 3. Results and Discussion

### 3.1. PPI Network Analysis for Potential Target Proteins Related to DHODH

**Figure 6:**
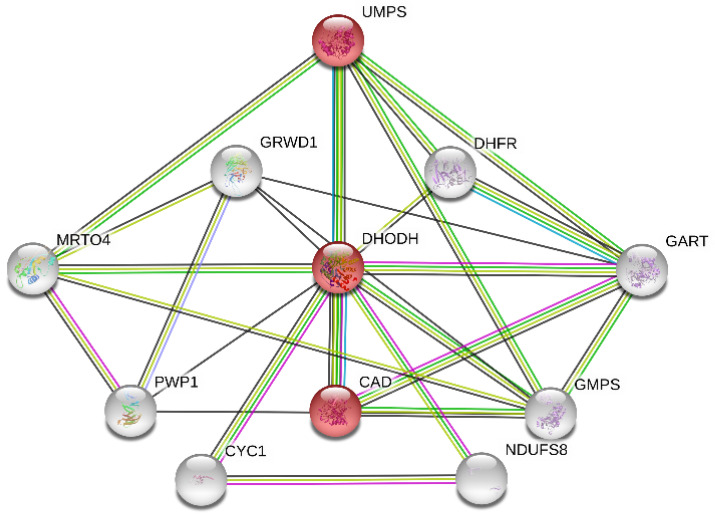
PPI network.

**Figure 7:**
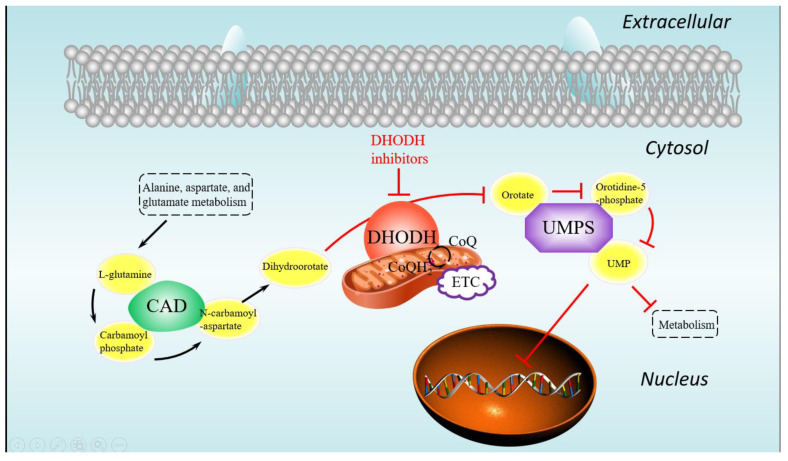
The pyrimidine biosynthesis in the pyrimidine metabolism pathway.

To identify the potential target proteins that could work together with the DHODH protein, the PPI network (Figure 6) was constructed through STRING v11.0, where the functional enrichment analysis of this network was performed. The average local clustering coefficient of the PPI network is 0.816. Based on the analysis above and the action mechanism of the DHODH protein, the pyrimidine metabolism pathway (colored in red; hsa00240) from the KEGG database with false discovery rate (FDR) values of only $1.8\times 10^{-4}$ was worthy of attention. The pyrimidine biosynthesis (Figure 7) in the pyrimidine metabolism pathway containing mainly three proteins, DHODH, CAD, and UMPS, plays a significant role in the de novo pyrimidine synthesis. As mentioned above, inhibiting the DHODH protein or the pyrimidine biosynthesis is able to regulate the abnormal cell proliferation and metabolism, so it achieves the anticancer effect. In addition, the combined score between DHODH and UMPS was 0.99, which indicates that drugs acting on them at the same time may have a greater effect. Therefore, two proteins, DHODH and UMPS, which should be inhibited, were focused on as potential targets in this research.

### 3.2. Docking Results

The whole molecular docking protocols of the DHODH protein and the UMPS protein were applied with the same parameters and accomplished in 9.0 h and 11.5 h, respectively. It needs to be noted that for the UMPS protein, the inhibited site for docking was defined in the crystal structure based on the related research. As for the DHODH protein, there were 20,793 poses docked, and 5586 poses filtered or failed to dock. One the other hand, for the UMPS protein, there were 43,317 poses docked, and 1289 poses filtered or failed to dock. 

**Figure 8:**
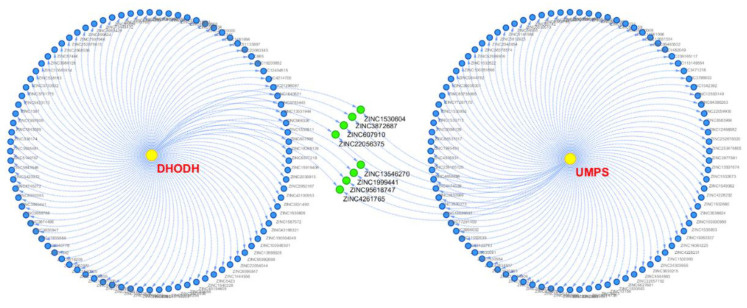
The compound–target interaction network.

The compound–target interaction network displayed the interaction of the top 150 candidates of the corresponding proteins, where the potential multitarget compounds were focused on (Figure 8). The blue points denote the different compounds of the corresponding proteins, the yellow points denote the target proteins, and the green points expressed specially in the middle denote the potential multitarget compounds. The results show that several molecules were associated with DHODH and also relevant to UMPS. Therefore, based on the interaction between two proteins and the docking results, we believe that it was credible to focus our drug selection on them.

### 3.3. Multi-GNN Models

**Figure 9:**
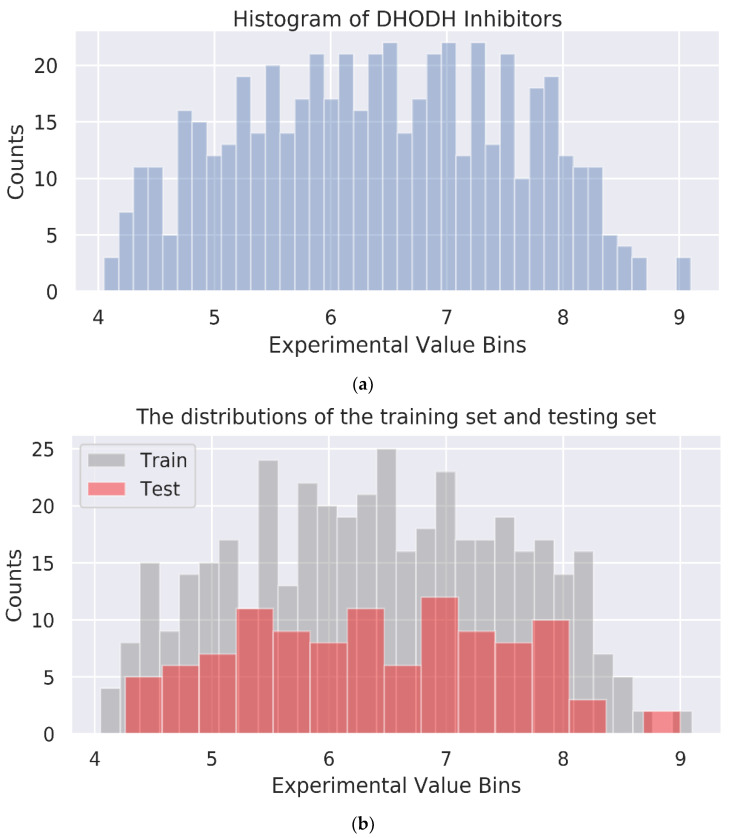
Dataset used for building prediction models. (**a**) The histogram of DHODH inhibitors. (**b**) The distributions of the training set and testing set.

Three multi-GNN models were constructed based on 532 compounds containing the SMILES information and their biological activity values. The dataset was randomly split with the ratio of train/test at 8:2. The histogram of DHODH inhibitors and the distributions of the training set and testing set are shown in Figure 9.

We trained our model for a maximum of 1000 epochs or until convergence (i.e., none of the metrics improved after 60 epochs) with early stopping operation. Then, the Adam optimizer with a learning rate of 0.001 was applied in the training process. In addition, dropout is one of the most effective and commonly used regularization methods for neural networks. The dropout technique was also applied in the GNN layers (with a rate of 0.35 for the ARMA layers and 0.06 for the GAT layers) to reduce overfitting. RMSE and R-squared were applied to evaluate the model accuracy. R-squared is a statistic used in the context of statistical models and provides a measure of how well observed outcomes are replicated by the model based on the proportion of the total variation of outcomes explained by the model. The better the linear regression fits the data in comparison with the simple average, the closer the value of R-squared is to 1. A dataset has n values marked $y_{i}$ for i∈n, each associated with a predicted value $f_{i}$ for i∈n. R-squared was defined as formulated below:

(13) $$R^{2}=1-\frac{SS_{res}}{SS_{tot}}=1-\frac{\sum_{i}{\left(y_{i}-f_{i}\right)}^{2}}{\sum_{i}{\left(y_{i}-\frac{1}{n}\sum_{i=1}^{n}y_{i}\right)}^{2}}$$

**Figure 10:**
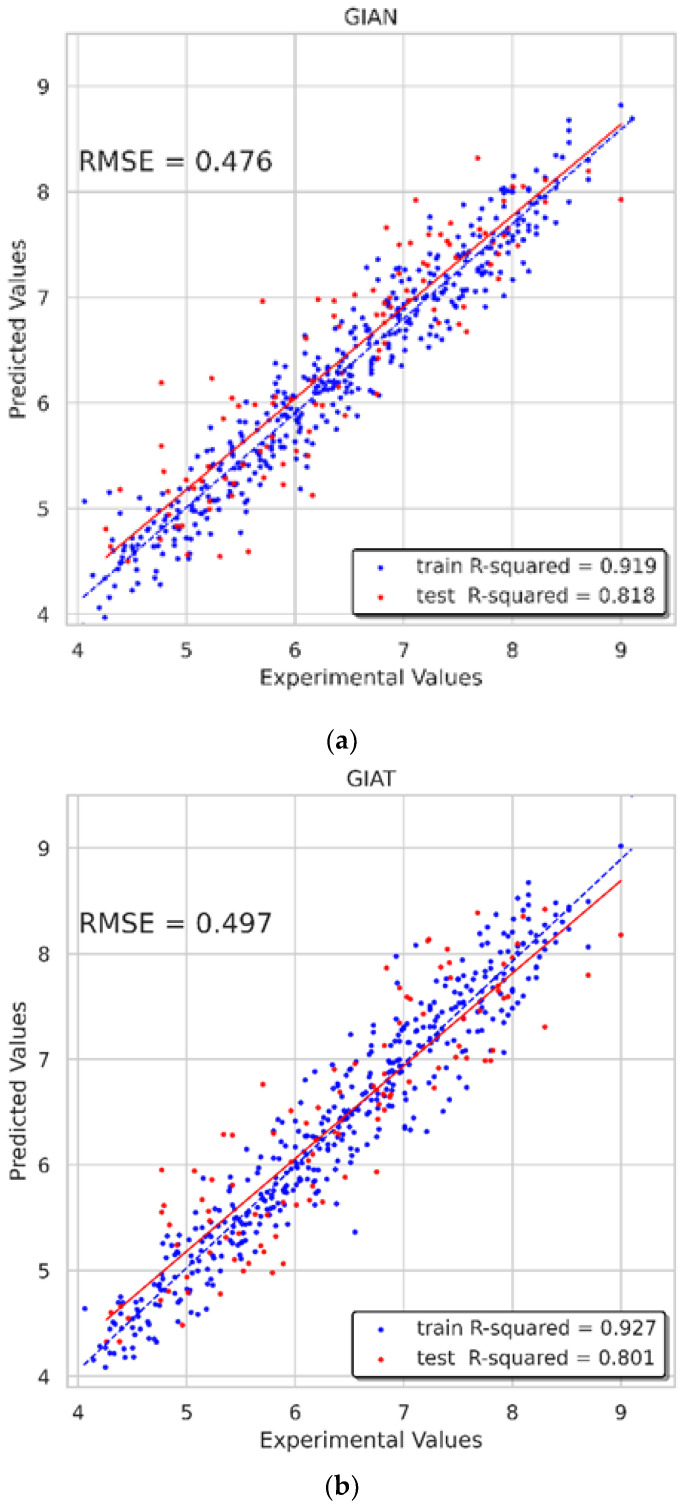

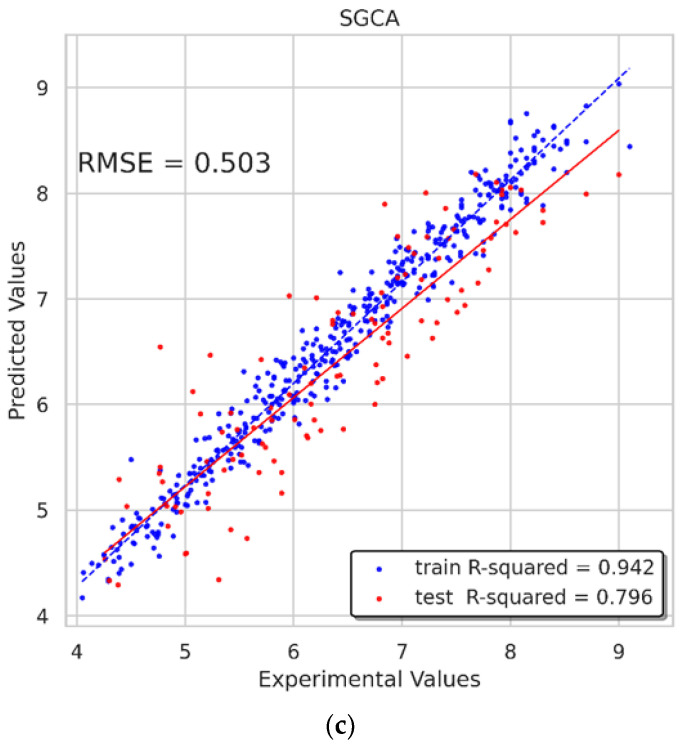
Results of three multi-GNN models. (**a**) Result of GIAN model; (**b**) Result of GIAT model; (**c**) Result of SGCA model.

We got satisfactory results, shown in Figure 10. The RMSE of the GIAN model on test sets was 0.476, and the R-squared on the training and test sets reached 0.919 and 0.818, respectively. As for the GIAT model, the RMSE on the test sets was 0.497, and the R-squared on the training and test sets reached 0.927 and 0.801, respectively. The R-squareds of the SGCA model on the training and test sets were 0.942 and 0.796, respectively, and the RMSE was 0.503. The performances of these models were satisfying and inspiring.

We also trained several single-GNN models as comparisons of multi-GNNs models, with specific results shown in Table 2.

### 3.4. RF Model and SVR Model

**Figure 11:**
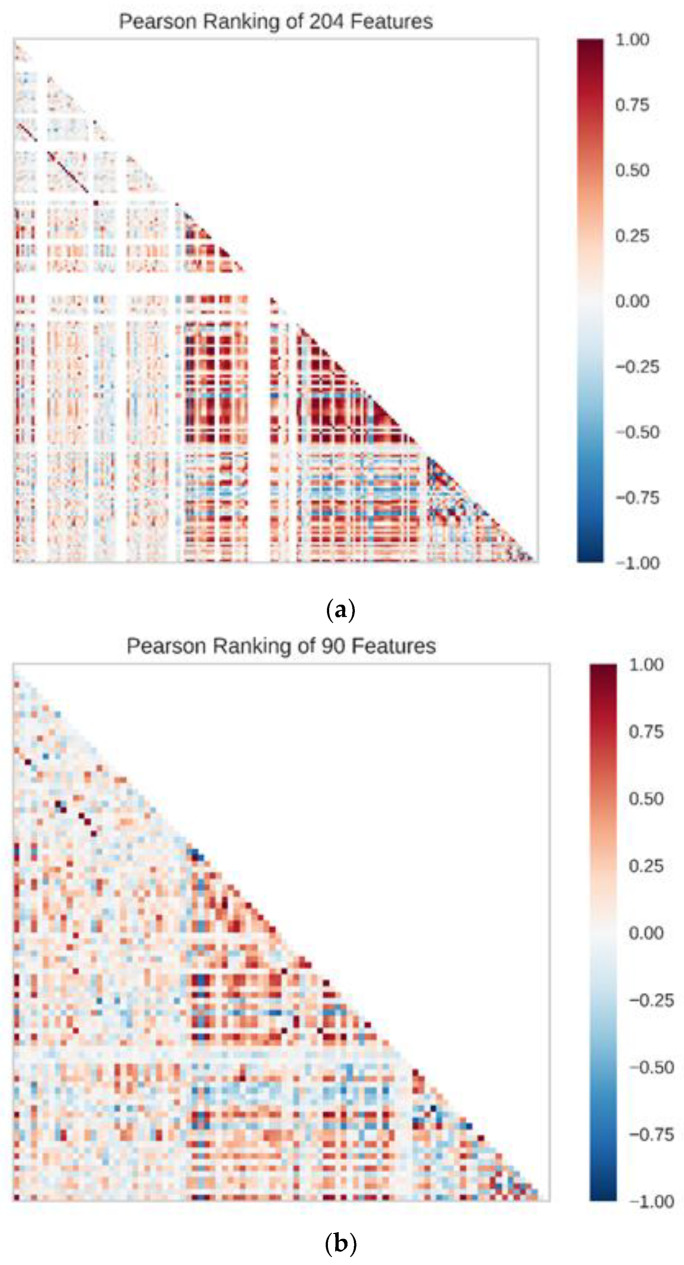
(**a**) Result of the Pearson correlation coefficient of 204 features; (**b**) 90 features with small correlation coefficients.

**Figure 12:**
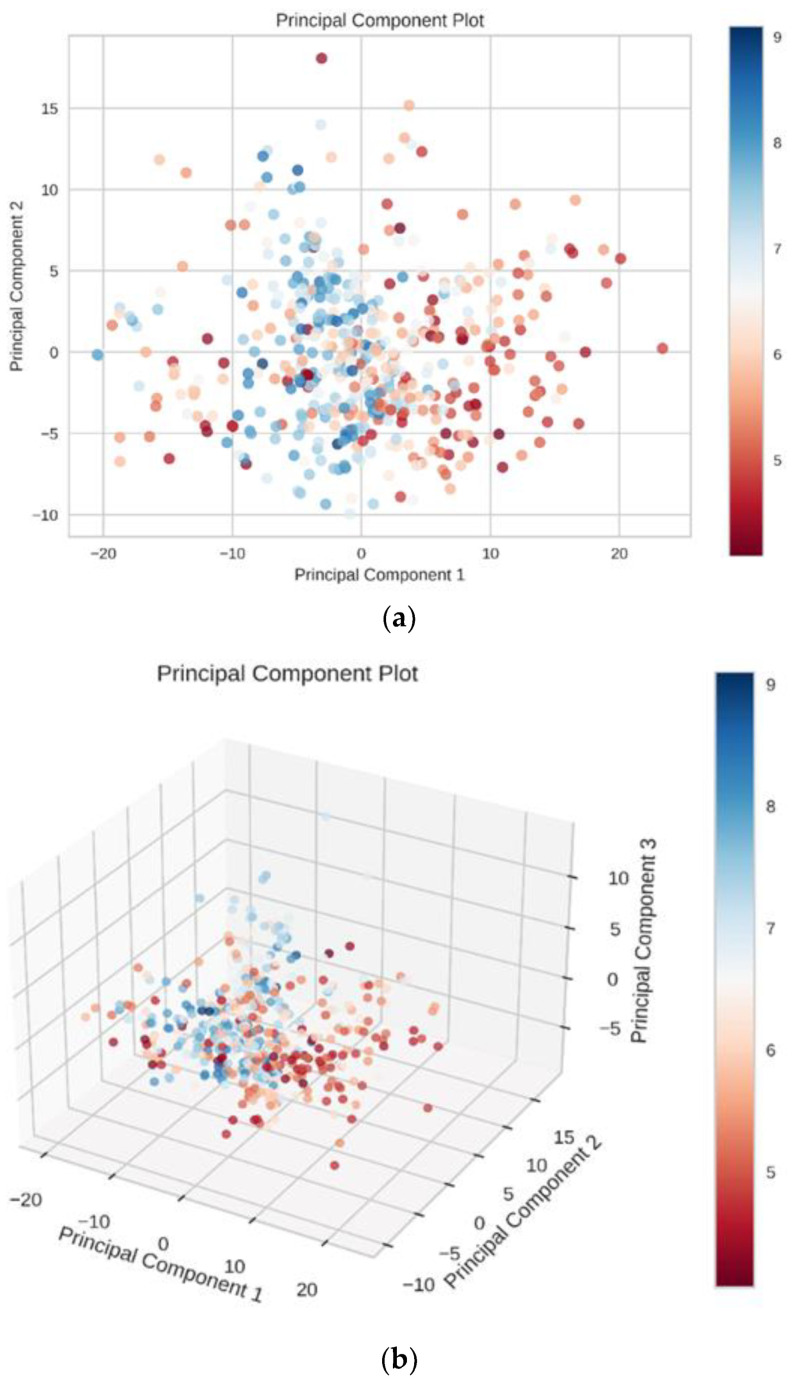
The results of the 2D principal component analysis (**a**) and the 3D principal component analysis (**b**).

The Pearson correlation coefficients of 204 features were first to be calculated to figure out the correlation between features before building the predicting model. Additionally, the Pearson rankings of 204 features indicated that some of the features had a high correlation, with correlation coefficients greater than 0.4 (Figure 11a). We used principal component analysis (PCA) to reduce the dimensionality of our dataset while maintaining the features of the dataset that contribute the most to each other by retaining the lower-order principal components and ignoring the higher-order principal components. The 2D and 3D principal component plots displayed the result of dimensionality reduction (Figure 12). In these two principal component plots, the color of each point on the plots was based on the value of the target. In data preprocessing, 204 features with a variance higher than 0.01 were filtered out and 160 features were chosen. Then, the selected features were standardized to a mean of 0 and a variance of 1. Lasso feature selection was performed to further filter 160 features, and finally, 90 features with small correlation coefficients and good orthogonality were obtained (Figure 11b). In this process, we found that the smaller number of features would not lead to a better predicting effect. We believe the reason is that our dataset was relatively large, and the number of features was small, which may easily lead to indistinguishable features and data overlap. The same preprocessing step was used for the RF model and SVR model. Finally, the known experimental activity values and the predicted activity values of our dataset were used for calculating the correlation coefficient ($R^{2}$) to validate the prediction accuracy.

**Figure 13:**
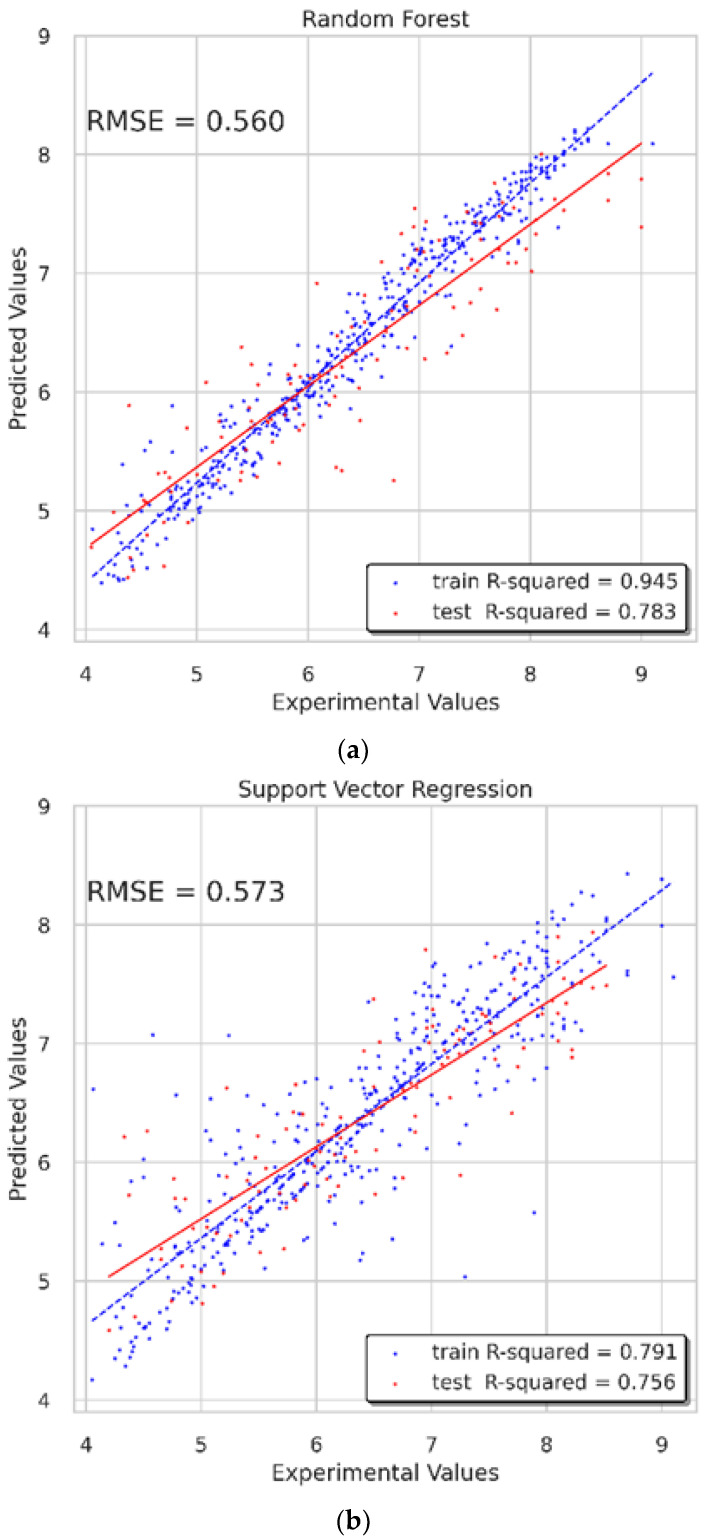
Results of traditional machine learning models. (**a**) Result of RF model; (**b**) Result of SVR model.

With regard to the RF model, the main parameter was *n_estimators*, which was the number of trees in the forest, which was set as 225, and the size of the random subsets of features to consider when splitting a node, *max_features*, was set as “None,” which meant that all features, instead of a random subset, were always considered. In addition, the parameter *random_state* was set as 2, and *min_samples_split* was set as 2. On the training set, the RMSE was 0.266 and the $R^{2}$ value was 0.945. On the test set, the RMSE was 0.56 and the $R^{2}$ value was 0.783. Overall, the performance of this model was good, and the predicting results were credible (Figure 13a).

For the SVR model, the parameter *kernel* standing for the kernel type used in the algorithm was set as “rbf.” The other three parameters, *tol, epsilon*, and *random_state*, were set as 0.0017, 0.1, and 55, respectively. The validated results display that the RMSE of the training set was 0.505, and that of the test set was 0.573. The $R^{2}$ value was 0.791 on the training set, and 0.756 on the test set (Figure 13b).

### 3.5. Discussion of Multi-GNNs

Different from machine learning algorithms, the 2D-QSAR model needed to be constructed to obtain the properties of candidates as input. GNNs captured the internal information directly from the graph structure, which means the SMILES of the molecules are transformed to a graph representation of molecules. The results show that the multi-GNN models had a better modeling effect and higher precision and lower RMSE value in the testing process than the two machine learning algorithms, RF and SVR models. During the experiment, we found that for large datasets, the 2D-QSAR model and the hyperparameter setting of the machine learning algorithms took a long time to construct and search. Moreover, when only a few properties were selected through PCA and Lasso feature selection, the model fitting results would be poor. 

On the other hand, the construction of the graph representation of molecules was more flexible and diverse. Specifically, we were able to not only select flexibly the features of nodes and edges but also use a variety of strategies for aggregation and combination. Related studies have also reported that a lot of novel GNN architectures have proposed and achieved state-of-the-art performance in several bioinformatics datasets, such as PROTEIN [49], NCI-1 [50], QM9 [51], and PPI [52].

In our study, we applied several existing GNNs and some deep learning strategies to combine and construct regression models for our dataset. The concept of multi-GNNs may put forward an original train of thought to construct the GNN architectures. The performance of the multi-GNNs was reliable and was better than that of the machine learning and single GNN (Table 2). The strategy of combining a global approach and a local approach achieved a model performance improvement.

However, how to select and combine different GNNs and even the hyperparameter setting was based on technique, experience, and task requirements. Although many existing GNNs could achieve state-of-the-art performance on public datasets, they may lack performance in some practice, like the dataset we built for predicting the biological activities of DHODH inhibitor candidates. One the other hand, according to our learning curve, the validation curve did not plateau at the maximum training set size used, and it still had potential to decrease and converge toward the training curve. Therefore, adding more training instances was very likely to improve our current models. Therefore, what we suppose to further study is how to apply the GNNs in drug discovery better.

In conclusion, the model generated by multi-GNNs and machine learning algorithms provided a credible referenced indicator for drug screening in our study. Additionally, multi-GNN still has a lot of potential to be explored and exploited.

### 3.6. Selection of ZINC Candidates

**Figure 14:**
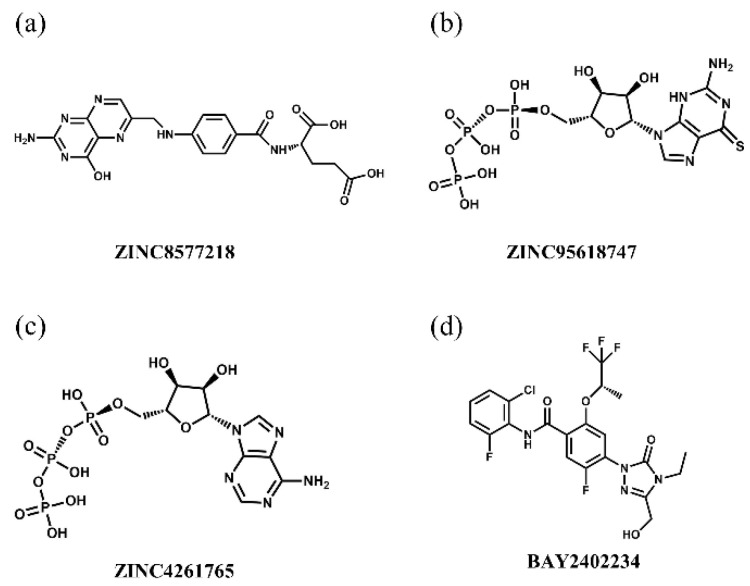
Chemical scaffolds of (**a**) ZINC8577218, (**b**) ZINC95618747, (**c**) ZINC4261765, and (**d**) BAY 2402234.

According to the docking score and the bioactivity prediction results generated from multi-GNN models and 2D-QSAR models (Table 3), the ZINC candidates were screened out convincingly and reasonably. The compound–target interaction network (Figure 8) showed that several ZINC candidates, including ZINC4261765 and ZINC95618747, had the potential to act on multiple proteins. Considering the docking score, all the five methodologies, and the drug–target interaction, we voted for these factors, and the voting score results are displayed in Table 4. ZINC8577218, ZINC4261765, and ZINC95618747 had great performance with high vote scores among the top 10 candidates and thus were determined as candidates; the last two target multiple proteins. Their chemical scaffolds are displayed in Figure 14.

**Figure 15:**
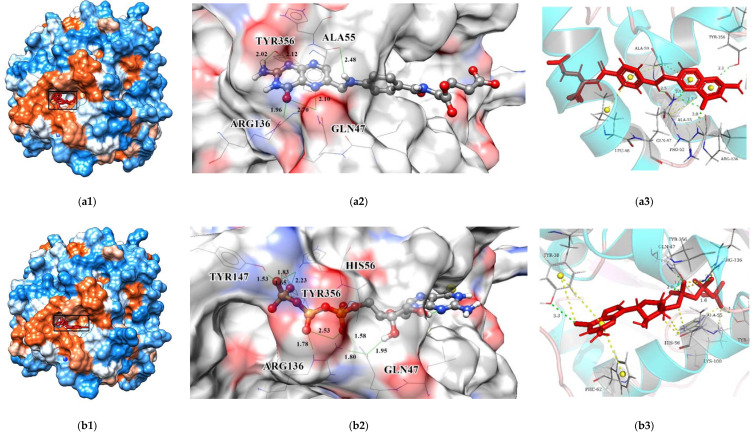

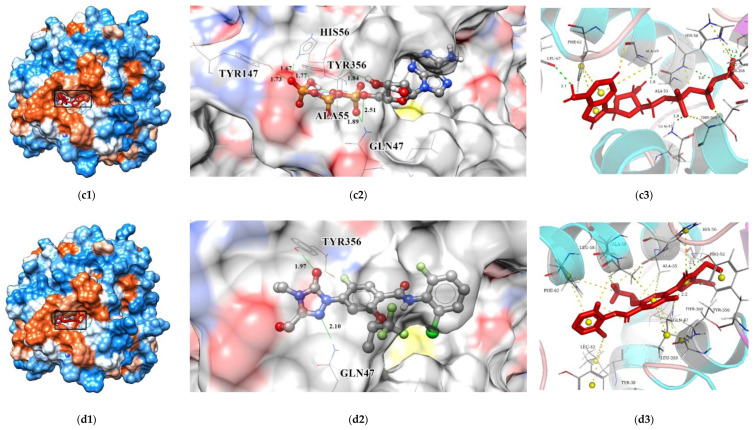
(**a**) Three-dimensional horizon of molecular docking result of ZINC8577218; (**b**) Three-dimensional horizon of molecular docking result of ZINC95618747; (**c**) Three-dimensional horizon of molecular docking result of ZINC4261765; (**d**) Three-dimensional horizon of molecular docking result of BAY 2402234. Each of these hydrogen bond lines (green) and their lengths (Å) are displayed. The green lines stand for H-bonds.

The interactions between key residues and candidates within macromolecules are presented in 2D and 3D horizons (Figure S1 and Figure 15). Analysis of the hydrogen bonding status, a significant reference of binding capacity, showed that these three chosen candidates and a control set have H-bonding interactions with TYR356 and GLN47 of DHODH in common. Moreover, both ZINC8577218 and ZINC4261765 form an H-bond with AL55; both ZINC95618747 and ZINC4261765 form an H-bond with TYR147. ZINC8577218 and ZINC42617 form a hydrogen bond with ARG136 and LEU47, respectively. Furthermore, besides a hydrogen bond, each ligand engages with different residues through diverse binding interactions, including van der Waals, pi interaction, and salt bridge, which might enhance the binding affinity with DHODH. Each of these hydrogen bond lines (green) and their lengths (Å) are displayed in the 3D docking interactions diagram. Therefore, three candidates were used for further MD simulation.

### 3.7. Molecular Dynamics Analysis

The protein–ligand systems of the top 3 candidates were validated through 300 ns MD simulations. It was exciting that two proteins remained combining well with all these candidate ligands at the end of the MD simulation, and the following analyses provided sufficient validations. 

**Figure 16:**
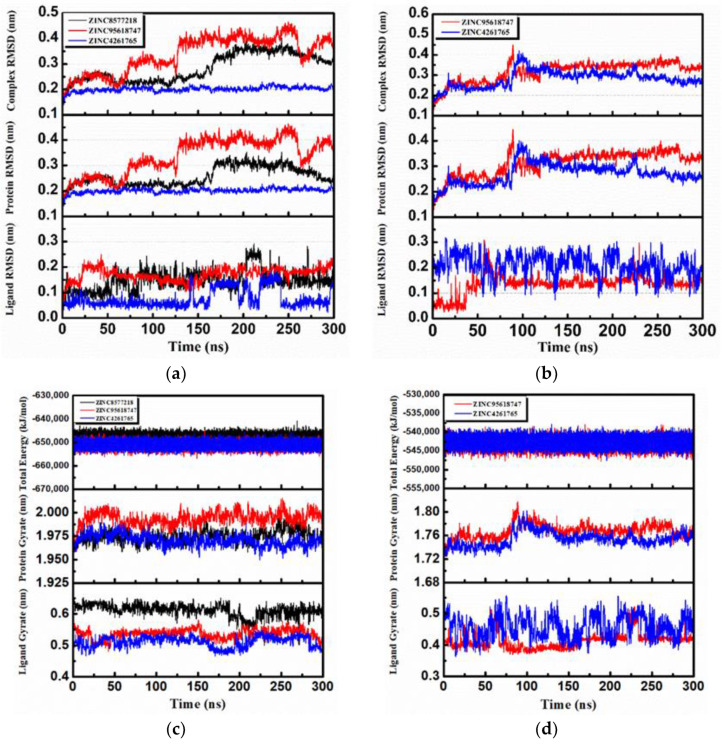
Root-mean-square deviation (RMSD), total energy, and gyrate analysis of each receptor–ligand complex in molecular dynamics simulation. (**a**) RMSD of the complex, protein, and ligand of DHODH complexes. (**b**) RMSD of the complex, protein, and ligand of UMPS complexes. (**c**) Total energy and gyrate of protein and ligand of DHODH complexes. (**d**) Total energy and gyrate of protein and ligand of UMPS complexes.

The RMSD changes (including complexes, proteins, and ligands) were calculated and then analyzed to not only reveal the position change between the conformation and the initial conformation of the protein during the simulation, but also determine whether the simulation was stable. It was obvious that the complex RMSD of DHODH–ZINC4261765 (Figure 16a, blue) was stable during the entire MD period. The complex RMSDs of the other two candidates were bumped up in 125 ns (Figure 16a, black) and 175 ns (Figure 16a, red), respectively, which suggests that the interactions changed in the conformation. They both tended to stabilize after the fluctuations. The trend of the protein RMSD value was similar to that of the complex RMSD value. Moreover, in general, the ligand RMSD tended toward a stable range over time, although it rose at some time. As for the UMPS protein, both ZINC95618747 (Figure 16b, red) and ZINC4261765 (Figure 16b, blue) showed good interactions with the receptor. The Ligand RMSD was in a dynamic equilibrium as well. The RMSD analysis indicated preliminarily the reliable stability of the protein structures with the binding candidates.

Further analyses of the total energy (Figure 16c,d) and the radius of gyration (gyrate; Figure 16c,d) provided the complexes state information during the MD process. We found that the total energies of all the systems were stable and the total energy values of the same protein were similar. The radius of gyration can be used to characterize the compactness of a protein structure and represent the change of peptide chain looseness in the simulation process. In general, the bulkier the protein, the smaller the gyrate value and the more difficult it would be for the ligand to “escape.” Both DHODH and UMPS had the relatively highest protein gyrate value when combined with ZINC95618747 (Figure 16c, red), indicating that their protein structures were relatively loose and less stable. The other gyrate value curves (including protein and ligand) were relatively flat, which was consistent with the RMSD analysis.

**Figure 17:**
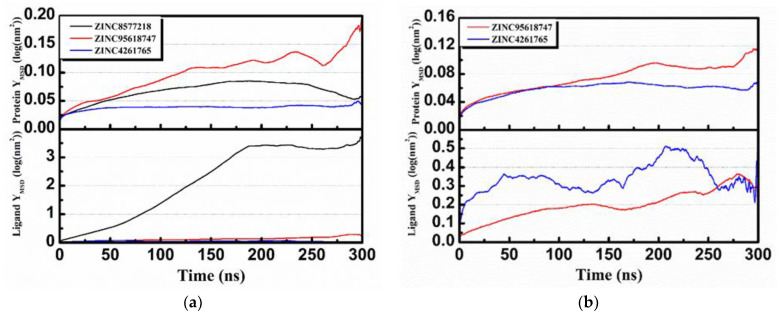

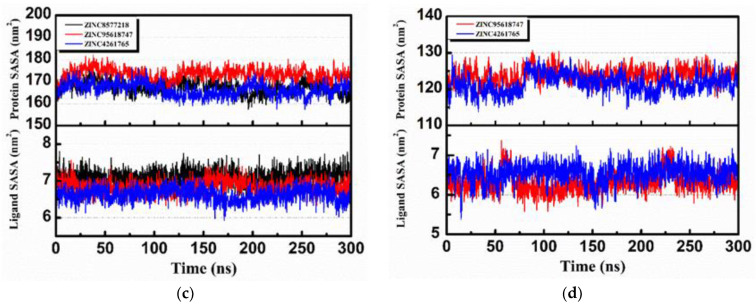
Mean square displacement (MSD) and solvent accessible surface area (SASA) analysis of each receptor–ligand complex in molecular dynamics simulation. (**a**) MSD of the protein and ligand of DHODH complexes. (**b**) MSD of the protein and ligand of UMPS complexes. (**c**) SASA of the protein and ligand of DHODH complexes. (**d**) SASA of the protein and ligand of UMPS complexes.

Mean square displacement (MSD) and solvent accessible surface area (SASA) analysis of each protein and ligand are shown in Figure 17. MSD is a measure of the position deviation of the particle over time relative to the reference position, following the logarithmic transformation function $Y_{\mathrm{MSD}}=2+\log_{10}\mathrm{MSD}\;\left(nm^{2}\right)$. The analysis illustrates that all protein MSD values were low (less than 0.2), suggesting that all of the simulation systems were stable during MD. Except for ZINC8577218 (Figure 17a, black), the low ligand MSD values also showed good affinity of the ligands staying with protein complexes. This might be caused by the poor binding capacity of ZINC8577218 with some residues of protein so that the ligand would have a relatively higher displacement. SASA analysis provided information about the hydrophilic and hydrophobic abilities of the system. The SASA values of both proteins and ligands changed little during the MD period, and there were no significant differences in hydrophilicity and hydrophobicity.

**Figure 18:**
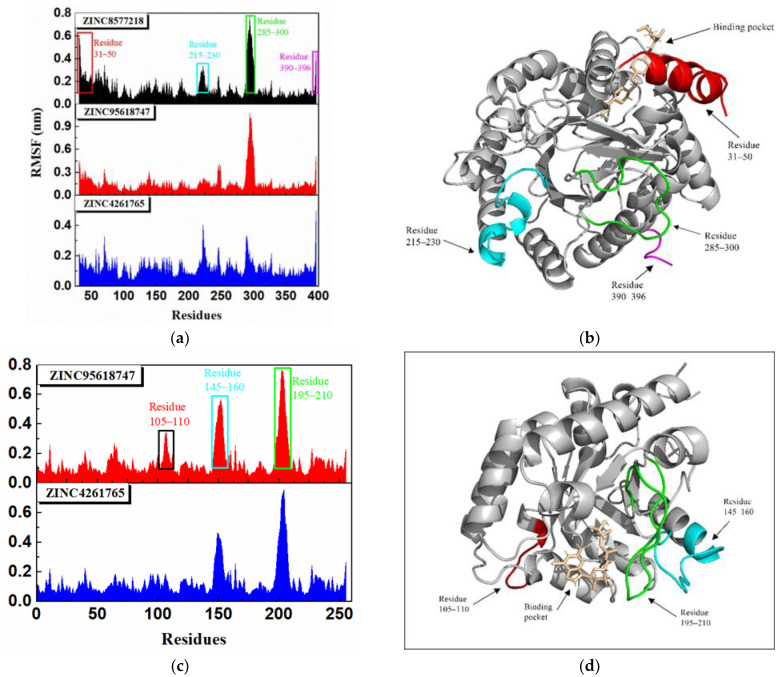
Root-mean-square fluctuation (RMSF) analysis of each receptor–ligand complex in molecular dynamics simulation. (**a**) RMSF of DHODH complexes. (**b**) The unstable region of the DHODH protein combined with ZINC8577218. (**c**) RMSF of UMPS complexes. (**d**) The unstable region of the UMPS protein combined with ZINC95618747.

The root-mean-square fluctuation (RMSF) shown in Figure 18 calculated the fluctuations of each residue relative to its average position, which could represent the flexibility and motion intensity of protein amino acids in the whole simulation process. All DHODH complexes displayed a similar residue fluctuation trend with the same fluctuation peaks and valleys. The RMSF values of DHODH complexes were higher in the ranges of 31–50, 215–230, 285–300, and 390–396, suggesting that the residues here fluctuated and the stability was poor (Figure 18b). However, the residues encoding 50–150 were a key binding region, where ligands bind to the protein, signifying good stability due to the low RMSF value. As for the UMPS protein, the difference between these complexes was the variation of degree, and the key binding region (residues encoding 50–90) was also stable. The residue distance matrix (Figure S2) showed the distance between all the residual pairs in the trajectory. We found that the protein binding with different ligands showed a similar residue distance. The reason could be that all these ligands stay steady inside the protein. Furthermore, it was surprising to note that a lot of similarity was revealed between the residue distance matrixes of different proteins.

**Figure 19:**
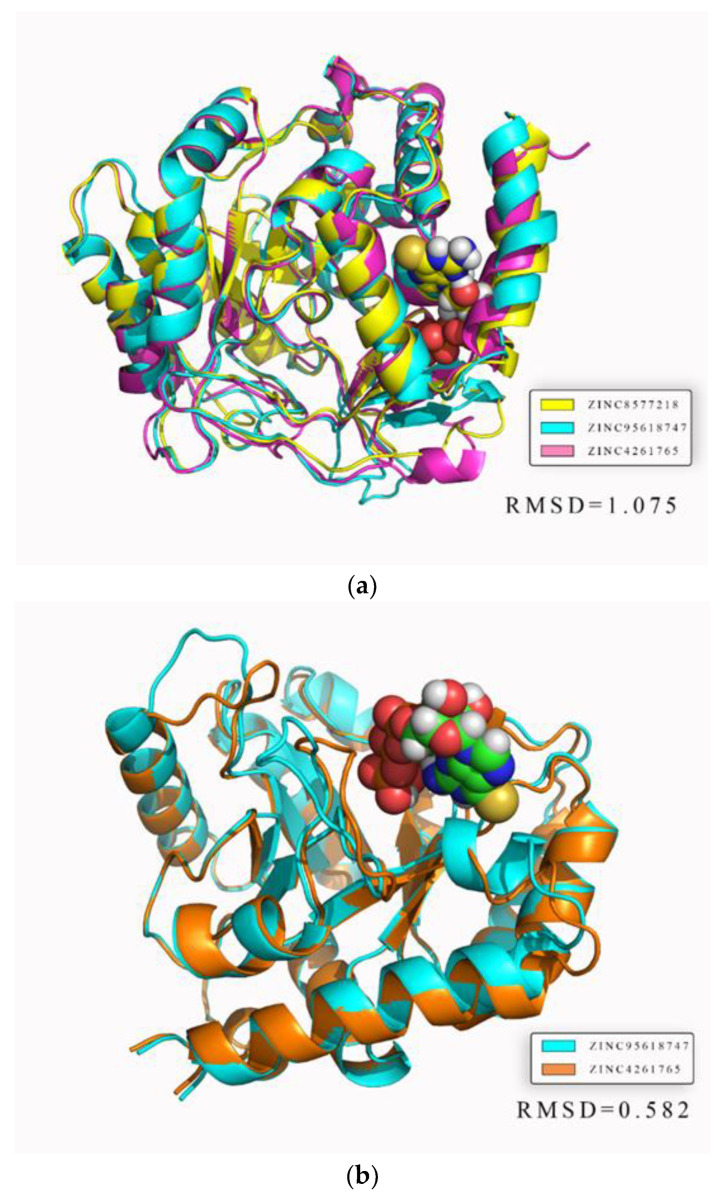
Average structure of each protein reacted with different ligands. The ligand is shown in the figure. (**a**) DHODH protein. (**b**) UMPS protein.

In addition, we focused on the superimposed average protein structures to assess whether the binding sites were stable and reasonable. Both the average structures of DHODH and UMPS reacted with different ligands were nearly superimposed on one structure, with RMSD values of 1.075 and 0.582, respectively (Figure 19). The result illustrates that residues near binding sites were highly overlapped, further demonstrating strong stability and reliability. The areas with less overlapping, where the fluctuations of residues were low, would not affect ligand binding, which was consistent with the RMSF analysis.

**Figure 20:**
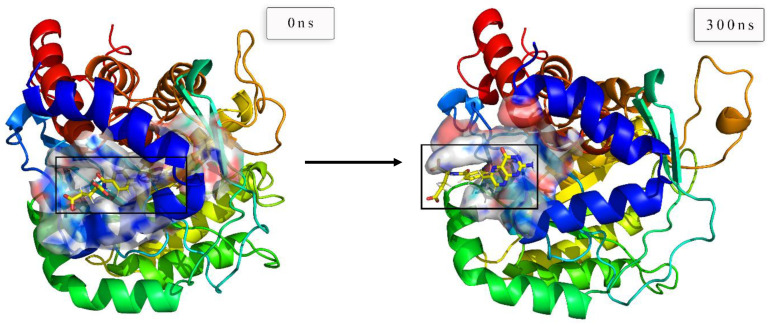

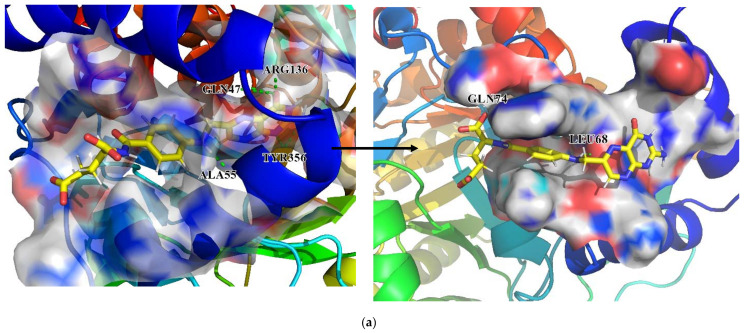

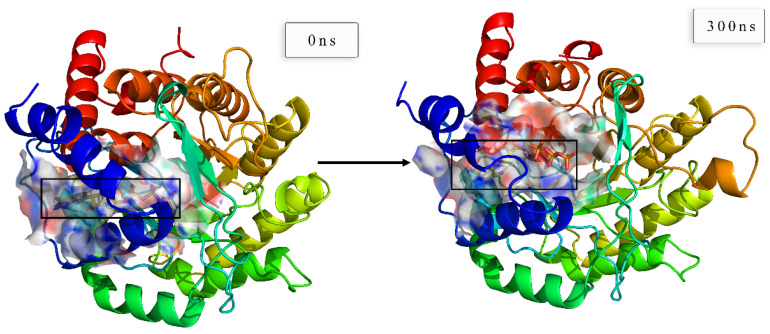

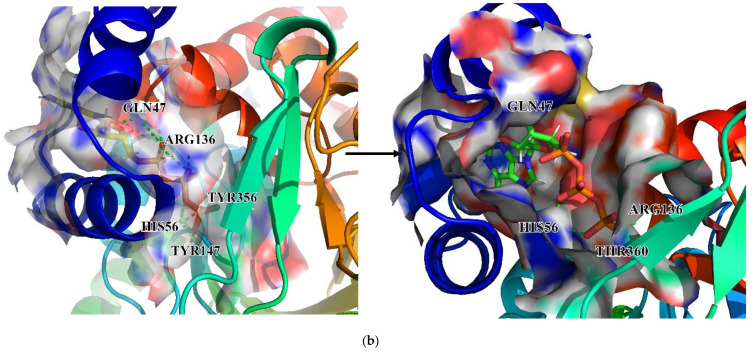

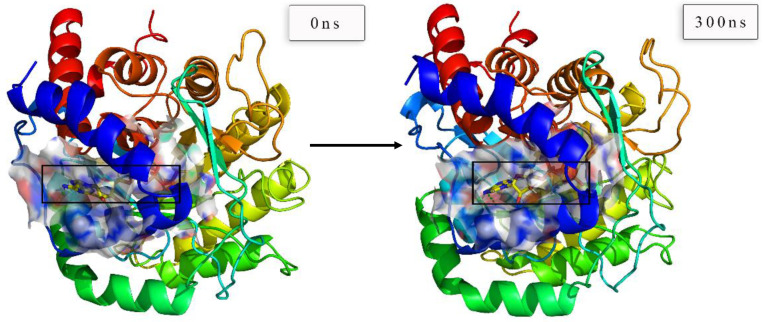

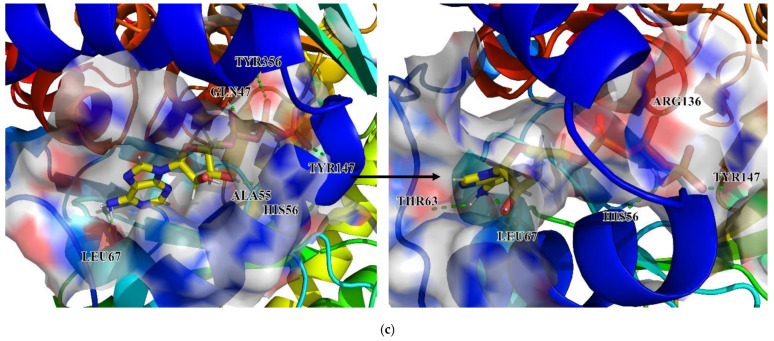
Changes in the initial and final conformations in molecular dynamics simulations. (**a**) DHODH protein with ZINC8577218. (**b**) DHODH protein with ZINC95618747. (**c**) DHODH protein with ZINC4261765.

Finally, changes in the initial and last conformations in MD simulations are shown in Figure 20. As for the DHODH–ZINC8577218 complex, although the ligand was connected with different residues at the beginning and end of the simulation, the critical residue GLN74 was still connected at the end. The other two ligands stayed in the same pocket of the DHODH protein biding with key residues, including GLN74, HIS56, and ARG136, throughout the simulation.

## 4. Conclusions

Overall, in this paper, several potential inhibitors for the DHODH protein were obtained by computer-aided computation. The PPI network revealed that two mainly related proteins, DHODH and its downstream effector protein UMPS, should be focused on in the pyrimidine metabolism pathway. Candidate compounds with strong binding stability screened from molecular docking were further used for multiple artificial intelligence models. We proposed the concept of multi-GNNs and applied it to predict the candidate biological activities with satisfactory results. Additionally, 2D-QSAR models were used to build machine learning prediction models. Furthermore, we expounded the principles and reasons of constructing the multi-GNN models and compared and discussed the performances of multi-GNNs and machine learning models. The results showed that multi-GNNs have great prospects in molecular property prediction and compound screening, attributed to the peculiarity that can capture information from a molecular graph structure directly. How to design and improve the graph information extraction and the model structures could be the key to improve the prediction accuracy of multi-GNNs. Integrating all methodologies and docking results, we finally found that ZINC8577218, ZINC95618747, and ZINC4261765 could be the potentially potent inhibitors for DHODH. MD analysis verified that these compounds show good interactions with both targets. By consulting relevant researches, ZINC8577218 is also known as folic acid that might have some effect on the treatment of cancer. Therefore, the application of artificial intelligence models, especially GNNs, to the discovery and development of multitarget drugs is feasible and reliable and provides a good basis for our further biological experiments to identify and validate the above three inhibitor candidates for DHODH.

## Figures and Tables

**Table 1:** Description of atom features.

Feature	Description	Size
Atom symbol	(C, N, O, S, F, Si, P, Cl, Br, I, H, other) (one-hot)	12
Degree	Number of covalent bonds (0, 1, 2, 3, 4, 5, 6) (one-hot)	7
Hybridization	(sp, sp^2^, sp^3^, sp^3^d, sp^3^d^2^) (one-hot)	5
Valence	Number of implicit valence (0, 1, 2, 3, 4, 5, 6) (one-hot)	7
Formal charge	Integer electronic charge (−1, 0, 1) (one-hot)	3
Atom in ring of size	(3, 4, 5, 6, 7, 8) (one-hot)	6
Aromatic	Whether the atom is part of aromatic systems (0, 1) (integer)	1
Explicit hydrogen	Number of explicit hydrogen (0, 1, 2, 3, 4) (one-hot)	5
		46

**Table 2:** Results of graph neural network (GNN) models and Multi-GNN models.

Name	RMSE on Training Set	R^2^ on Training Set	RMSE on Test Set	R^2^ on Test Set
GIN	0.274	0.953	0.563	0.745
GAT	0.102	0.989	0.606	0.704
ARMA	0.118	0.986	0.604	0.706
SGC	0.092	0.991	0.578	0.731
GIAN	0.323	0.919	0.476	0.818
GIAT	0.316	0.927	0.497	0.801
SGCA	0.277	0.942	0.503	0.796

**Table 3:** Docking score and predicted activity value for the top 10 ZINC candidates.

Rank	Compound	Docking Score	Predicted Activity				
			GIAN	GIAT	SGCA	RF	SVR
1	ZINC8577218	64.296	7.301	7.765	7.235	6.613	5.790
2	ZINC15919406	63.009	6.724	5.593	6.858	6.002	6.384
3	ZINC2036915	62.93	7.608	7.820	6.586	6.611	5.619
4	ZINC3952167	62.463	6.645	6.336	5.167	5.661	6.242
5	ZINC43100953	61.858	5.847	7.543	7.401	5.710	7.242
6	ZINC3831490	61.751	6.627	7.417	7.191	6.074	6.129
7	ZINC4261765	60.522	8.058	10.816	12.787	5.873	5.991
8	ZINC1530605	60.384	5.740	7.980	6.135	7.431	6.689
9	ZINC1587572	59.994	7.608	7.820	6.586	6.664	5.595
10	ZINC95618747	59.621	7.532	10.966	12.392	5.846	6.141

**Table 4:** Vote scores of the top 10 candidates ^a^.

Compound	Docking Score	pIC_50_					Multi-Target	Total Score
		GIAN	GIAT	SGCA	RF	SVR		
ZINC8577218	1	1	0	1	1	0	0	4
ZINC15919406	1	0	0	0	0	1	0	2
ZINC2036915	1	1	1	0	1	0	0	4
ZINC3952167	1	0	0	0	0	1	0	2
ZINC43100953	1	0	0	1	0	1	0	3
ZINC3831490	0	0	0	1	1	0	0	2
ZINC4261765	0	1	1	1	0	0	1	4
ZINC1530605	0	0	1	0	1	1	0	3
ZINC1587572	0	1	1	0	1	0	0	3
ZINC95618747	0	1	1	1	0	1	1	5

^a^ Vote score: for all activity values predicted by one algorithm, the top 50% were voted as 1 point, and others were voted as 0 point.

## Data Availability

The data presented in this study are available in Supplementary Materials Table S1.

## Supplementary Materials

The following are available online at https://www.mdpi.com/2218-273X/11/3/477/s1. Figure S1: 2D horizon of molecular docking results, Figure S2: Residue distance matrix, Table S1: Data sets of dihydroorotate dehydrogenase inhibitors, Table S2: Docking score and predicted activity value for the top 200 zinc candidates.
