# Supplementary material for: A Novel Graph Neural Network Methodology to Investigate Dihydroorotate Dehydrogenase Inhibitors in Small Cell Lung Cancer
Source: Biomolecules. 2021 Mar 23;11(3):477. doi: 10.3390/biom11030477 (PMC8005042; doi:10.3390/biom11030477)
Supplement: Supplementary file 1 [file biomolecules-11-00477-s001.pdf]

**Table S1.** Data sets of dihydroorotate dehydrogenase inhibitors.

| Molecule ChEMBL ID | SMILES | pIC50 |
|---|---|---|
| CHEMBL1076869 | Brc1ccc(cc1)c2csc(N\N=C\c3ccccc3)n2 | 5.48 |
| CHEMBL1084927 | CCC(=O)Nc1ccc(cc1C(=O)O)N(C)Cc2cccnc2 | 5.26 |
| CHEMBL1084928 | CN(Cc1cccnc1)c2ccc(NC(=O)C3CC3)c(c2)C(=O)O | 7 |
| CHEMBL1088742 | OC(=O)c1ccccc1NC(=O)Nc2cc(Cl)cc(Cl)c2 | 6.16 |
| CHEMBL1088743 | OC(=O)c1ccccc1N\N=C\c2oc(cc2)c3ccccc3Cl | 6.3 |
| CHEMBL116206 | Cc1cc(Nc2ccc(cc2)C(F)(F)F)[nH]n1 | 4.78 |
| CHEMBL116946 | Cc1n[nH]c(Nc2ccc(cc2)C(F)(F)F)c1C | 4.65 |
| CHEMBL118125 | Cc1n[nH]c(Nc2c(F)cc(F)cc2F)c1C#N | 4.65 |
| CHEMBL118168 | FC(F)(F)c1ccc(Nc2[nH]nc3CCCCc23)cc1 | 5.21 |
| CHEMBL118395 | Cc1n[nH]c(Nc2ccc(OC(F)(F)F)cc2)c1C#N | 6.3 |
| CHEMBL118468 | CC(C)c1n[nH]c(Nc2ccc(cc2)C(F)(F)F)c1C#N | 5.4 |
| CHEMBL118894 | Cc1n[nH]c(Nc2ccc(cc2)C(F)(F)F)c1CO | 5.57 |
| CHEMBL119801 | Cc1n[nH]c(Nc2ccc(cc2)C#N)c1C#N | 5.5 |
| CHEMBL120640 | Cc1n[nH]c(Nc2ccc(cc2)C(C)(C)C)c1C#N | 5.44 |
| CHEMBL1237007 | OC(=O)c1cccnc1Nc2ccc(Cl)cc2 | 6.4 |
| CHEMBL1332971 | Cc1c(Cl)cccc1Nc2ncccc2C(=O)O | 4.89 |
| CHEMBL135703 | Cc1cc(O)n2nc(SCc3ccccc3)nc2n1 | 4.05 |
| CHEMBL1373924 | COc1cccc(c1)C(=O)Nc2nc3ccccc3s2 | 5.17 |
| CHEMBL141732 | O\C(=C(\C#N)/C(=O)Nc1ccc(cc1)C(F)(F)F)\C2CC2 | 6.93 |
| CHEMBL142996 | O\C(=C(\C#N)/C(=O)Nc1ccc(cc1)c2ccccc2)\C3CC3 | 7.28 |
| CHEMBL1450 | OC1=C([C@@H]2CC[C@H](CC2)c3ccc(Cl)cc3)C(=O)c4ccccc4C1=O | 4.84 |
| CHEMBL1460972 | CCOC(=O)c1sc(Nc2ccc(Br)cc2)nc1C | 5.04 |
| CHEMBL154121 | COc1ccccc1c2cc(F)c(NC(=O)C3=C(CCC3)C(=O)O)c(F)c2 | 7.38 |
| CHEMBL154151 | CN(C)c1ccc(cc1)c2ccc(NC(=O)C3=C(CCC3)C(=O)O)cc2Cl | 7.3 |
| CHEMBL154214 | OC(=O)C1=C(CCC1)C(=O)Nc2ccc(cc2F)c3ccc(OC(F)(F)F)cc3 | 5.24 |
| CHEMBL154336 | OC(=O)C1=C(CCC1)C(=O)Nc2ccc(cc2C#N)c3cccc(OC(F)(F)F)c3 | 6.43 |
| CHEMBL154372 | COc1cccc(c1)c2ccc(NC(=O)C3=C(CCC3)C(=O)O)c(OC)c2 | 6.77 |
| CHEMBL154493 | OC(=O)C1=C(CCC1)C(=O)Nc2ccc(cc2F)c3cccc(OC(F)(F)F)c3 | 6.55 |
| CHEMBL154513 | OC(=O)C1=C(CCC1)C(=O)Nc2c(F)cc(cc2F)c3ccccc3F | 7.35 |
| CHEMBL154613 | CN(C)c1ccc(cc1)c2cc(F)c(NC(=O)C3=C(CCC3)C(=O)O)c(F)c2 | 6.21 |
| CHEMBL154623 | OC(=O)C1=C(CCC1)C(=O)Nc2c(F)c(F)c(c(F)c2F)c3cccc(OC(F)(F)F)c3 | 8.15 |
| CHEMBL154908 | OC(=O)C1=C(CCC1)C(=O)Nc2ccc(cc2Cl)c3ccc(Br)cc3 | 5.54 |
| CHEMBL155530 | OC(=O)C1=C(CCC1)C(=O)Nc2ccc(cc2[N+](=O)[O-])c3cccc(OC(F)(F)F)c3 | 6.41 |
| CHEMBL155548 | OC(=O)C1=C(CCC1)C(=O)Nc2ccc(cc2Cl)c3cccc(OC(F)(F)F)c3 | 6.54 |
| CHEMBL155597 | Cc1cc(ccc1NC(=O)C2=C(CCC2)C(=O)O)c3cccc(OC(F)(F)F)c3 | 6.82 |
| CHEMBL155732 | COc1cc(ccc1NC(=O)C2=C(CCC2)C(=O)O)c3ccccc3Cl | 6.65 |
| CHEMBL157004 | OC(=O)C1=C(CCC1)C(=O)Nc2ccc(cc2C(F)(F)F)c3cccc(OC(F)(F)F)c3 | 6.08 |
| CHEMBL1571058 | OC(=O)c1cc(nc2ccccc12)c3ccc4OCOc4c3 | 5.29 |
| CHEMBL157164 | CC(C)(C)c1ccc(cc1)c2ccc(NC(=O)C3=C(CCC3)C(=O)O)cc2Cl | 7.1 |
| CHEMBL157277 | COc1ccccc1c2ccc(NC(=O)C3=C(CCC3)C(=O)O)c(Cl)c2 | 6.51 |
| CHEMBL1596993 | COc1ccc(Nc2ccccc2C(=O)O)cc1 | 5 |
| CHEMBL1599552 | Cc1cc(O)n2nc(SCc3ccccc3Cl)nc2n1 | 4.92 |

| | | |
|---|---|---|
| CHEMBL1617398 | Cc1c(Nc2ncccc2C(=O)O)cccc1C(F)(F)F | 5.1 |
| CHEMBL1704856 | Cc1cc(O)n2nc(SCc3ccc(Cl)cc3)nc2n1 | 4.41 |
| CHEMBL1929432 | CC\C(=C(/C#N)\C(=O)Nc1ccc(OS(=O)(=O)C(F)(F)F)cc1)\O | 6.82 |
| CHEMBL1929433 | CC\C(=C(/C#N)\C(=O)Nc1ccc(OS(=O)(=O)C(F)(F)F)c(c1)C(=O)OC)\O | 7.03 |
| CHEMBL1929434 | CC\C(=C(/C#N)\C(=O)Nc1ccc(c2ccccc2)c(c1)C(=O)OC)\O | 7.14 |
| CHEMBL1929435 | CC\C(=C(/C#N)\C(=O)Nc1ccc(c2ccc(F)cc2)c(c1)C(=O)OC)\O | 6.96 |
| CHEMBL1929436 | CC\C(=C(/C#N)\C(=O)Nc1ccc(c(c1)C(=O)OC)c2ccccc2Cl)\O | 7.28 |
| CHEMBL1929437 | CC\C(=C(/C#N)\C(=O)Nc1ccc(c2cccc(Cl)c2)c(c1)C(=O)OC)\O | 7.51 |
| CHEMBL1929438 | CC\C(=C(/C#N)\C(=O)Nc1ccc(c2ccc(Cl)cc2)c(c1)C(=O)OC)\O | 7.35 |
| CHEMBL1929439 | CC\C(=C(/C#N)\C(=O)Nc1ccc(c2cccc(OC(F)(F)F)c2)c(c1)C(=O)OC)\O | 7.57 |
| CHEMBL1929440 | CC\C(=C(/C#N)\C(=O)Nc1ccc(c2cccc(F)c2)c(c1)C(=O)OC)\O | 6.7 |
| CHEMBL1929441 | CC\C(=C(/C#N)\C(=O)Nc1ccc(c(c1)C(=O)OC)c2ccc(F)cc2F)\O | 7.51 |
| CHEMBL1929442 | CC\C(=C(/C#N)\C(=O)Nc1ccc(c2cccnc2)c(c1)C(=O)OC)\O | 7.4 |
| CHEMBL1929443 | CC\C(=C(/C#N)\C(=O)Nc1ccc(c(c1)C(=O)OC)c2ccc(F)cc2C)\O | 7.31 |
| CHEMBL1929444 | CC\C(=C(/C#N)\C(=O)Nc1ccc(c2ccc(F)nc2)c(c1)C(=O)OC)\O | 6.82 |
| CHEMBL1929445 | CC\C(=C(/C#N)\C(=O)Nc1ccc(c2ccc(F)c(F)c2)c(c1)C(=O)OC)\O | 7.01 |
| CHEMBL1929446 | CC\C(=C(/C#N)\C(=O)Nc1ccc(c2cc(F)c(F)c(F)c2)c(c1)C(=O)OC)\O | 6.86 |
| CHEMBL1929447 | CC\C(=C(/C#N)\C(=O)Nc1ccc(c(c1)C(=O)OC)c2ccc(F)cc2OC)\O | 7.18 |
| CHEMBL1929448 | CCOc1cccc(c1)c2ccc(NC(=O)\C(=C(/O)\CC)\C#N)cc2C(=O)OC | 7.23 |
| CHEMBL1929449 | CC\C(=C(/C#N)\C(=O)Nc1ccc(c2ccc3OCOc3c2)c(c1)C(=O)OC)\O | 7.12 |
| CHEMBL1929450 | CC\C(=C(/C#N)\C(=O)Nc1ccc(c2cccc(OC)c2)c(c1)C(=O)OC)\O | 7.58 |
| CHEMBL1929451 | CC\C(=C(/C#N)\C(=O)Nc1ccc(c2ccc3OCCOc3c2)c(c1)C(=O)OC)\O | 7.18 |
| CHEMBL1929452 | CC\C(=C(/C#N)\C(=O)Nc1ccc(c2cccc(OC(F)F)c2)c(c1)C(=O)OC)\O | 7.47 |
| CHEMBL1929453 | CC\C(=C(/C#N)\C(=O)Nc1ccc(c2cccc(OC3CCC3)c2)c(c1)C(=O)OC)\O | 7.51 |
| CHEMBL193365 | OC(=O)C1=C(CCC1)C(=O)Nc2ccc(OCc3c(F)cccc3Cl)c(Cl)c2 | 7.96 |
| CHEMBL193371 | OC(=O)C1=C(COC1)C(=O)Nc2ccc(OCc3c(F)cccc3Cl)c(Cl)c2 | 7.39 |
| CHEMBL194023 | COc1cccc(c1)c2ccc(NC(=O)C3=C(CSC3)C(=O)O)c(F)c2 | 6.88 |
| CHEMBL194075 | OC(=O)C1=C(CSC1)C(=O)Nc2c(F)cc(cc2F)c3cccc(OC(F)(F)F)c3 | 7.82 |
| CHEMBL194395 | COc1cccc(c1)c2ccc(NC(=O)C3=C(C(O)CC3)C(=O)O)c(F)c2 | 6.17 |
| CHEMBL194776 | OC(=O)C1=C(CSC1)C(=O)Nc2cc(Br)c(OCc3c(F)cccc3Cl)c(Br)c2 | 6.98 |
| CHEMBL194954 | OC(=O)C1=C(COC1)C(=O)Nc2c(F)c(F)c(c(F)c2F)c3cccc(OC(F)(F)F)c3 | 7.24 |
| CHEMBL194955 | OC(=O)C1=C(CSC1)C(=O)Nc2c(F)c(F)c(c(F)c2F)c3cccc(OC(F)(F)F)c3 | 8.4 |
| CHEMBL195163 | OC(=O)C1=C(COC1)C(=O)Nc2c(F)cc(cc2F)c3cccc(OC(F)(F)F)c3 | 6.69 |
| CHEMBL195246 | OC(=O)C1=C(CSC1)C(=O)Nc2cc(Br)c(OCc3ccccc3)c(Br)c2 | 6.76 |
| CHEMBL195622 | COc1cccc(c1)c2cc(F)c(NC(=O)C3=C(CCC3)C(=O)O)c(F)c2 | 7.96 |
| CHEMBL1956291 | Cc1cc(Nc2ccc(cc2)S(F)(F)(F)(F)F)n3nc(nc3n1)C(F)(F)F | 4.39 |
| CHEMBL1957366 | Oc1c(C#N)c(nc2ccncc12)c3ccc(cc3)c4ccccc4OC(F)(F)F | 6.7 |
| CHEMBL1957370 | Oc1c(C#N)c(nc2ccncc12)c3ccc(cc3)c4ccncc4Cl | 5.2 |
| CHEMBL1957380 | Oc1c(C#N)c(nc2ccncc12)c3ccc(cc3)c4cccnc4 | 5.24 |
| CHEMBL1957460 | Cc1nccc(c1Cl)c2ccc(cc2)c3nc4ccncc4c(O)c3C#N | 5.89 |
| CHEMBL1957462 | Cc1nn(C)c(C)c1c2ccc(cc2)c3nc4ccncc4c(O)c3C#N | 5.96 |
| CHEMBL196173 | OC(=O)C1=C(CS(=O)(=O)C1)C(=O)Nc2ccc(cc2)c3ccccc3 | 5.42 |
| CHEMBL196181 | OC(=O)C1=C(CCC1)C(=O)Nc2cc(Br)c(OCc3c(F)cccc3Cl)c(Br)c2 | 6.96 |
| CHEMBL196256 | COc1cccc(c1)c2ccc(NC(=O)C3=C(CCC3O)C(=O)O)c(F)c2 | 5.84 |

| | | |
|---|---|---|
| CHEMBL197194 | COc1cccc(c1)c2ccc(NC(=O)C3=C(CCC3)C(=O)O)c(F)c2 | 6.87 |
| CHEMBL197455 | COc1cccc(c1)c2ccc(NC(=O)C3=C(COC3)C(=O)O)c(F)c2 | 6.44 |
| CHEMBL197553 | OC(=O)C1=C(CCC1)C(=O)Nc2cc(Br)c(OCc3ccccc3)c(Br)c2 | 6.9 |
| CHEMBL199347 | OC(=O)c1cscc1C(=O)Nc2c(F)cc(cc2F)c3ccccc3OC(F)(F)F | 8 |
| CHEMBL199361 | COc1ccccc1c2c(F)c(F)c(NC(=O)c3ccsc3C(=O)O)c(F)c2F | 9 |
| CHEMBL199501 | OC(=O)c1cscc1C(=O)Nc2ccc(cc2)c3ccccc3 | 6 |
| CHEMBL199574 | OC(=O)c1ccccc1NC(=O)c2ccc3cc(Br)ccc3c2 | 5.08 |
| CHEMBL200536 | OC(=O)c1ccccc1NC(=O)c2ccc3ccccc3c2 | 4.86 |
| CHEMBL200699 | CCOc1cccc(c1)c2cc(F)c(NC(=O)c3ccsc3C(=O)O)c(F)c2 | 8.52 |
| CHEMBL200856 | CCOc1ccccc1c2cc(F)c(NC(=O)c3ccsc3C(=O)O)c(F)c2 | 8.05 |
| CHEMBL200858 | COc1ccccc1c2ccc(NC(=O)c3ccsc3C(=O)O)cc2Cl | 7.92 |
| CHEMBL200895 | CCOc1cccc(c1)c2cc(F)c(NC(=O)c3cocc3C(=O)O)c(F)c2 | 7.8 |
| CHEMBL2012831 | CCCc1nc2ccccc2n1c3ccc(s3)C(=O)NC4CC4 | 4.82 |
| CHEMBL2012961 | Cc1cc(F)c2ncn(c3ccc(s3)C(=O)NC4CC4)c2c1 | 5.51 |
| CHEMBL2012963 | Fc1cc(Cl)c2ncn(c3ccc(s3)C(=O)NC4CC4)c2c1 | 5.25 |
| CHEMBL2012964 | Cc1cc(F)c2c(c1)ncn2c3ccc(s3)C(=O)NC4CC4 | 5.14 |
| CHEMBL2012965 | FC(F)(F)c1ccc2c(c1)ncn2c3ccc(s3)C(=O)NC4CC4 | 4.76 |
| CHEMBL2012966 | Fc1cccc2c1ncn2c3ccc(s3)C(=O)NC4CC4 | 4.83 |
| CHEMBL202431 | OC(=O)c1sccc1C(=O)Nc2c(F)cc(cc2F)c3cccc(OC(F)(F)F)c3 | 8.7 |
| CHEMBL2043303 | OC(=O)c1ccccc1Nc2ccc(Cl)cc2 | 6.38 |
| CHEMBL2177115 | COC(=O)c1ccc(Nc2nc(c(C)s2)c3ccc(Cl)cc3)cc1 | 5.9 |
| CHEMBL2177491 | Fc1ccc(\C=C\C(=O)Nc2ccccn2)cc1 | 4.89 |
| CHEMBL2177492 | OC(=O)c1ccc(\C=N\c2cccc3ccccc23)cc1 | 4.81 |
| CHEMBL2177493 | Cc1ccccc1C(=O)Nc2cc(Cl)c(O)c(Cl)c2 | 4.79 |
| CHEMBL2177494 | Cc1ccc(C)c(c1)\N=C\c2cccc(O)c2O | 4.75 |
| CHEMBL2177495 | N1c2ccccc2Oc3nc4ccccc4nc13 | 4.74 |
| CHEMBL2177853 | Cc1cc(O)n2nc(SCc3ccc(Br)cc3)nc2n1 | 4.5 |
| CHEMBL2177854 | COc1ccc(CSc2nc3nc(C)cc(O)n3n2)cc1 | 4.2 |
| CHEMBL2177855 | Cc1cc(O)n2nc(SCc3ccccc3[N+](=O)[O-])nc2n1 | 5.02 |
| CHEMBL2177856 | Cc1cc(O)n2nc(SCc3ccc(Cl)c(Cl)c3)nc2n1 | 5.19 |
| CHEMBL2177857 | Cc1cc(O)n2nc(SCc3c(Cl)cccc3Cl)nc2n1 | 4.75 |
| CHEMBL2177858 | Cc1cc(O)n2nc(SCc3cc(Cl)ccc3Cl)nc2n1 | 7.29 |
| CHEMBL2177859 | Cc1cc(C)cc(CSc2nc3nc(C)cc(O)n3n2)c1 | 4.52 |
| CHEMBL2177860 | CCc1cc(O)n2nc(SCc3ccc(Cl)cc3)nc2n1 | 4.89 |
| CHEMBL2177861 | CCc1cc(O)n2nc(SCc3cc(Cl)ccc3Cl)nc2n1 | 7.89 |
| CHEMBL2178104 | O=Cc1ccccc1\C=N\Nc2nc(cs2)c3ccccc3 | 6.96 |
| CHEMBL2178105 | ClC(Cl)(Cl)c1cccc(c1)C(=O)Nc2nc3ccccc3s2 | 6.47 |
| CHEMBL2178106 | CCc1cccc(NC(=O)\C=C\c2ccc(Cl)c(Cl)c2)c1 | 6.26 |
| CHEMBL2178107 | Oc1ccc(\C=N\c2cccc3ccccc23)c(O)c1 | 6.11 |
| CHEMBL2178109 | Clc1ccc(C(=O)Nc2nc3ccccc3s2)c(Cl)c1 | 5.74 |
| CHEMBL2178110 | CC1=NN(C(=O)/C/1=C/c2oc(cc2)c3cccc(c3)C(=O)O)c4ccccc4 | 5.56 |
| CHEMBL2178111 | Oc1ccc(cc1)\N=C\c2ccc3ccccc3c2 | 5.4 |
| CHEMBL2178112 | OC(=O)c1cc(\C=C\C(=O)c2ccc(Cl)cc2)ccc1O | 5.22 |
| CHEMBL2178513 | Cc1cc(O)n2nc(SCc3cccc(Cl)c3)nc2n1 | 4.54 |

| | | |
|---|---|---|
| CHEMBL2178514 | Cc1ccc(CSc2nc3nc(C)cc(O)n3n2)cc1 | 4.34 |
| CHEMBL217918 | Cc1ccc2nc(c(O)c(C(=O)O)c2c1)c3ccc(Cl)cc3 | 6.66 |
| CHEMBL217919 | Cc1cccc2nc(c(O)c(C(=O)O)c12)c3ccc(Cl)cc3 | 4.29 |
| CHEMBL217959 | Cc1ccc2c(C(=O)O)c(O)c(nc2c1)c3ccc(Cl)cc3 | 4.77 |
| CHEMBL218051 | Cc1cc(Br)cc2c(C(=O)O)c(O)c(nc12)c3ccc(Cl)cc3 | 4.39 |
| CHEMBL218052 | OC(=O)c1c(O)c(nc2ccc(OC(F)(F)F)cc12)c3ccc(Cl)cc3 | 5.4 |
| CHEMBL218062 | OC(=O)c1c(O)c(nc2ccccc12)c3ccc(cc3)c4ccc(O)cc4 | 8.22 |
| CHEMBL218084 | OC(=O)c1c(O)c(nc2ccc(F)cc12)c3ccc(Oc4ccccc4)cc3 | 8.22 |
| CHEMBL218085 | OC(=O)c1c(O)c(nc2ccc(F)cc12)c3ccc(Sc4ccccc4)cc3 | 7.75 |
| CHEMBL218169 | OC(=O)c1c(O)c(nc2ccccc12)c3ccc(Oc4ccccc4)cc3 | 8.15 |
| CHEMBL218249 | Nc1c(nc2ccc(F)cc2c1C(=O)O)c3ccc(cc3)c4ccccc4 | 7.92 |
| CHEMBL218464 | OC(=O)c1c(O)c(nc2ccc(F)cc12)c3ccc(Cl)cc3 | 6.51 |
| CHEMBL218467 | CCOC(=O)C(=CNc1ccc2sc3ccccc3c2c1)C(=O)OCC | 4.53 |
| CHEMBL218583 | OC(=O)c1c(O)c(nc2c(cccc12)c3ccccc3)c4ccc(Cl)cc4 | 4.58 |
| CHEMBL218700 | OC(=O)c1c(O)c(nc2ccc(Br)cc12)c3ccc(Cl)cc3 | 6.68 |
| CHEMBL219174 | Cc1ccc2c(C(=O)O)c(O)c(nc2c1C)c3ccc(cc3)C(F)(F)F | 4.5 |
| CHEMBL219376 | OC(=O)c1c(O)c(nc2ccccc12)c3ccccc3 | 4.92 |
| CHEMBL219489 | OC(=O)c1c(O)c(nc2ccc(F)cc12)c3ccc(O)cc3 | 5.68 |
| CHEMBL219520 | COc1ccc2nc(c(O)c(C(=O)O)c2c1)c3ccc(Cl)cc3 | 4.37 |
| CHEMBL219648 | OC(=O)c1c(O)c(nc2ccccc12)c3ccc(cc3)c4ccccc4 | 8 |
| CHEMBL220467 | Cc1c(nc2ccc(F)cc2c1C(=O)O)c3ccc(cc3)c4ccccc4 | 8.15 |
| CHEMBL222556 | Oc1ccc(cc1O)C2=CC(=O)c3ccccc3O2 | 4.91 |
| CHEMBL2259675 | OC(=O)c1cc(Cl)cnc1Nc2c(F)cc(cc2F)c3ccccc3 | 8.1 |
| CHEMBL2263447 | OC(=O)c1cc(cnc1Nc2c(F)cc(cc2F)c3ccccc3Cl)C4CC4 | 8.3 |
| CHEMBL2263448 | Cc1c(F)c(Nc2ncccc2C(=O)O)c(F)cc1c3cccc(OC(F)(F)F)c3 | 8.22 |
| CHEMBL2263449 | Cc1c(F)c(Nc2ncccc2C(=O)O)c(F)cc1c3ccccc3Cl | 8.4 |
| CHEMBL2263625 | COc1cccc(c1)c2c(F)c(F)c(Nc3ncccc3C(=O)O)c(F)c2F | 8.52 |
| CHEMBL2263626 | Cc1ccccc1c2cc(F)c(Nc3ncccc3C(=O)O)c(F)c2 | 7.96 |
| CHEMBL2263627 | CCOc1cccc(c1)c2ccc(Nc3ncccc3C(=O)O)c(F)c2 | 6.7 |
| CHEMBL2263628 | COc1cccc(c1)c2cc(F)c(Nc3ncccc3C(=O)O)cc2F | 7.06 |
| CHEMBL2263629 | COc1cccc(c1)c2ccc(Nc3ncccc3C(=O)O)c(Cl)c2 | 6.82 |
| CHEMBL2263630 | OC(=O)c1cccnc1Nc2c(F)cc(cc2F)c3cccc(OC4CCC4)c3 | 7.05 |
| CHEMBL2263631 | CCOc1cccc(c1)c2cc(F)c(Nc3ncccc3C(=O)O)c(F)c2 | 7.72 |
| CHEMBL2263632 | COc1cccc(c1)c2cc(F)c(Nc3ncccc3C(=O)O)c(F)c2 | 7.82 |
| CHEMBL2263636 | Cc1cnc(Nc2ccc(cc2F)c3cccc(OC(F)(F)F)c3)c(c1)C(=O)O | 6.96 |
| CHEMBL2263637 | COc1cccc(c1)c2ccc(Nc3ncc(cc3C(=O)O)C4CC4)c(F)c2 | 7.48 |
| CHEMBL2263638 | COc1cccc(c1)c2cc(F)c(Nc3ncc(C)cc3C(=O)O)c(F)c2 | 7.92 |
| CHEMBL2263639 | COc1cccc(c1)c2cc(F)c(Nc3ncccc3C(=O)O)cc2C | 7 |
| CHEMBL2263640 | CCc1cnc(Nc2c(F)cc(cc2F)c3cccc(OC)c3)c(c1)C(=O)O | 7.92 |
| CHEMBL2263641 | CCOc1cccc(c1)c2cc(F)c(Nc3ncc(cc3C(=O)O)C4CC4)cc2F | 7.64 |
| CHEMBL2263642 | COc1cccc(c1F)c2cc(F)c(Nc3ncccc3C(=O)O)c(F)c2 | 7.28 |
| CHEMBL2263643 | OC(=O)c1cccnc1Nc2c(F)cc(cc2F)c3cccc(OC4CC4)c3 | 7.77 |
| CHEMBL2263644 | Cc1c(F)c(Nc2ncccc2C(=O)O)c(F)cc1c3ccccc3 | 8.3 |
| CHEMBL2263645 | OC(=O)c1cc(cnc1Nc2c(F)cc(cc2F)c3cccc(OC4CC4)c3)C5CC5 | 8.22 |

| | | |
|---|---|---|
| CHEMBL2263646 | OC(=O)c1cc(cnc1Nc2c(F)cc(cc2F)c3cccc(OC(F)(F)F)c3)C4CC4 | 8.4 |
| CHEMBL2335126 | CC(C)c1ccc(cc1)c2nc3ccccc3c(C(=O)O)c2C | 5.8 |
| CHEMBL2385510 | CC(C)(C)c1ccc(Oc2ccc(cc2)c3cc(C(=O)O)c4cc(F)ccc4n3)cc1 | 7.72 |
| CHEMBL2385511 | Cc1cc(cc(C)c1Oc2ccccc2)c3cc(C(=O)O)c4cc(F)ccc4n3 | 7.8 |
| CHEMBL2385512 | CC(C)c1cc(c(C)cc1Oc2ccccc2)c3cc(C(=O)O)c4cc(F)ccc4n3 | 9 |
| CHEMBL2385514 | OC(=O)c1cc(nc2ccc(Cl)cc12)c3ccc(Oc4ccccc4)cc3 | 7.02 |
| CHEMBL2385515 | OC(=O)c1cc(nc2ccc(F)cc12)c3ccc(Oc4ccccc4)cc3 | 7.44 |
| CHEMBL2385715 | CCCOc1ccc(cc1)c2cc(C(=O)O)c3cc(Cl)ccc3n2 | 6.58 |
| CHEMBL2385719 | OC(=O)c1cc(nc2ccc(Cl)cc12)c3ccc(OC(F)(F)F)cc3 | 6.51 |
| CHEMBL2385720 | OC(=O)c1cc(nc2ccc(Cl)cc12)c3ccc(F)cc3 | 5.8 |
| CHEMBL2385721 | Cc1ccc(cc1)c2cc(C(=O)O)c3cc(Cl)ccc3n2 | 5.23 |
| CHEMBL2431484 | CCCNC(=O)C1=C(Nc2ccc(C)c(C)c2)SCC1=O | 5.2 |
| CHEMBL2431485 | CCCCNC(=O)C1=C(Nc2ccc(C)c(C)c2)SCC1=O | 5.35 |
| CHEMBL2431486 | Cc1ccc(NC2=C(C(=O)NC3CC3)C(=O)CS2)cc1C | 5.39 |
| CHEMBL2431487 | Cc1ccc(NC2=C(C(=O)NCC3CC3)C(=O)CS2)cc1C | 5.74 |
| CHEMBL2431513 | CCOC(=O)C1=C(Nc2ccc3CCCc3c2)SCC1=O | 4.92 |
| CHEMBL2431523 | CCCOC(=O)C1=C(Nc2ccc(C)c(C)c2)SCC1=O | 4.93 |
| CHEMBL267210 | Cc1ccc2c(C(=O)O)c(O)c(nc2c1C)c3ccc(Cl)cc3 | 4.14 |
| CHEMBL304401 | CCc1oncc1\C(=N\c2ccc(cc2)C(F)(F)F)\S | 5.52 |
| CHEMBL305215 | S=C(Nc1ccc(cc1)C#N)c2cnoc2C3CC3 | 5.03 |
| CHEMBL306100 | Cc1oncc1\C(=N\c2ccc(cc2)C#N)\S | 4.46 |
| CHEMBL306784 | CCc1oncc1\C(=N\c2ccc(OC(F)(F)F)cc2)\S | 6.16 |
| CHEMBL308141 | FC(F)(F)Oc1ccc(NC(=S)c2cnoc2C3CC3)cc1 | 6.1 |
| CHEMBL3183877 | O=C(CCNC(=O)c1ccc(cc1)C#N)N[C@@H]2CCCc3ccccc23 | 5.05 |
| CHEMBL3184096 | FC(F)(F)c1ccc(cc1)C(=O)NCCC(=O)N[C@@H]2CCCc3ccccc23 | 6.04 |
| CHEMBL3184132 | FC(F)(F)Oc1ccc(cc1)C(=O)NCCC(=O)N[C@@H]2CCCc3ccccc23 | 6.25 |
| CHEMBL3184332 | Cc1ccc(cc1)C(=O)NCCC(=O)N[C@@H]2CCCc3ccccc23 | 5.72 |
| CHEMBL3185911 | Fc1ccc(cc1)C(=O)NCCC(=O)N[C@@H]2CCCc3ccccc23 | 5.02 |
| CHEMBL3186409 | Clc1cccc(c1)C(=O)NCCC(=O)N[C@@H]2CCCc3ccccc23 | 5.13 |
| CHEMBL3186954 | Clc1ccc(cc1)C(=O)NCCC(=O)N[C@@H]2CCCc3ccccc23 | 5.89 |
| CHEMBL3187986 | COc1ccc(cc1)C(=O)NCCC(=O)N[C@@H]2CCCc3ccccc23 | 5.44 |
| CHEMBL3188811 | FC1(F)Oc2ccc(cc2O1)C(=O)NCCC(=O)N[C@@H]3CCCc4ccccc34 | 5.57 |
| CHEMBL3188954 | Clc1ccc(cc1Cl)C(=O)NCCC(=O)N[C@@H]2CCCc3ccccc23 | 5.82 |
| CHEMBL3286441 | COc1ccc2nc(C(=O)Nc3ccc(cc3)c4ccccc4)c(C)c(O)c2c1 | 5.64 |
| CHEMBL3289670 | Cc1cc(Nc2ccc(c(F)c2)C(F)(F)F)n3nc(nc3n1)C(C)(F)F | 4.77 |
| CHEMBL3289671 | Cc1cc(Nc2cc(F)c(c(F)c2)C(F)(F)F)n3nc(nc3n1)C(C)(F)F | 5.68 |
| CHEMBL3289672 | Cc1cc(Nc2cc(F)c(c(F)c2)C(F)(F)F)n3nc(nc3n1)C(F)(F)F | 5.68 |
| CHEMBL3290833 | CCc1ccc2nc(C(=O)Nc3c(F)cc(cc3F)c4cccc(F)c4)c(C)c(O)c2c1 | 5.49 |
| CHEMBL3290836 | COc1ccc2nc(C(=O)Nc3c(F)cc(cc3F)c4cccc(F)c4)c(C)c(O)c2c1 | 6.03 |
| CHEMBL3290838 | Cc1c(O)c2cc(ccc2nc1C(=O)Nc3c(F)cc(cc3F)c4cccc(F)c4)C(F)(F)F | 5.21 |
| CHEMBL3290842 | Cc1c(nc2ccc(F)cc2c1C(=O)O)C(=O)Nc3c(F)cc(cc3F)c4cccc(F)c4 | 5.34 |
| CHEMBL3290843 | CCOc1ccc2nc(C(=O)Nc3c(F)cc(cc3F)c4cccc(F)c4)c(C)c(C(=O)O)c2c1 | 5.55 |
| CHEMBL3290845 | Cc1c(nc2ccc(OC(F)(F)F)cc2c1C(=O)O)C(=O)Nc3c(F)cc(cc3F)c4cccc(F)c4 | 5.82 |
| CHEMBL3290846 | Cc1c(nc2ccc(cc2c1C(=O)O)C(F)(F)F)C(=O)Nc3c(F)cc(cc3F)c4cccc(F)c4 | 5.46 |

| | | |
|---|---|---|
| CHEMBL3290847 | COc1ccc2nc(C(=O)Nc3ccc(cc3)c4ccccc4)c(C)c(C(=O)O)c2c1 | 5.96 |
| CHEMBL330901 | Cc1ccc(Nc2[nH]nc(C)c2C#N)cc1 | 4.92 |
| CHEMBL333344 | Cc1n[nH]c(Nc2ccc(cc2)[N+](=O)[O-])c1C#N | 5.05 |
| CHEMBL333404 | Cc1n[nH]c(Nc2ccc(F)cc2)c1C#N | 4.71 |
| CHEMBL334317 | Cc1n[nH]c(Nc2ccc(cc2)C(F)(F)F)c1C#N | 5.58 |
| CHEMBL3409286 | CC(C)Oc1nn(c(C)c1Cc2ccccc2F)c3ncc(cn3)C4CC4 | 7.9 |
| CHEMBL3409299 | CC(C)Oc1nn(c(C)c1C(O)c2ccccc2)c3ncc(cn3)C4CC4 | 7.8 |
| CHEMBL3409306 | CCOc1nn(c(C)c1Oc2ccccc2Br)c3ncc(CC)cn3 | 6.9 |
| CHEMBL3409491 | CCc1cnc(nc1)n2nc(OC(C)C)c(Oc3c(F)cccc3F)c2C | 9.1 |
| CHEMBL3410571 | CC(=O)c1sc(Nc2ccc(cc2)C(C)(C)C)nc1C | 5.44 |
| CHEMBL3410572 | CC(=O)c1sc(Nc2ccc(C)c(C)c2)nc1C | 5.42 |
| CHEMBL3410574 | CC(=O)c1sc(Nc2ccc(Cl)c(c2)C(F)(F)F)nc1C | 5.63 |
| CHEMBL3410575 | CC(=O)c1sc(Nc2ccc(Br)c(c2)C(F)(F)F)nc1C | 5.71 |
| CHEMBL3410576 | CC(=O)c1sc(Nc2ccc3ccccc3c2)nc1C | 6.25 |
| CHEMBL3410578 | CCOC(=O)c1sc(Nc2ccc(C)c(C)c2)nc1C | 5.87 |
| CHEMBL3410579 | CCOC(=O)c1sc(Nc2ccc(C)c(F)c2)nc1C | 5.7 |
| CHEMBL3410580 | CCOC(=O)c1sc(Nc2ccc(C)c(Cl)c2)nc1C | 6.01 |
| CHEMBL3410581 | CCOC(=O)c1sc(Nc2ccc3ccccc3c2)nc1C | 6.05 |
| CHEMBL3410582 | CCOC(=O)c1sc(Nc2ccc(C)c(C)c2)nc1N | 5.68 |
| CHEMBL3410585 | CCOC(=O)c1sc(Nc2ccc(Cl)c(c2)C(F)(F)F)nc1NC(=O)C3CC3 | 5.22 |
| CHEMBL3410586 | CCOC(=O)c1sc(Nc2ccc(C)c(C)c2)nc1NC(=O)c3ccccc3 | 5.04 |
| CHEMBL3410588 | CCOC(=O)c1sc(Nc2ccc(C)c(C)c2)nc1C3CC3 | 6.49 |
| CHEMBL3410589 | CCOC(=O)c1sc(Nc2ccc(C)c(F)c2)nc1C3CC3 | 6.19 |
| CHEMBL3410590 | CCOC(=O)c1sc(Nc2ccc(C)c(Cl)c2)nc1C3CC3 | 6.43 |
| CHEMBL3410591 | CCOC(=O)c1sc(Nc2ccc(C)c(C)c2)nc1C(C)(C)C | 6.29 |
| CHEMBL3410592 | CCOC(=O)c1sc(Nc2ccc(C)c(F)c2)nc1C(C)(C)C | 6.19 |
| CHEMBL3410593 | CCOC(=O)c1sc(Nc2ccc(C)c(Cl)c2)nc1C(C)(C)C | 6.26 |
| CHEMBL3410594 | CCOC(=O)c1sc(Nc2ccc(C)c(C)c2)nc1c3ccccc3 | 7.24 |
| CHEMBL3410595 | CCOC(=O)c1sc(Nc2ccc(C)c(F)c2)nc1c3ccccc3 | 7.31 |
| CHEMBL3410596 | CCOC(=O)c1sc(Nc2ccc(C)c(Cl)c2)nc1c3ccccc3 | 7.46 |
| CHEMBL3410597 | CCOC(=O)c1sc(Nc2ccccc2)nc1c3ccccc3 | 6.11 |
| CHEMBL3410598 | CCOC(=O)c1sc(Nc2ccc(OC)cc2)nc1c3ccccc3 | 5.84 |
| CHEMBL3410599 | CCOC(=O)c1sc(Nc2ccc(Cl)cc2)nc1c3ccccc3 | 6.62 |
| CHEMBL3410600 | CCOC(=O)c1sc(Nc2ccc(cc2)C(F)(F)F)nc1c3ccccc3 | 6.39 |
| CHEMBL3410601 | CCOC(=O)c1sc(Nc2ccc(cc2)C(C)(C)C)nc1c3ccccc3 | 6.55 |
| CHEMBL3410602 | CCOC(=O)c1sc(Nc2ccc(Cl)c(c2)C(F)(F)F)nc1c3ccccc3 | 6.88 |
| CHEMBL3410603 | CCOC(=O)c1sc(Nc2ccc(Br)c(c2)C(F)(F)F)nc1c3ccccc3 | 6.89 |
| CHEMBL3410604 | CCOC(=O)c1sc(Nc2ccc(Cl)c(Cl)c2)nc1c3ccccc3 | 6.76 |
| CHEMBL3410605 | CCOC(=O)c1sc(Nc2cc(Cl)cc(Cl)c2)nc1c3ccccc3 | 6.41 |
| CHEMBL3410607 | CCOC(=O)c1sc(Nc2ccc3CCCc3c2)nc1c4ccccc4 | 7.58 |
| CHEMBL3410608 | CCOC(=O)c1sc(Nc2ccc3OCOc3c2)nc1c4ccccc4 | 5.96 |
| CHEMBL3410610 | CCOC(=O)c1sc(Nc2ccc3ccccc3c2)nc1c4ccccc4 | 7.54 |
| CHEMBL3410611 | CCOC(=O)c1sc(Nc2ccc3cc4ccccc4cc3c2)nc1c5ccccc5 | 6.34 |
| CHEMBL3410616 | CCOC(=O)c1sc(Nc2c(F)cc(cc2F)c3cccc(OC)c3)nc1c4ccccc4 | 6.51 |

| | | |
|---|---|---|
| CHEMBL345147 | OC(=O)C1=C(CCC1)C(=O)Nc2c(F)cc(cc2F)c3ccccc3Cl | 7.75 |
| CHEMBL345446 | OC(=O)C1=C(CCC1)C(=O)Nc2ccc(cc2F)c3ccc(Br)cc3 | 6.13 |
| CHEMBL345464 | OC(=O)C1=C(CCC1)C(=O)Nc2ccc(c(Cl)c2)c3cccc(OC(F)(F)F)c3 | 7.7 |
| CHEMBL345685 | OC(=O)C1=C(CCC1)C(=O)Nc2ccc(cc2F)c3cccc(O)c3 | 6.21 |
| CHEMBL345701 | COc1ccccc1c2c(F)c(F)c(NC(=O)C3=C(CCC3)C(=O)O)c(F)c2F | 8.1 |
| CHEMBL347148 | COc1ccc(cc1)c2ccc(NC(=O)C3=C(CCC3)C(=O)O)c(F)c2 | 5.77 |
| CHEMBL348270 | OC(=O)C1=C(CCC1)C(=O)Nc2ccc(cc2)c3ccccc3 | 6.39 |
| CHEMBL348871 | OC(=O)C1=C(CCC1)C(=O)Nc2ccc(c(Cl)c2)c3ccc(Br)cc3 | 7.16 |
| CHEMBL349577 | OC(=O)C1=C(CCC1)C(=O)Nc2ccc(cc2C(F)(F)F)c3cccc4ccccc34 | 6.75 |
| CHEMBL351786 | CCOc1cccc(c1)c2cc(F)c(NC(=O)C3=C(CCC3)C(=O)O)c(F)c2 | 7.77 |
| CHEMBL358435 | COc1ccc(cc1)c2ccc(NC(=O)C3=C(CCC3)C(=O)O)c(Cl)c2 | 5.08 |
| CHEMBL3589752 | CC(C)Oc1nn(c(C)c1Oc2c(F)cccc2F)c3ncc(cc3F)C4CC4 | 7.6 |
| CHEMBL3593251 | CC(=O)Nc1ccc(cc1)c2ccc(cc2)c3nc4c(cc(C)cc4[nH]3)C(=O)O | 6.68 |
| CHEMBL3593802 | CC(=O)Nc1cccc(c1)c2ccc(cc2)c3nc4c(cc(C)cc4[nH]3)C(=O)O | 5.86 |
| CHEMBL3593803 | CC(=O)Nc1ccccc1c2ccc(cc2)c3nc4c(cc(C)cc4[nH]3)C(=O)O | 4.5 |
| CHEMBL3593804 | CC(=O)Nc1ccccc1COc2ccc(cc2)c3ccc(cc3)c4nc5c(cc(C)cc5[nH]4)C(=O)O | 6.52 |
| CHEMBL3593805 | CC(=O)Nc1cccc(COc2ccc(cc2)c3ccc(cc3)c4nc5c(cc(C)cc5[nH]4)C(=O)O)c1 | 7.55 |
| CHEMBL3593806 | CC(=O)Nc1ccc(COc2ccc(cc2)c3ccc(cc3)c4nc5c(cc(C)cc5[nH]4)C(=O)O)cc1 | 7.89 |
| CHEMBL3593807 | CC(=O)Nc1cc(ccc1c2ccccc2)c3nc4c(cc(C)cc4[nH]3)C(=O)O | 4.33 |
| CHEMBL3593808 | Cc1cc(C(=O)N)c2nc([nH]c2c1)c3ccc(cc3)c4ccccc4 | 5.11 |
| CHEMBL3593812 | NC(=O)c1cccc2[nH]c(nc12)c3ccc(cc3)c4ccc(N)nc4 | 4.7 |
| CHEMBL3593813 | NC(=O)c1cccc2[nH]c(nc12)c3ccc(cc3)c4cncc(c4)C(=O)N5CCCC5 | 4.55 |
| CHEMBL3593981 | Cc1cc(C(=O)N)c2nc([nH]c2c1)c3ccc(cc3)c4cncc(c4)C(=O)N5CCCC5 | 4.3 |
| CHEMBL3593983 | Cc1cc(C(=O)N)c2nc([nH]c2c1)c3ccc(cc3)c4ccc(nc4)C(=O)N5CCCC5 | 4.43 |
| CHEMBL3593984 | CC(=O)Nc1ccccc1c2ccc(cc2)c3nc4c(cccc4[nH]3)C(=O)N | 4.29 |
| CHEMBL3593985 | CC(=O)Nc1cccc(c1)c2ccc(cc2)c3nc4c(cccc4[nH]3)C(=O)N | 4.38 |
| CHEMBL3593986 | CC(=O)Nc1ccc(cc1)c2ccc(cc2)c3nc4c(cccc4[nH]3)C(=O)N | 4.25 |
| CHEMBL3593987 | NC(=O)c1cccc2[nH]c(nc12)c3ccc(cc3)c4ccc(cc4)C(=O)N5CCCC5 | 4.7 |
| CHEMBL3593989 | CC(=O)Nc1ccc(cn1)c2ccc(cc2)c3nc4c(cccc4[nH]3)C(=O)N | 4.4 |
| CHEMBL3593990 | NC(=O)c1cccc2[nH]c(nc12)c3ccc(cc3)c4cncc(CN5CCCC5)c4 | 4.26 |
| CHEMBL3593992 | NC(=O)c1cccc2[nH]c(nc12)c3ccc(cc3)c4ccc(OCCN5CCCCC5)cc4 | 4.51 |
| CHEMBL3593993 | NC(=O)c1cc(F)cc2[nH]c(nc12)c3ccc(cc3F)c4ccc(NC(=O)C5CC5)cc4F | 4.32 |
| CHEMBL3593995 | NC(=O)c1cc(F)cc2[nH]c(nc12)c3ccc(cc3F)c4cccc(NC(=O)C5CC5)c4 | 4.42 |
| CHEMBL3593996 | COc1cc(ccc1NC(=O)C2CC2)c3ccc(c(F)c3)c4nc5c(cc(F)cc5[nH]4)C(=O)N | 4.64 |
| CHEMBL3593998 | OC(=O)c1cccc2[nH]c(nc12)c3ccc(cc3)c4ccccc4 | 6.12 |
| CHEMBL3639735 | Cc1ccc(Nc2cnc(c(C)c2)c3ccccc3C(F)(F)F)c(c1)C(=O)O | 7.7 |
| CHEMBL3639766 | Cc1cc(C(=O)O)c2nc([nH]c2c1)c3c(F)c(F)c(c(F)c3F)c4ccccc4F | 8.46 |
| CHEMBL3674625 | OC(=O)c1cc(ccc1Nc2cnc(nc2)c3ccccc3)C4CC4 | 6.98 |
| CHEMBL3674626 | Cc1ccc(Nc2ccc(nc2)c3cccc(c3)C(F)(F)F)c(c1)C(=O)O | 7.01 |
| CHEMBL3674627 | OC(=O)c1cc(ccc1Nc2cnc(nc2)c3c(F)cc(O)cc3F)C4CC4 | 7.24 |
| CHEMBL3674628 | COc1ncc(Nc2ccc(cc2C(=O)O)C3CC3)cc1c4ccccc4 | 7.31 |
| CHEMBL3674629 | Cc1ccc(Nc2cnc(c(F)c2)c3ccccc3)c(c1)C(=O)O | 7.75 |
| CHEMBL3674630 | OC(=O)c1cc(ccc1Nc2cnc(nc2)c3ccccc3C4CC4)C5CC5 | 7.24 |
| CHEMBL3674631 | OC(=O)c1cc(ccc1Nc2cncc(c2)c3ccccc3)C4CC4 | 6.95 |

| | | |
|---|---|---|
| CHEMBL3674632 | Cc1ccc(Nc2cc(Cl)cnc2c3ccccc3)c(c1)C(=O)O | 7.21 |
| CHEMBL3674633 | OC(=O)c1cc(ccc1Nc2cnc(c3ccccc3)c(c2)c4ccccc4)C5CC5 | 8 |
| CHEMBL3674634 | OC(=O)c1cc(ccc1Nc2cnc(nc2)c3cccc(OC4CC4)c3)C5CC5 | 6.94 |
| CHEMBL3674635 | OC(=O)c1cc(ccc1Nc2cnc(C3CC3)c(c2)c4ccccc4)C5CC5 | 7.55 |
| CHEMBL3674636 | Cc1ccc(Nc2cnc(N3CCCCC3)c(C)c2)c(c1)C(=O)O | 7.39 |
| CHEMBL3674637 | Cc1ccc(Nc2cnc(N3CCCCCC3)c(C)c2)c(c1)C(=O)O | 6.92 |
| CHEMBL3674638 | Cc1ccc(Nc2ccc(nc2)c3ccncc3Cl)c(c1)C(=O)O | 6.96 |
| CHEMBL3674639 | CCOc1cccc(c1)c2ccc(Nc3ccc(C)cc3C(=O)O)cn2 | 6.72 |
| CHEMBL3674640 | CCOc1cccc(c1)c2ncc(Nc3ccc(C)cc3C(=O)O)cc2C | 7.52 |
| CHEMBL3674641 | CCOc1cccc(c1)c2cc(C)c(Nc3ccc(C)cc3C(=O)O)cn2 | 7.11 |
| CHEMBL3674642 | COc1cccc(c1)c2ncc(Nc3ccc(cc3C(=O)O)C(F)(F)F)cc2C | 6.86 |
| CHEMBL3674643 | COc1cccc(c1)c2ncc(Nc3ccc(C)cc3C(=O)O)cc2C | 7.68 |
| CHEMBL3674644 | CCOc1ccc(F)c(c1)c2ccc(Nc3ccc(C)cc3C(=O)O)cn2 | 7.72 |
| CHEMBL3674645 | Cc1ccc(Nc2ccc(nc2)c3ccccc3F)c(c1)C(=O)O | 7.04 |
| CHEMBL3674646 | Cc1ccc(Nc2cnc(c(C)c2)c3ccccc3)c(c1)C(=O)O | 7.28 |
| CHEMBL3674647 | CC(C)Oc1ccc(F)c(c1)c2ccc(Nc3ccc(C)cc3C(=O)O)cn2 | 7.55 |
| CHEMBL3674648 | CC(C)Oc1cccc(c1)c2ncc(Nc3ccc(C)cc3C(=O)O)cc2C | 7.96 |
| CHEMBL3674649 | Cc1ccc(Nc2cnc(c(C)c2)c3ccccc3Cl)c(c1)C(=O)O | 7.85 |
| CHEMBL3674650 | Cc1ccc(Nc2cnc(c(C)c2)c3cccc(c3)C(=O)N)c(c1)C(=O)O | 6.72 |
| CHEMBL3674651 | COc1ccc(F)c(c1)c2cc(C)c(Nc3ccc(C)cc3C(=O)O)cn2 | 7.01 |
| CHEMBL3674652 | COc1cccc(c1)c2ncc(Nc3ccc(C)cc3C(=O)O)cc2C(F)(F)F | 7.92 |
| CHEMBL3674653 | CN(C)C(=O)c1cccc(c1)c2ncc(Nc3ccc(C)cc3C(=O)O)cc2C | 7.48 |
| CHEMBL3674654 | Cc1ccc(Nc2ccc(nc2)c3ccccc3Cl)c(c1)C(=O)O | 7.5 |
| CHEMBL3674655 | Cc1cc(Nc2ccc(cc2C(=O)O)C3CC3)cnc1c4cccc(OC(F)(F)F)c4 | 8.3 |
| CHEMBL3674656 | Cc1cc(Nc2ccc(cc2C(=O)O)C3CC3)cnc1c4ccccc4 | 8.22 |
| CHEMBL3674657 | Cc1ccc(Nc2cnc(c(C)c2)c3cccc(Cl)c3)c(c1)C(=O)O | 8 |
| CHEMBL3674658 | Cc1ccc(Nc2cnc(c(C)c2)c3ccccc3F)c(c1)C(=O)O | 8.3 |
| CHEMBL3674659 | Cc1ccc(Nc2cnc(c(C)c2)c3ccncc3F)c(c1)C(=O)O | 7.43 |
| CHEMBL3674660 | OC(=O)c1cc(ccc1Nc2cnc(c3ccccc3)c(c2)C(F)(F)F)C4CC4 | 8.7 |
| CHEMBL3674661 | COc1cccc(c1)c2ncc(Nc3ccc(cc3C(=O)O)C4CC4)cc2C(F)(F)F | 8.15 |
| CHEMBL3674662 | OC(=O)c1cc(Cl)ccc1Nc2ccc(nc2)c3ccccc3Cl | 6.84 |
| CHEMBL3674663 | OC(=O)c1cc(ccc1Nc2ccc(nc2)c3ccccc3Cl)C4CC4 | 8.4 |
| CHEMBL3674664 | Cc1ccc(Nc2ccc(nc2)c3ccncc3F)c(c1)C(=O)O | 7.05 |
| CHEMBL3674665 | Cc1ccc(Nc2ccc(nc2)c3c(F)cccc3F)c(c1)C(=O)O | 7.72 |
| CHEMBL3674666 | OC(=O)c1cc(ccc1Nc2cnc(nc2)c3ccccc3Cl)C4CC4 | 8.52 |
| CHEMBL3674667 | Cc1ccc(Nc2cnc(nc2)c3ccccc3Cl)c(c1)C(=O)O | 7.24 |
| CHEMBL3674668 | Cc1ccc(Nc2cnc(c(C)c2)c3cccc(c3)C(=O)N4CCCC4)c(c1)C(=O)O | 8.05 |
| CHEMBL3674669 | Cc1ccc(Nc2cnc(c(C)c2)c3cccc(c3)C(=O)NC4CC4)c(c1)C(=O)O | 7.92 |
| CHEMBL3674670 | OC(=O)c1cc(ccc1Nc2cnc(nc2)c3ccccc3F)C4CC4 | 8 |
| CHEMBL3674671 | OC(=O)c1cc(ccc1Nc2cnc(nc2)c3ccccc3C(F)(F)F)C4CC4 | 8.05 |
| CHEMBL3674672 | Cc1ccccc1c2ncc(Nc3ccc(cc3C(=O)O)C4CC4)cn2 | 7.92 |
| CHEMBL3674673 | OC(=O)c1cc(ccc1Nc2cnc(nc2)c3cc(F)ccc3F)C4CC4 | 7.42 |
| CHEMBL3674674 | OC(=O)c1cc(ccc1Nc2cnc(nc2)c3cc(ccc3F)C(F)(F)F)C4CC4 | 7.68 |
| CHEMBL3674675 | OC(=O)c1cc(ccc1Nc2ccc(nc2)c3ccccc3F)C4CC4 | 8.1 |

| | | |
|---|---|---|
| CHEMBL3674676 | Cc1ccc(Nc2ccc(nc2)c3c(F)ccc(F)c3F)c(c1)C(=O)O | 7.11 |
| CHEMBL3674677 | COc1ccc(F)cc1c2ncc(Nc3ccc(C)cc3C(=O)O)cc2C | 7.34 |
| CHEMBL3674678 | Cc1ccc(Nc2ccc(nc2)c3ccc(F)cc3F)c(c1)C(=O)O | 6.84 |
| CHEMBL3677726 | OC(=O)c1cccc2[nH]c(nc12)c3ccc(OCc4ccccc4)cc3 | 5.3 |
| CHEMBL3677727 | COc1cccc(c1)c2ccc(c(F)c2)c3nc4c(cccc4[nH]3)C(=O)O | 6.21 |
| CHEMBL3677728 | OC(=O)c1cc(Br)cc2[nH]c(nc12)c3ccc(cc3)c4ccccc4 | 6.24 |
| CHEMBL3677729 | Cn1c(nc2cccc(C(=O)O)c12)c3ccc(cc3)c4ccccc4 | 5.8 |
| CHEMBL3677730 | OC(=O)c1cccc2[nH]c(nc12)c3cccc(Oc4ccccc4)c3 | 5.14 |
| CHEMBL3677731 | OC(=O)c1cccc2[nH]c(nc12)c3ccc(Oc4ccccc4)cc3 | 5.54 |
| CHEMBL3677732 | Cc1cc(C(=O)O)c2nc([nH]c2c1)c3ccc(cc3)c4ccccc4 | 6.52 |
| CHEMBL3677733 | OC(=O)c1cccc2[nH]c(nc12)c3ccc(cc3)c4ccccc4F | 6.31 |
| CHEMBL3677734 | OC(=O)c1cccc2[nH]c(nc12)c3ccc(cc3)c4cccc(F)c4 | 5.42 |
| CHEMBL3677735 | OC(=O)c1cccc2[nH]c(nc12)c3ccc(cc3)c4cccc(OC(F)(F)F)c4 | 5.9 |
| CHEMBL3677736 | OC(=O)c1cccc2nc(oc12)c3ccc(cc3)c4ccccc4 | 5.52 |
| CHEMBL3677737 | COc1cc(cc(OC)c1c2ccccc2)c3nc4c(cccc4[nH]3)C(=O)O | 6.01 |
| CHEMBL3677738 | OC(=O)c1cccc2[nH]c(nc12)c3c(F)c(F)c(c(F)c3F)c4ccccc4 | 7.42 |
| CHEMBL3677739 | OC(=O)c1cccc2nc([nH]c12)c3ccc(cc3F)c4ccccc4F | 6.01 |
| CHEMBL3677740 | Cc1cc(C(=O)O)c2[nH]c(nc2c1)c3ccc(cc3F)c4ccccc4F | 6.76 |
| CHEMBL3677741 | Cc1cc(C(=O)O)c2[nH]c(nc2c1)c3ccc(cc3)c4ccccc4F | 6.43 |
| CHEMBL3677742 | OC(=O)c1cccc2nc([nH]c12)c3ccc(cc3F)c4ccccc4 | 6.04 |
| CHEMBL3677743 | Cc1cc(C(=O)O)c2[nH]c(nc2c1)c3ccc(cc3F)c4ccccc4 | 6.39 |
| CHEMBL3677744 | OC(=O)c1cc(F)cc2[nH]c(nc12)c3ccc(cc3F)c4ccccc4 | 6.21 |
| CHEMBL3677745 | Cc1cc(C(=O)O)c2[nH]c(nc2c1)c3ccc(cc3F)c4ccc(F)cc4F | 6 |
| CHEMBL3677746 | Cc1cc(C(=O)O)c2[nH]c(nc2c1)c3ccc(cc3)c4ccc(F)cc4F | 5.85 |
| CHEMBL3677747 | OC(=O)c1cc(Cl)cc2nc([nH]c12)c3ccc(cc3)c4ccccc4 | 6 |
| CHEMBL3677748 | Cc1cc(C(=O)O)c2nc([nH]c2c1)c3c(F)c(F)c(c(F)c3F)c4ccccc4 | 8.17 |
| CHEMBL3677749 | OC(=O)c1cc(Cl)cc2[nH]c(nc12)c3ccc(cc3F)c4ccccc4 | 6.18 |
| CHEMBL3677750 | OC(=O)c1cc(Cl)cc2[nH]c(nc12)c3ccc(cc3)c4ccccc4F | 6.02 |
| CHEMBL3677751 | COc1cc(ccc1c2nc3c(cc(C)cc3[nH]2)C(=O)O)c4ccccc4 | 5.93 |
| CHEMBL3677752 | Cc1cc(C(=O)O)c2nc([nH]c2c1)c3c(F)cc(cc3F)c4ccccc4 | 6.75 |
| CHEMBL3677753 | OC(=O)c1cc(Cl)cc2CC(=Nc12)c3ccc(cc3)c4ccccc4 | 5.63 |
| CHEMBL3677754 | Cc1cc(C(=O)O)c2nc([nH]c2c1)c3ccc(cc3)c4cccc(F)c4F | 6.05 |
| CHEMBL3677755 | Cc1cc(C(=O)O)c2nc([nH]c2c1)c3ccc(cn3)c4ccccc4 | 5.15 |
| CHEMBL3677756 | OC(=O)c1cccc2[nH]c(nc12)c3ccc(Nc4ccccc4)cc3 | 4.57 |
| CHEMBL3677757 | OC(=O)c1cccc2[nH]c(nc12)c3ccc(Oc4c(F)cccc4F)cc3 | 5.8 |
| CHEMBL3677758 | OC(=O)c1cc(F)cc2[nH]c(nc12)c3ccc(cc3)c4cccnc4 | 6.36 |
| CHEMBL3677759 | Cc1cc(C(=O)O)c2nc([nH]c2c1)c3ccc(cc3)c4cccnc4 | 6.39 |
| CHEMBL3677760 | OC(=O)c1cccc2cc([nH]c12)c3ccc(cc3)c4ccccc4 | 6.22 |
| CHEMBL3677761 | OC(=O)c1cccc2nc([nH]c12)c3ccc(cc3)c4c(F)cccc4F | 5.74 |
| CHEMBL3677762 | Cc1cc(C(=O)O)c2[nH]c(nc2c1)c3ccc(Oc4ccccc4)cc3 | 5.47 |
| CHEMBL3677763 | OC(=O)c1cc(F)cc2[nH]c(nc12)c3c(F)cc(cc3F)c4ccccc4 | 6.56 |
| CHEMBL3677764 | Cc1cc(C(=O)O)c2nc([nH]c2c1)c3ccc(Oc4ccccn4)cc3 | 5.36 |
| CHEMBL3677765 | Cc1cc(C(=O)O)c2[nH]c(nc2c1)c3ccc(nc3)c4ccccc4 | 5.42 |
| CHEMBL3677766 | Cc1cc(C(=O)O)c2[nH]c(nc2c1)c3ccc(cc3)c4c(F)cccc4F | 6.46 |

| | | |
|---|---|---|
| CHEMBL3677767 | Cc1cc(C(=O)O)c2[nH]c(nc2c1)c3c(F)c(F)c(c(F)c3F)n4c(C)ccc4C | 8.25 |
| CHEMBL3677768 | Cc1cc(C(=O)O)c2nc([nH]c2c1)c3c(F)c(F)c(c(F)c3F)c4ccc(CN5CCCCC5)cc4 | 7.11 |
| CHEMBL3677769 | Cc1cc(C(=O)O)c2nc(C#Cc3ccccc3)[nH]c2c1 | 4.94 |
| CHEMBL3677770 | Cc1cc(C(=O)O)c2nc([nH]c2c1)c3c(F)c(F)c(c(F)c3F)c4ccc(NC(=O)C5CCCN5)cc4 | 7.25 |
| CHEMBL3677771 | Cc1cc(C(=O)O)c2nc([nH]c2c1)c3c(F)c(F)c(c(F)c3F)c4cccc(NC(=O)C5CCCN5)c4 | 6.09 |
| CHEMBL3677772 | Cc1cc(C(=O)O)c2nc([nH]c2c1)c3ccc(cc3)c4ccc(OCCCN5CCOCC5)cc4 | 5.52 |
| CHEMBL3677773 | Cc1cc(C(=O)O)c2nc([nH]c2c1)c3ccc(cc3)c4ccc(OCCCN5CCCCC5)cc4 | 5.79 |
| CHEMBL3677774 | Cc1cc(C(=O)O)c2nc([nH]c2c1)c3ccc(cc3)c4ccc(NC(=O)C5CCCN5)cc4 | 6.16 |
| CHEMBL3677775 | Cc1cc(C(=O)O)c2nc([nH]c2c1)c3ccc(cc3)c4ccc(NC(=O)C5CCNCC5)cc4 | 5.43 |
| CHEMBL3677776 | Cc1cc(C(=O)O)c2[nH]c(nc2c1)c3ccc(cc3)c4ccc(O)c(O)c4 | 6.19 |
| CHEMBL3677777 | Cc1cc(C(=O)O)c2nc([nH]c2c1)c3ccc(cc3)c4ccc(cc4)C(=O)NC5CCNCC5 | 5.18 |
| CHEMBL3677778 | Cc1cc(C(=O)O)c2nc(c3ccc(cc3)c4ccccc4)n(CCO)c2c1 | 5.32 |
| CHEMBL3677779 | Cc1cc(C(=O)O)c2c(c1)nc(c3c(F)c(F)c(c(F)c3F)c4ccc(O)cc4)n2CCO | 7.11 |
| CHEMBL3677780 | Cc1cc(C(=O)O)c2[nH]c(nc2c1)c3ccc(cc3)c4ccc(OCCC5CCNCC5)cc4 | 5.22 |
| CHEMBL3677781 | Cc1cc(C(=O)O)c2[nH]c(nc2c1)c3c(F)c(F)c(c(F)c3F)c4ccc(O)cc4 | 7.8 |
| CHEMBL3677782 | Cc1cc(C(=O)O)c2c(c1)nc(c3ccc(cc3)c4ccc(O)cc4)n2CCO | 5.67 |
| CHEMBL3677783 | CONC(=O)c1cc(C)cc2nc([nH]c12)c3c(F)c(F)c(c(F)c3F)c4ccccc4 | 5.66 |
| CHEMBL3677784 | Cc1cc(C(=O)O)c2[nH]c(nc2c1)c3ccc(cc3)c4ccc(OCCO)cc4 | 5.51 |
| CHEMBL3677785 | Cc1cc(C(=O)O)c2[nH]c(nc2c1)c3c(F)c(F)c(c(F)c3F)c4ccccc4OCc5ccccc5 | 7.22 |
| CHEMBL3677787 | Cc1cc(C(=O)N)c2nc([nH]c2c1)c3c(F)c(F)c(c(F)c3F)c4ccccc4 | 6.35 |
| CHEMBL3677788 | Cc1cc(c2nnn[nH]2)c3nc([nH]c3c1)c4c(F)c(F)c(c(F)c4F)c5ccccc5 | 5.92 |
| CHEMBL3677789 | Cc1cc(C(=O)O)c2[nH]c(nc2c1)c3c(F)c(F)c(c(F)c3F)c4cccc(O)c4 | 7.87 |
| CHEMBL3677790 | Cc1cc(C(=O)O)c2[nH]c(nc2c1)c3c(F)c(F)c(c(F)c3F)c4cccc(OCc5ccccc5)c4 | 7.87 |
| CHEMBL3677791 | Cc1cc(C(=O)O)c2[nH]c(nc2c1)c3ccc(cc3)c4ccccc4OCc5ccccc5 | 5.82 |
| CHEMBL3677792 | OC(=O)c1cccc2nc([nH]c12)c3c(F)c(F)c(c(F)c3F)c4cccc(OCc5ccccc5)c4 | 7.58 |
| CHEMBL3677793 | Nc1nnc(s1)c2cccc3[nH]c(nc23)c4ccc(cc4)c5ccccc5 | 4.8 |
| CHEMBL3677794 | Cc1cc(C#N)c2nc([nH]c2c1)c3c(F)c(F)c(c(F)c3F)c4ccccc4 | 5.78 |
| CHEMBL3677795 | Cc1ccc(C)n1c2c(F)c(F)c(c(F)c2F)c3nc4cccc(C(=O)O)c4[nH]3 | 6.96 |
| CHEMBL3677796 | Cc1cc(C(=O)O)c2nc([nH]c2c1)c3ccc(cc3)c4cnn(Cc5ccccc5)c4 | 6.81 |
| CHEMBL3677798 | Cc1cc(C(=O)O)c2nc(c3c(F)c(F)c(c(F)c3F)c4ccccc4)n(C)c2c1 | 6.94 |
| CHEMBL3677799 | Cc1cc(C(=O)O)c2nc([nH]c2c1)c3ccc(cc3)c4ccc(OCc5ccncc5)cc4 | 5.76 |
| CHEMBL3677800 | Cc1cc(C(=O)O)c2nc([nH]c2c1)c3ccc(cc3)c4cccc(OCc5ccccc5)c4 | 5.92 |
| CHEMBL3677801 | Cc1cc(C(=O)O)c2nc([nH]c2c1)c3ccc(cc3)c4cccc(O)c4 | 6.06 |
| CHEMBL3677802 | Cc1cc(C(=O)O)c2nc([nH]c2c1)c3ccc(cc3)c4ccc(O)cc4 | 6.46 |
| CHEMBL3677803 | OC(=O)c1cccc2[nH]c(nc12)c3ccc(cc3)c4cccc(OCc5ccccc5)c4 | 5.89 |
| CHEMBL3677804 | Cc1cc(C(=O)O)c2nc([nH]c2c1)c3ccc(cc3)c4ccc(OCc5ccccn5)cc4 | 5.65 |
| CHEMBL3677805 | Cc1cc(C(=O)O)c2nc([nH]c2c1)c3ccc(cc3)c4ccc(OCc5cccnc5)cc4 | 6.12 |
| CHEMBL3677806 | Cc1cc(C(=O)O)c2[nH]c(nc2c1)c3ccc(cc3)n4c(C)ccc4C | 6.4 |
| CHEMBL3677807 | OC(=O)c1cccn2nc(nc12)c3ccc(cc3)c4ccccc4 | 5.92 |
| CHEMBL3677808 | Cc1cc(C(=O)O)c2c(c1)nc(c3c(F)c(F)c(c(F)c3F)c4ccccc4)n2C | 8.52 |
| CHEMBL3677809 | OC(=O)c1cccc2nc([nH]c12)c3c(F)c(F)c(c(F)c3F)c4cccs4 | 6.3 |
| CHEMBL3677810 | OC(=O)c1cccc2nc([nH]c12)c3c(F)c(F)c(c(F)c3F)c4cccc(F)c4 | 7.55 |
| CHEMBL3677811 | Cc1cc(C(=O)O)c2[nH]c(nc2c1)c3c(F)c(F)c(c(F)c3F)c4cccc(F)c4 | 8.05 |
| CHEMBL3677812 | Cc1cc(C(=O)O)c2nc([nH]c2c1)c3c(F)c(F)c(c(F)c3F)c4ccc(OCc5ccccc5)cc4 | 7.32 |

| | | |
|---|---|---|
| CHEMBL3677813 | OC(=O)c1cccc2[nH]c(nc12)c3c(F)c(F)c(c(F)c3F)c4cccc(F)c4F | 6.68 |
| CHEMBL3677814 | OC(=O)c1cccc2[nH]c(nc12)c3c(F)c(F)c(c(F)c3F)c4cc(F)cc(F)c4 | 7.7 |
| CHEMBL3677815 | OC(=O)c1cccc2[nH]c(nc12)c3c(F)c(F)c(c(F)c3F)c4ccc(F)c(F)c4 | 5.91 |
| CHEMBL3677816 | OC(=O)c1cccc2[nH]c(nc12)c3c(F)c(F)c(c(F)c3F)c4ccccc4F | 7.75 |
| CHEMBL3677817 | OC(=O)c1cccc2[nH]c(nc12)c3c(F)c(F)c(c(F)c3F)c4ccc(OCc5ccccc5)cc4 | 6.42 |
| CHEMBL3677818 | OC(=O)c1cccc2[nH]c(nc12)c3c(F)c(F)c(c(F)c3F)c4ccc(F)cc4 | 6.09 |
| CHEMBL3677819 | Cc1cc(C(=O)O)c2nc([nH]c2c1)c3c(F)c(F)c(c(F)c3F)c4ccc(F)cc4 | 6.93 |
| CHEMBL3677820 | Cc1cc(C(=O)O)c2nc([nH]c2c1)c3ccc(cc3)c4ccc(Oc5ccc(C=O)cc5)cc4 | 5.8 |
| CHEMBL3677821 | OC(=O)c1cccc2[nH]c(nc12)c3c(F)c(F)c(c(F)c3F)c4cccnc4 | 6.55 |
| CHEMBL3677822 | Cc1cc(C(=O)O)c2nc([nH]c2c1)c3ccc(cc3)c4ccc(Oc5ccccc5C(=O)O)cc4 | 5.65 |
| CHEMBL3677823 | OC(=O)c1cccn2cc(nc12)c3ccc(cc3)c4ccccc4 | 5.42 |
| CHEMBL3677824 | Cc1cc(C(=O)O)c2nc([nH]c2c1)c3c(F)c(F)c(c(F)c3F)c4ccsc4 | 7.3 |
| CHEMBL3677825 | Cc1cc(C(=O)O)c2nc([nH]c2c1)c3c(F)c(F)c(c(F)c3F)c4cccnc4 | 7.5 |
| CHEMBL3677826 | Cc1cc(C(=O)O)c2nc([nH]c2c1)c3ccc(cc3)c4ccc(Oc5ccccc5C=O)cc4 | 5.98 |
| CHEMBL3677827 | Cc1cc(C(=O)O)c2nc([nH]c2c1)c3ccc(cc3)c4ccc(OCc5ccccc5)cc4 | 5.91 |
| CHEMBL3677828 | OC(=O)c1cccc2[nH]c(nc12)c3c(F)c(F)c(c(F)c3F)c4ccsc4 | 6.36 |
| CHEMBL3677829 | Cc1cc(C(=O)O)c2nc([nH]c2c1)c3c(F)c(F)c(c(F)c3F)c4ccc(F)c(F)c4 | 6.5 |
| CHEMBL3677830 | Cc1cc(C(=O)O)c2nc([nH]c2c1)c3c(F)c(F)c(c(F)c3F)c4cc(F)cc(F)c4 | 7.68 |
| CHEMBL3677831 | Cc1cc(C(=O)O)c2nc([nH]c2c1)c3ccc(cc3)c4cnccn4 | 5.07 |
| CHEMBL3677832 | OC(=O)c1cccc2[nH]c(nc12)c3c(F)c(F)c(c(F)c3F)c4cccc(OC(F)(F)F)c4 | 6.66 |
| CHEMBL3677833 | Cc1cc(C(=O)O)c2nc([nH]c2c1)c3ccc(cc3)c4cccs4 | 5.78 |
| CHEMBL3694254 | O=C(NC1CC1)c2ccc(s2)N3CCSc4ccccc34 | 5.1 |
| CHEMBL370008 | OC(=O)C1=C(CCC1)C(=O)Nc2ccc(OCc3ccccc3)cc2 | 5.7 |
| CHEMBL370228 | COc1cccc(c1)c2ccc(NC(=O)c3cocc3C(=O)O)c(F)c2 | 6.47 |
| CHEMBL370865 | OC(=O)c1ccccc1NC(=O)c2ccc(cc2)c3ccccc3 | 5.3 |
| CHEMBL371732 | COc1cccc(c1)c2ccc(NC(=O)c3ccsc3C(=O)O)c(F)c2 | 7.36 |
| CHEMBL372101 | OC(=O)C1=C(CSC1)C(=O)Nc2ccc(cc2)c3ccccc3 | 6.18 |
| CHEMBL374432 | Cc1ccc2c(C(=O)O)c(O)c(nc2c1C)c3ccc(Br)cc3 | 4.25 |
| CHEMBL375425 | Cc1cc(C)c2nc(c(O)c(C(=O)O)c2c1)c3ccc(Cl)cc3 | 5.31 |
| CHEMBL3808600 | COc1ccccc1NC(=O)c2cccc3nc([nH]c23)c4cc(Br)nn4c5ncccc5Cl | 6.75 |
| CHEMBL3809369 | Cc1ccccc1NC(=O)c2cccc3nc([nH]c23)c4cc(Br)nn4c5ncccc5Cl | 6.17 |
| CHEMBL3809712 | COc1ccc(cc1NC(=O)c2cccc3nc([nH]c23)c4cc(Br)nn4c5ncccc5Cl)C(F)(F)F | 5.31 |
| CHEMBL381043 | OC(=O)c1sccc1C(=O)Nc2ccc(cc2)c3ccccc3 | 6.52 |
| CHEMBL3817894 | CCCCOc1cccc(c1)c2cc(C(=O)O)c3ccccc3n2 | 5.88 |
| CHEMBL3818031 | CCC(C)c1ccc(cc1)c2nc3ccc(Br)cc3c(C(=O)O)c2C | 8.01 |
| CHEMBL3818174 | COC(=O)c1cc(nc2ccccc12)c3ccc(OC)c(OC)c3 | 5.49 |
| CHEMBL3818257 | CC(C)c1ccc(cc1)c2cc(C(=O)O)c3cc(Br)ccc3n2 | 6.87 |
| CHEMBL3818502 | CCCCc1ccc(cc1)c2cc(C(=O)O)c3ccccc3n2 | 6.36 |
| CHEMBL3818640 | OC(=O)c1cc(nc2ccc(Br)cc12)c3ccc(Cl)c(Cl)c3 | 7.24 |
| CHEMBL3819188 | OC(=O)c1cc(nc2ccc(Br)cc12)c3ccc(Cl)cc3Cl | 6.18 |
| CHEMBL383165 | CCOc1ccccc1c2cc(F)c(NC(=O)c3cscc3C(=O)O)c(F)c2 | 8 |
| CHEMBL38434 | Cc1c(C(=O)O)c2cc(F)ccc2nc1c3ccc(cc3)c4ccccc4F | 7.4 |
| CHEMBL386159 | OC(=O)c1c(O)c(nc2ccc(F)cc12)c3ccc(cc3)c4ccc(O)cc4 | 8.15 |
| CHEMBL386700 | OC(=O)c1c(O)c(nc2ccc(cc12)C(F)(F)F)c3ccc(Cl)cc3 | 6.57 |

| | | |
|---|---|---|
| CHEMBL3891218 | Cc1sc(N\N=C\c2ccccc2C(=O)O)nc1c3ccccc3Cl | 7.18 |
| CHEMBL3893343 | CCCC(C)N(\N=C\c1ccccc1C(=O)O)c2nc(c(C)s2)c3ccccc3Cl | 7.82 |
| CHEMBL3896992 | OC(=O)c1ccccc1\C=N\Nc2nc(cs2)c3ccccc3Cl | 7.54 |
| CHEMBL3900235 | OCCN(\N=C\c1ccccc1C(=O)O)c2nc(cs2)c3ccccc3Cl | 6.37 |
| CHEMBL3907085 | O=C(CCNC(=O)c1ccccc1)N[C@@H]2CCCc3ccccc23 | 4.91 |
| CHEMBL3909259 | CCCN(\N=C\c1ccccc1C(=O)O)c2nc(cs2)c3ccccc3Cl | 8.05 |
| CHEMBL3910373 | CCN(\N=C\c1ccccc1C(=O)O)c2nc(cs2)c3ccccc3Cl | 7.23 |
| CHEMBL3913950 | CN(\N=C\c1ccccc1C(=O)O)c2nc(cs2)c3cccc(Cl)c3 | 7.96 |
| CHEMBL3914938 | OC(=O)c1cccnc1Nc2ccc(OC(F)(F)F)cc2 | 6.3 |
| CHEMBL3916793 | OC(=O)c1cccnc1Nc2ccc(F)cc2 | 5.55 |
| CHEMBL3919303 | CCc1sc(N\N=C\c2ccccc2C(=O)O)nc1c3ccccc3Cl | 6.45 |
| CHEMBL3922925 | CN(\N=C\c1ccc(C)cc1C(=O)O)c2nc(cs2)c3ccccc3Cl | 8.1 |
| CHEMBL3927131 | CCCC(C)N(\N=C\c1ccccc1C(=O)O)c2nc(cs2)c3ccccc3Cl | 7.72 |
| CHEMBL3928205 | CCC(C)N(\N=C\c1ccccc1C(=O)O)c2nc(cs2)c3ccccc3Cl | 7.62 |
| CHEMBL3931872 | CN(\N=C\c1ccc(C)cc1C(=O)O)c2nc(c(C)s2)c3ccccc3 | 7.85 |
| CHEMBL3937185 | CC(C)N(\N=C\c1ccccc1C(=O)O)c2nc(cs2)c3ccccc3Cl | 7.03 |
| CHEMBL3941922 | CN(\N=C\c1ccccc1C(=O)O)c2nc(cs2)c3cc(Cl)ccc3Cl | 7.55 |
| CHEMBL3944693 | OC(=O)c1cccnc1Nc2ccccc2 | 4.76 |
| CHEMBL3949527 | OC(=O)c1ccccc1\C=N\Nc2nc(cs2)c3ccccc3 | 6.86 |
| CHEMBL3950655 | CN(\N=C\c1ccc(cc1C(=O)O)C(F)(F)F)c2nc(cs2)c3ccccc3Cl | 7.06 |
| CHEMBL3959360 | CN(\N=C\c1ccc(F)cc1C(=O)O)c2nc(cs2)c3ccccc3Cl | 8.7 |
| CHEMBL3968151 | Cc1sc(N\N=C\c2ccccc2C(=O)O)nc1c3ccccc3 | 7.09 |
| CHEMBL3980486 | CN(\N=C\c1ccccc1C(=O)O)c2nc(cs2)c3ccccc3Cl | 8.1 |
| CHEMBL41719 | OC(=O)c1c(O)c(nc2ccc(F)cc12)c3ccc(cc3)c4ccccc4 | 8.3 |
| CHEMBL418870 | FC(F)(F)c1ccc(Nc2[nH]nc3CCCC(=O)c23)cc1 | 5.19 |
| CHEMBL419631 | CCc1oncc1\C(=N\c2ccc(cc2)N(C)C)\S | 4.39 |
| CHEMBL483161 | COc1ccccc1c2ccc(NC(=O)\C(=C(\C)/O)\C#N)c(Cl)c2 | 6.7 |
| CHEMBL483552 | C\C(=C(/C#N)\C(=O)Nc1ccc(cc1Cl)c2ccccc2Cl)\O | 6.75 |
| CHEMBL483994 | C\C(=C(/C#N)\C(=O)Nc1ccc(cc1Cl)c2ccccc2)\O | 6.5 |
| CHEMBL483995 | CCOc1cccc(c1)c2ccc(NC(=O)\C(=C(\C)/O)\C#N)cc2 | 6.89 |
| CHEMBL484531 | C\C(=C(/C#N)\C(=O)Nc1ccc(cc1)c2ccccc2Cl)\O | 6.89 |
| CHEMBL484594 | C\C(=C(/C#N)\C(=O)Nc1ccc(cc1Cl)c2c(Cl)cccc2Cl)\O | 4.78 |
| CHEMBL484595 | C\C(=C(/C#N)\C(=O)Nc1ccc(cc1Cl)c2cccc(Cl)c2)\O | 5.82 |
| CHEMBL484602 | C\C(=C(/C#N)\C(=O)Nc1ccc(cc1C(F)(F)F)c2cccc(Cl)c2)\O | 4.06 |
| CHEMBL484780 | C\C(=C(/C#N)\C(=O)Nc1ccc(c(Cl)c1)c2ccccc2Cl)\O | 7.66 |
| CHEMBL505315 | C\C(=C(/C#N)\C(=O)Nc1ccc(cc1)c2cccc(Cl)c2Cl)\O | 6.7 |
| CHEMBL519160 | C\C(=C(/C#N)\C(=O)Nc1ccc(cc1)c2ccccc2)\O | 7.05 |
| CHEMBL520008 | C\C(=C(/C#N)\C(=O)Nc1ccc(c(Cl)c1)c2cccc(Cl)c2)\O | 6.72 |
| CHEMBL520306 | C\C(=C(/C#N)\C(=O)Nc1ccc(cc1C(F)(F)F)c2ccccc2Cl)\O | 6.77 |
| CHEMBL520999 | C\C(=C(/C#N)\C(=O)Nc1ccc(cc1Cl)c2cccc(Cl)c2Cl)\O | 6.48 |
| CHEMBL571285 | NC(=O)c1cccc2[nH]c(nc12)c3ccc(cc3)c4cccnc4 | 4.72 |
| CHEMBL576423 | NC(=O)c1cccc2[nH]c(nc12)c3ccc(cc3)c4ccccn4 | 4.55 |
| CHEMBL578185 | NC(=O)c1cccc2[nH]c(nc12)c3ccc(cc3)c4ccccc4 | 5.01 |
| CHEMBL63323 | OC(=O)c1cccnc1Nc2cccc(c2)C(F)(F)F | 4.77 |

| | | |
|---|---|---|
| CHEMBL70261 | Cc1oncc1\C(=N\c2ccc(OC(F)(F)F)cc2)\S | 5.14 |
| CHEMBL70544 | CC(=O)\C(=C(\S)/Nc1ccc(cc1)C(F)(F)F)\C#N | 6.1 |
| CHEMBL70708 | FC(F)(F)c1ccc(NC(=S)c2cnoc2C3CC3)cc1 | 5.5 |
| CHEMBL71154 | CCc1oncc1\C(=N\c2ccc(cc2)C#N)\S | 4.96 |
| CHEMBL960 | Cc1oncc1C(=O)Nc2ccc(cc2)C(F)(F)F | 5 |
| CHEMBL973 | C\C(=C(/C#N)\C(=O)Nc1ccc(cc1)C(F)(F)F)\O | 6.86 |
| CHEMBL999 | C\C(=C(\C#N)/C(=O)Nc1ccc(cc1)C(F)(F)F)\O | 6.36 |

**Table S2.** Docking score and predicted activity value for the top 200 zinc candidates.

| Name | Docking score | Predicted activity | | |
|---|---|---|---|---|
| | | GIAN | GIAT | SGCA |
| ZINC000008577218* | 64.296 | 7.300996 | 7.765394 | 7.234998 |
| ZINC000015919406 | 63.009 | 6.723702 | 5.593675 | 6.857596 |
| ZINC000002036915 | 62.93 | 7.608401 | 7.819659 | 6.585709 |
| ZINC000003952167 | 62.463 | 6.645174 | 6.335661 | 5.166162 |
| ZINC000043100953 | 61.858 | 4.887485 | 7.542762 | 7.400549 |
| ZINC000003831490 | 61.751 | 6.62721 | 7.416915 | 7.190752 |
| ZINC000004261765* | 60.522 | 8.057562 | 10.81596 | 12.78676 |
| ZINC000001530605 | 60.384 | 5.740261 | 7.979621 | 6.135328 |
| ZINC000001587572 | 59.994 | 7.6084 | 7.81966 | 6.585708 |
| ZINC000095618747* | 59.621 | 7.532437 | 10.96646 | 12.39158 |
| ZINC000043195321 | 59.17 | 6.07048 | 7.23513 | 5.990883 |
| ZINC000100004249 | 59.033 | 5.674796 | 6.083844 | 5.279045 |
| ZINC000100048501 | 58.625 | 6.299141 | 8.085845 | 6.764382 |
| ZINC000013588928 | 58.578 | 7.530169 | 8.905917 | 10.66835 |
| ZINC000095892888 | 58.467 | 4.630929 | 5.935944 | 5.206252 |
| ZINC000022054044 | 58.284 | 6.194102 | 7.258717 | 6.175798 |
| ZINC000006095847 | 58.118 | 5.294021 | 6.119356 | 6.854791 |
| ZINC000001444556 | 58.108 | 5.074522 | 6.087246 | 5.250934 |
| ZINC000000005423 | 58.018 | 5.96597 | 7.224964 | 6.556073 |
| ZINC000001540228 | 57.612 | 6.668216 | 6.510778 | 7.344737 |
| ZINC000043194409 | 57.499 | 6.707047 | 4.910901 | 5.83283 |
| ZINC000000089688 | 57.233 | 5.00581 | 5.799558 | 5.621189 |
| ZINC000003871698 | 57.183 | 5.491028 | 6.483674 | 5.680937 |
| ZINC000003872687 | 56.959 | 4.598122 | 5.705404 | 5.326142 |
| ZINC000053166256 | 56.936 | 4.977914 | 4.997481 | 5.279971 |
| ZINC000033753205 | 56.9 | 5.868948 | 9.044409 | 10.17845 |
| ZINC000000007455 | 56.757 | 4.696512 | 6.164524 | 5.058284 |
| ZINC000001493878 | 56.466 | 6.399541 | 8.024447 | 7.691423 |
| ZINC000100005073 | 56.312 | 4.23337 | 7.947003 | 5.665305 |
| ZINC000019632628 | 56.269 | 5.228067 | 4.575965 | 5.482564 |
| ZINC000000121541 | 56.257 | 4.744668 | 4.305178 | 3.71702 |
| ZINC000013129998 | 56.23 | 4.986147 | 5.606158 | 5.192493 |
| ZINC000000537795 | 56.195 | 5.679065 | 6.292289 | 6.575707 |
| ZINC000012501706 | 56.083 | 7.57146 | 8.986579 | 11.4689 |
| ZINC000019203855 | 55.963 | 4.09478 | 6.272138 | 5.492144 |
| ZINC000001530935 | 55.905 | 4.633284 | 3.962843 | 4.647081 |
| ZINC000003952881 | 55.842 | 5.451977 | 6.315676 | 6.638064 |
| ZINC000004618208 | 55.713 | 4.633284 | 3.962843 | 4.647081 |
| ZINC000001530604 | 55.606 | 5.740261 | 7.979621 | 6.135327 |
| ZINC000000001342 | 55.487 | 4.446496 | 4.897518 | 4.607798 |
| ZINC000002040778 | 55.399 | 4.971159 | 4.704404 | 5.306667 |

| | | | | |
|---|---|---|---|---|
| ZINC000017835656 | 55.256 | 3.994599 | 4.564504 | 3.85613 |
| ZINC000003830847 | 55.14 | 4.240569 | 5.403502 | 4.173788 |
| ZINC000003874496 | 54.744 | 4.696104 | 5.328797 | 5.245481 |
| ZINC000000607910 | 54.623 | 4.598122 | 5.705404 | 5.326142 |
| ZINC000003956788 | 54.564 | 5.213435 | 5.50495 | 5.163043 |
| ZINC000001999441 | 54.309 | 7.473666 | 8.909299 | 7.538009 |
| ZINC000003860441 | 54.142 | 4.760465 | 8.037248 | 6.951668 |
| ZINC000022056375 | 54.133 | 5.262184 | 5.959076 | 5.878389 |
| ZINC000006093393 | 53.857 | 4.281672 | 5.780708 | 5.956861 |
| ZINC000006745272 | 53.717 | 5.595707 | 7.352758 | 7.356881 |
| ZINC000005423072 | 53.699 | 4.642952 | 5.160971 | 5.682736 |
| ZINC000005843546 | 53.657 | 5.580665 | 6.001852 | 5.799144 |
| ZINC000005140767 | 53.651 | 4.668482 | 6.256027 | 4.896819 |
| ZINC000003935481 | 53.651 | 4.439359 | 5.15712 | 5.499027 |
| ZINC000000538174 | 53.631 | 5.713495 | 6.447265 | 6.845958 |
| ZINC000001843099 | 53.585 | 5.767795 | 6.613459 | 4.489143 |
| ZINC000007997905 | 53.318 | 3.994599 | 4.564504 | 3.85613 |
| ZINC000000001567 | 53.313 | 4.715746 | 5.611708 | 5.534105 |
| ZINC000005425173 | 53.285 | 4.349578 | 5.766132 | 4.529187 |
| ZINC000003791775 | 53.242 | 4.410463 | 6.007408 | 4.677265 |
| ZINC000005733652 | 52.942 | 4.79531 | 4.110694 | 5.426235 |
| ZINC000000538163 | 52.926 | 5.043701 | 6.729642 | 5.478788 |
| ZINC000017653974 | 52.92 | 5.4334 | 5.617358 | 5.085844 |
| ZINC000003964126 | 52.916 | 4.845099 | 6.078844 | 6.276227 |
| ZINC000000057466 | 52.859 | 5.778152 | 6.512209 | 4.772442 |
| ZINC000007997966 | 52.811 | 4.578921 | 5.517211 | 5.383073 |
| ZINC000252679615 | 52.811 | 4.57892 | 5.517211 | 5.383073 |
| ZINC000002568036 | 52.811 | 4.21554 | 5.479602 | 5.626502 |
| ZINC000000899824 | 52.81 | 6.183718 | 6.968305 | 6.779979 |
| ZINC000009060429 | 52.787 | 4.410463 | 6.007408 | 4.677264 |
| ZINC000013546270 | 52.778 | 8.218697 | 10.84782 | 11.66157 |
| ZINC000013449412 | 52.76 | 5.485429 | 5.467895 | 6.842892 |
| ZINC000000035804 | 52.746 | 5.749037 | 6.361116 | 6.146009 |
| ZINC000005140766 | 52.66 | 4.950457 | 4.979789 | 5.379768 |
| ZINC000004099200 | 52.523 | 4.849636 | 5.701288 | 5.550549 |
| ZINC000019632614 | 52.519 | 6.488966 | 7.526567 | 6.908721 |
| ZINC000003818726 | 52.508 | 5.65894 | 6.333538 | 5.042331 |
| ZINC000100015491 | 52.447 | 4.661204 | 4.851078 | 5.705404 |
| ZINC000000599734 | 52.355 | 4.895085 | 4.47099 | 4.89599 |
| ZINC000028870000 | 52.253 | 6.107962 | 5.08248 | 5.52845 |
| ZINC000001481956 | 52.145 | 6.695495 | 7.295831 | 7.462146 |
| ZINC000051133897 | 52.084 | 4.6772 | 6.337007 | 4.672034 |
| ZINC000022060383 | 52.08 | 6.080103 | 6.294571 | 6.257107 |
| ZINC000000000865 | 52.021 | 4.592355 | 5.138213 | 4.581967 |

| | | | | |
|---|---|---|---|---|
| ZINC000019203852 | 52.011 | 4.09478 | 6.272138 | 5.492144 |
| ZINC000012404515 | 51.945 | 5.975027 | 5.010041 | 5.654284 |
| ZINC000004214700 | 51.876 | 6.695495 | 7.295831 | 7.462146 |
| ZINC000001640621 | 51.81 | 4.510243 | 5.09842 | 4.939918 |
| ZINC000013531944 | 51.778 | 5.88609 | 5.933717 | 6.081086 |
| ZINC000000968326 | 51.773 | 4.828036 | 5.009841 | 5.750008 |
| ZINC000001530811 | 51.772 | 4.433525 | 4.042876 | 4.344839 |
| ZINC000000607986 | 51.659 | 7.473666 | 8.909299 | 7.538009 |
| ZINC000013233295 | 51.592 | 5.339896 | 5.632687 | 5.314468 |
| ZINC000000601305 | 51.543 | 4.68365 | 4.094828 | 5.014022 |
| ZINC000100006429 | 51.337 | 4.933535 | 5.193223 | 4.915394 |
| ZINC000012341529 | 51.318 | 5.886089 | 5.933717 | 6.081086 |
| ZINC000013831791 | 51.317 | 5.438772 | 5.346507 | 5.247869 |
| ZINC000029210629 | 51.312 | 6.777005 | 6.920874 | 6.221011 |
| ZINC000003786192 | 51.295 | 6.115422 | 5.175305 | 5.774267 |
| ZINC000001641925 | 51.29 | 4.729214 | 5.231195 | 4.858466 |
| ZINC000022056370 | 51.25 | 5.262183 | 5.959076 | 5.878389 |
| ZINC000043202140 | 51.188 | 6.82178 | 6.980927 | 6.892757 |
| ZINC000000001003 | 51.149 | 4.847857 | 6.034954 | 5.110968 |
| ZINC000011679756 | 51.109 | 7.149884 | 7.044483 | 6.112609 |
| ZINC000000968328 | 50.955 | 5.15685 | 5.867417 | 5.734884 |
| ZINC000004096488 | 50.94 | 4.927535 | 7.252732 | 8.655126 |
| ZINC000001545565 | 50.853 | 5.395932 | 4.364805 | 4.547904 |
| ZINC000001530940 | 50.834 | 4.326652 | 4.745024 | 4.453138 |
| ZINC000003927870 | 50.825 | 4.183327 | 7.745678 | 8.274746 |
| ZINC000004098610 | 50.796 | 5.824383 | 8.410183 | 7.736589 |
| ZINC000000000017 | 50.728 | 4.138582 | 4.407006 | 3.364537 |
| ZINC000000968327 | 50.627 | 4.828036 | 5.00984 | 5.750008 |
| ZINC000000538538 | 50.545 | 4.491793 | 4.647077 | 4.487322 |
| ZINC000004676424 | 50.413 | 4.849636 | 5.701287 | 5.550549 |
| ZINC000005424275 | 50.39 | 3.85517 | 5.024442 | 3.435555 |
| ZINC000000057461 | 50.346 | 5.594043 | 5.873698 | 4.596666 |
| ZINC000004097476 | 50.343 | 3.716354 | 5.61807 | 4.287302 |
| ZINC000031425359 | 50.334 | 7.051373 | 5.293667 | 8.015163 |
| ZINC000000002094 | 50.332 | 5.203577 | 5.116295 | 5.516985 |
| ZINC000034220093 | 50.33 | 4.455754 | 4.289412 | 4.944213 |
| ZINC000040899447 | 50.312 | 6.536111 | 7.885767 | 6.95361 |
| ZINC000033972992 | 50.064 | 6.967649 | 7.201378 | 7.552161 |
| ZINC000004475353 | 50.032 | 6.115422 | 5.175305 | 5.774268 |
| ZINC000000592419 | 50.013 | 4.756728 | 6.511214 | 6.597853 |
| ZINC000000057417 | 49.999 | 5.892313 | 5.490169 | 4.702092 |
| ZINC000003860156 | 49.89 | 4.854493 | 7.119677 | 8.315127 |
| ZINC000035328014 | 49.862 | 6.649402 | 6.070032 | 5.88962 |
| ZINC000003816292 | 49.726 | 4.712955 | 6.305059 | 4.91292 |

| | | | | |
|---|---|---|---|---|
| ZINC000000538312 | 49.718 | 6.230903 | 7.258238 | 7.221371 |
| ZINC000013514109 | 49.679 | 4.375775 | 7.299162 | 8.380635 |
| ZINC000006030359 | 49.619 | 6.647312 | 6.379235 | 7.382363 |
| ZINC000000057493 | 49.604 | 4.367746 | 4.50208 | 4.420006 |
| ZINC000004095934 | 49.593 | 4.138582 | 4.407006 | 3.364537 |
| ZINC000017146904 | 49.58 | 3.739798 | 4.103144 | 3.139428 |
| ZINC000000643114 | 49.554 | 4.68365 | 4.094828 | 5.014022 |
| ZINC000000089763 | 49.549 | 4.304827 | 4.670278 | 4.862598 |
| ZINC000000057490 | 49.523 | 5.419512 | 5.9966 | 5.425169 |
| ZINC000018185774 | 49.513 | 5.646585 | 4.266383 | 5.801611 |
| ZINC000001530713 | 49.379 | 5.715982 | 7.472035 | 5.514945 |
| ZINC000000016154 | 49.277 | 5.088128 | 5.384476 | 4.578799 |
| ZINC000004693575 | 49.017 | 4.655963 | 5.358908 | 4.808446 |
| ZINC000000537802 | 48.977 | 5.317133 | 6.073922 | 6.325182 |
| ZINC000001542113 | 48.849 | 6.035281 | 5.986148 | 6.59202 |
| ZINC000000537805 | 48.815 | 6.089524 | 6.553387 | 6.552194 |
| ZINC000001540998 | 48.811 | 6.414423 | 6.278392 | 6.592865 |
| ZINC000000125047 | 48.764 | 5.52607 | 5.549182 | 5.075521 |
| ZINC000019796155 | 48.732 | 5.597431 | 5.292646 | 5.245823 |
| ZINC000003927198 | 48.727 | 4.424043 | 8.329219 | 5.211196 |
| ZINC000001690324 | 48.68 | 4.781328 | 4.565401 | 5.347376 |
| ZINC000005844788 | 48.639 | 7.473665 | 8.909299 | 7.538009 |
| ZINC000021981235 | 48.574 | 5.194707 | 5.071111 | 5.237409 |
| ZINC000003830961 | 48.506 | 3.184162 | 5.421502 | 3.932911 |
| ZINC000036056301 | 48.505 | 5.825628 | 4.481654 | 5.086045 |
| ZINC000003871356 | 48.388 | 3.501432 | 3.722906 | 4.610196 |
| ZINC000053084692 | 48.381 | 4.212025 | 4.502934 | 5.369962 |
| ZINC000000039092 | 48.338 | 4.841402 | 3.964946 | 5.051723 |
| ZINC000001530636 | 48.284 | 5.017924 | 5.41182 | 4.599806 |
| ZINC000005116154 | 48.278 | 4.517035 | 6.40187 | 4.764874 |
| ZINC000000537874 | 48.263 | 4.961668 | 6.04738 | 5.822662 |
| ZINC000000001246 | 48.259 | 5.434993 | 5.527972 | 6.044355 |
| ZINC000000025958 | 48.226 | 3.739155 | 5.109676 | 4.33611 |
| ZINC000013818943 | 48.201 | 4.500135 | 7.787578 | 7.226458 |
| ZINC000001530788 | 48.145 | 5.424422 | 6.936097 | 6.434423 |
| ZINC000049643479 | 48.093 | 5.287564 | 4.988073 | 5.870728 |
| ZINC000001489478 | 48.043 | 7.213428 | 8.071492 | 6.924459 |
| ZINC000006467621 | 47.996 | 3.467192 | 5.976681 | 4.785444 |
| ZINC000000968330 | 47.965 | 5.15685 | 5.867417 | 5.734885 |
| ZINC000003830933 | 47.907 | 3.956304 | 6.191349 | 4.583596 |
| ZINC000003814784 | 47.904 | 5.975027 | 5.010042 | 5.654285 |
| ZINC000000002097 | 47.876 | 4.750436 | 5.396768 | 5.246945 |
| ZINC000022059926 | 47.808 | 5.49988 | 5.974154 | 5.263975 |
| ZINC000005924265 | 47.762 | 5.09754 | 4.794147 | 5.356349 |

| | | | | |
|---|---|---|---|---|
| ZINC000003812865 | 47.704 | 5.815678 | 5.943406 | 5.403072 |
| ZINC000000057537 | 47.64 | 5.431703 | 4.983044 | 5.389072 |
| ZINC000000057494 | 47.539 | 4.579184 | 5.200683 | 4.814668 |
| ZINC000002005305 | 47.482 | 7.351001 | 8.238765 | 7.220576 |
| ZINC000000001490 | 47.463 | 4.907505 | 5.452746 | 4.464963 |
| ZINC000100004231 | 47.438 | 5.775934 | 6.06221 | 5.453905 |
| ZINC000000011012 | 47.418 | 4.493898 | 5.679562 | 5.331686 |
| ZINC000000900543 | 47.372 | 3.13424 | 4.511394 | 3.722074 |
| ZINC000035342789 | 47.369 | 5.131887 | 3.623349 | 5.206191 |
| ZINC000000898237 | 47.342 | 4.191215 | 4.989699 | 4.945329 |
| ZINC000003871576 | 47.328 | 5.756988 | 4.360831 | 5.81127 |
| ZINC000000005191 | 47.315 | 4.770481 | 3.731991 | 4.617854 |
| ZINC000004213946 | 47.234 | 7.473666 | 8.909299 | 7.538009 |
| ZINC000002570817 | 47.222 | 4.322212 | 3.962971 | 5.058281 |
| ZINC000004693574 | 47.216 | 4.655963 | 5.358908 | 4.808446 |
| ZINC000011617039 | 47.173 | 7.046603 | 7.681741 | 6.688553 |
| ZINC000003872177 | 47.115 | 8.41622 | 4.727499 | 7.164699 |
| ZINC000100004227 | 47.113 | 4.712177 | 4.765829 | 4.191784 |
| ZINC000095619124 | 47.066 | 6.739865 | 6.383723 | 7.551508 |
| ZINC000018115268 | 47.034 | 6.856761 | 5.478155 | 4.636524 |
| ZINC000000001382 | 47.018 | 4.378035 | 5.346808 | 4.750925 |
| ZINC000003869608 | 46.997 | 5.860081 | 5.261596 | 5.896521 |
| ZINC000003830986 | 46.987 | 4.895085 | 4.47099 | 4.89599 |
| ZINC000006716957 | 46.969 | 9.029577 | 8.046642 | 8.966531 |
| ZINC000095617673 | 46.882 | 5.123659 | 5.474619 | 6.76257 |
| ZINC000000020221 | 46.86 | 3.75293 | 3.763909 | 4.088777 |
| ZINC000001529323 | 46.856 | 7.434438 | 7.96345 | 7.010843 |

*The selected candidates

(a)

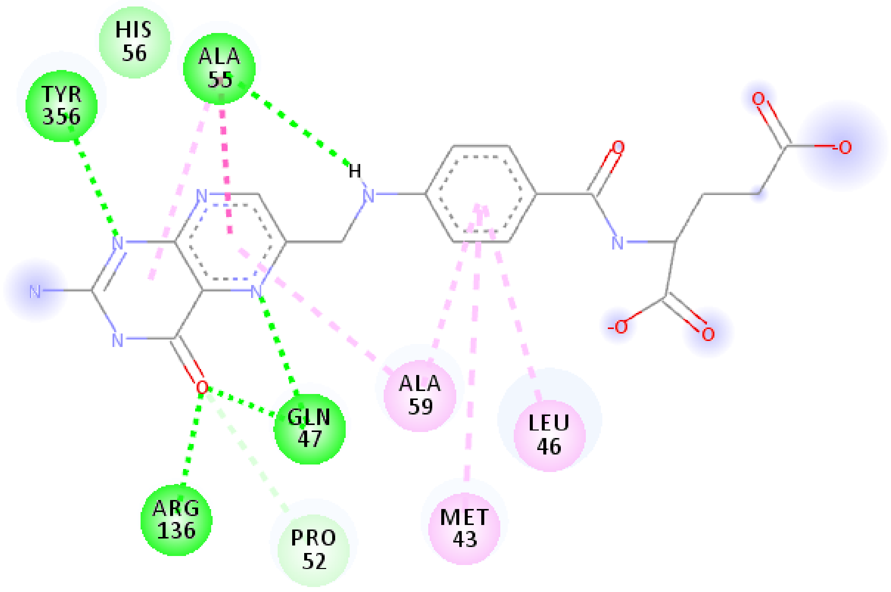
HIS 56
ALA 55
TYR 356
GLN 47
ARG 136
PRO 52
ALA 59
MET 43
LEU 46

(b)

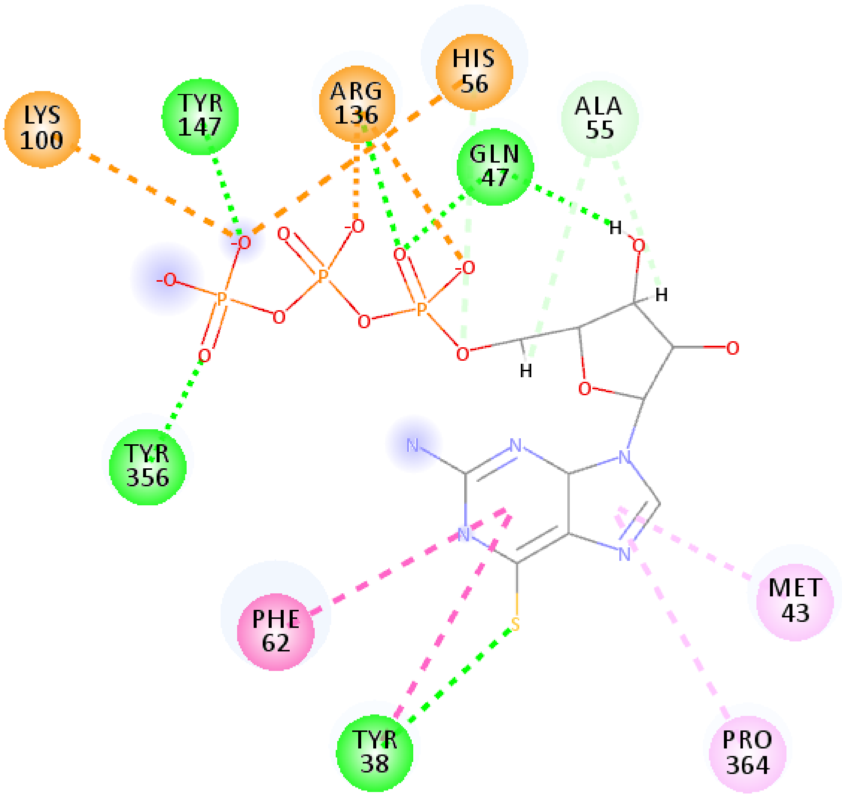
LYS 100
TYR 147
ARG 136
HIS 56
ALA 55
GLN 47
TYR 356
PHE 62
TYR 38
MET 43
PRO 364

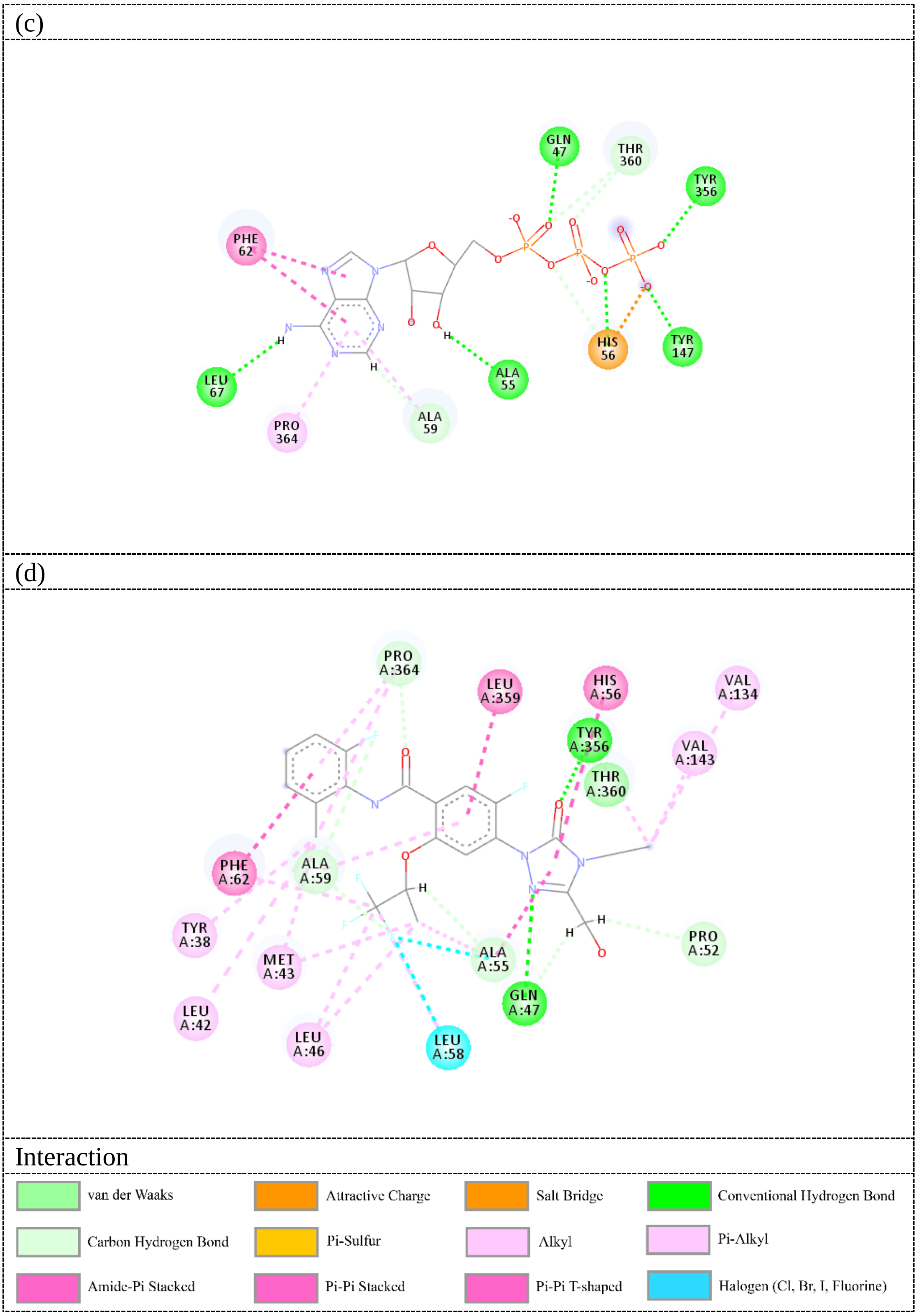


**Figure S1.** 2D horizon of molecular docking results. (**a**) ZINC8577218, (**b**) ZINC95618747, (**c**) ZINC4261765 and (**d**) BAY-2402234.

(a)

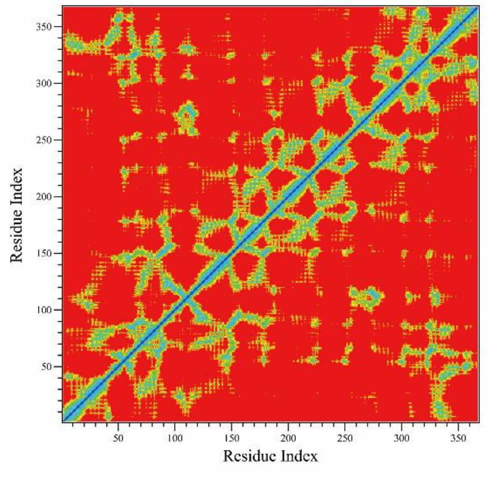

Residue Index
Residue Index


(b)

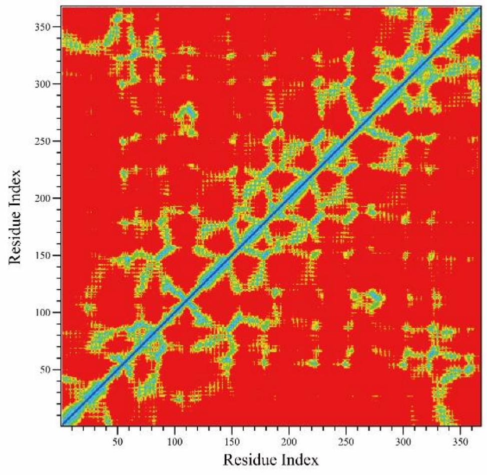

Residue Index
Residue Index


(c)

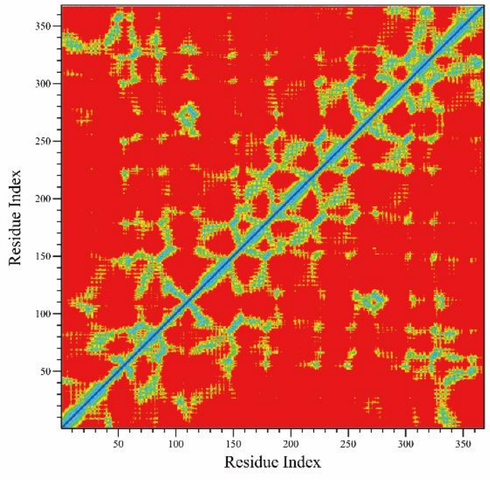

Residue Index
Residue Index


(d)

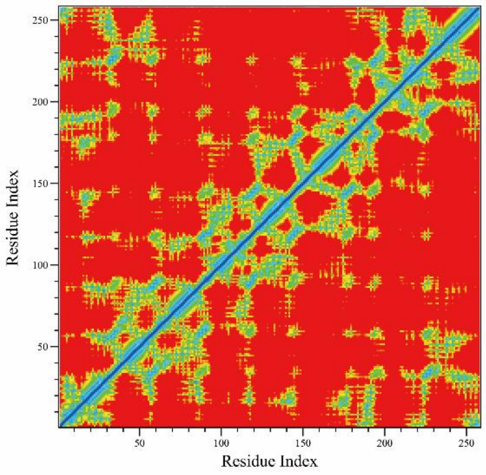

Residue Index
Residue Index

(e)

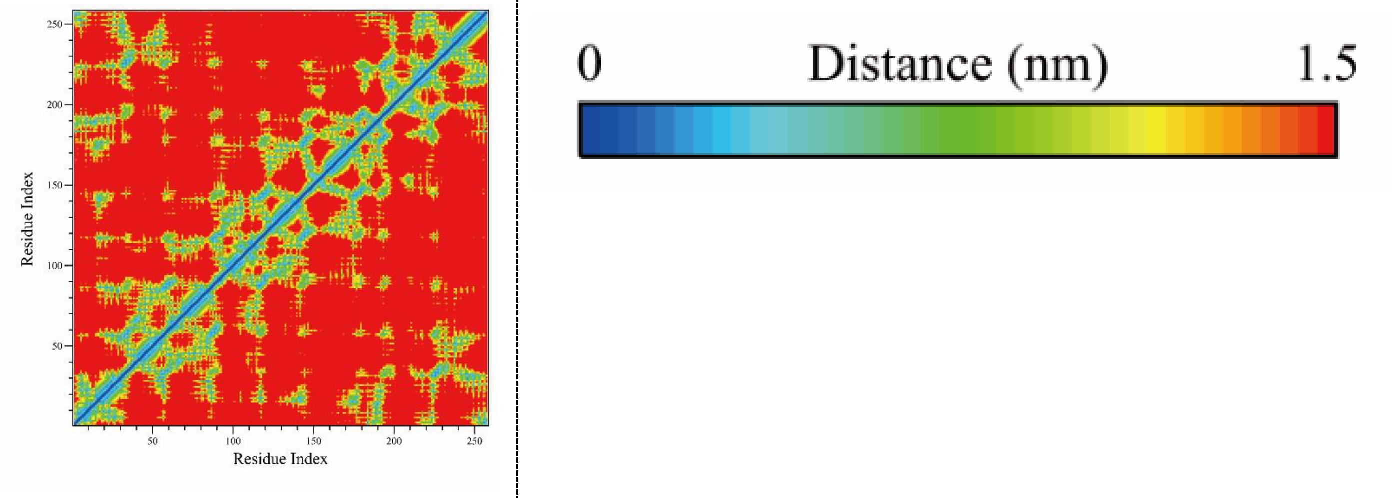


**Figure S2.** Residue distance matrix. (**a**) DHODH protein with ZINC8577218. (**b**) DHODH protein with ZINC95618747. (**c**) DHODH protein with ZINC4261765. (**d**) UMPS protein with ZINC95618747. (**e**) UMPS protein with ZINC4261765.
